# Design, Synthesis, Biological Evaluation, and Molecular Dynamics Simulation of Influenza Polymerase PB2 Inhibitors

**DOI:** 10.3390/molecules28041849

**Published:** 2023-02-15

**Authors:** Xinhong Li, Yijie Xu, Wei Li, Jinjing Che, Xu Zhao, Ruyuan Cao, Xingzhou Li, Song Li

**Affiliations:** 1 National Engineering Research Center for the Emergency Strategic Drug, Beijing Institute of Pharmacology and Toxicology, Beijing 100850, China; 2 Department of Hepatology, Fifth Medical Center of Chinese PLA General Hospital, Beijing 100039, China; 3 China Military Institute of Chinese Materia, Fifth Medical Center of Chinese PLA General Hospital, Beijing 100053, China

**Keywords:** influenza virus, antiviral, PB2 inhibitors, 2,3-dihydro-imidazopyridine, molecular dynamics, MM/PBSA

## Abstract

The PB2 subunit of the influenza RNA-dependent RNA polymerase (RdRp) has been identified as a promising target for the treatment of influenza. To expand the chemical space of the known influenza polymerase PB2 inhibitor–pimodivir (formerly VX-787) and improve its pharmacokinetic profile, two pimodivir analogs containing 2,3-dihydro-imidazopyridine fragment (comp. **I** and comp. **II**) were designed, synthesized, and evaluated for anti-influenza virus activity. In the cytopathic effect (CPE) inhibition assay, comp. **I** and comp. **II** showed IC_50_ values of 0.07 and 0.09 μM for A/Puerto Rico/8/34 (H1N1) and 0.04 and 0.07 μM for A/Hong Kong/8/68 (H3N2), respectively. Protein-binding affinity assay results showed a concentration-dependent association and dissociation pattern, with K_D_ values of 1.398 and 1.670 μM, respectively. In vitro metabolic stability assays showed that comp. **I** and comp. **II** exhibited good stability to liver microsomes and considerably less sensitivity to aldehyde oxidase compared to pimodivir. The binding modes of comp. **I** and comp. **II** were similar to those of VX-787; however, comp. **I** and comp. **II** had lower structural adaptability to PB2 than VX-787. Our results provide helpful information regarding the structure–activity relationship for the design of novel PB2 inhibitors and a reference for the development of drugs containing 2,3-dihydro-imidazopyridine fragments.

## 1. Introduction

**Figure 1:**
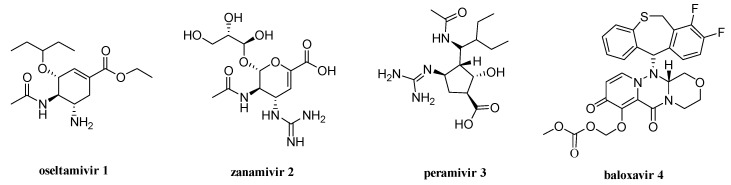
FDA-approved anti-flu drugs currently in use.

Influenza A virus (IAV) is a contagious species causing upper respiratory disease [1]. It is estimated that the occurrence of influenza results in approximately 3.5 million severe cases and 300,000 to 500,000 deaths annually [2]. Vaccination is commonly effective in healthy individuals but is often less effective in the elderly [3]. In addition, seasonal vaccines can be completely ineffective in the case of an antigenic mismatch between the virus present in the vaccine and the virus circulating in society; therefore, their utility in responding quickly to an influenza pandemic remains uncertain [4]. The 2009 H1N1 pandemic demonstrated how rapidly isolating and identifying the correct strain and producing sufficient vaccines worldwide is a very challenging task [1,5,6]. A variety of antivirals can be used to prevent influenza virus infection or to treat the disease on a long-term basis. They can also provide rapid deployment options during a pandemic [7]. The current FDA-approved standard treatments by the CDC (Centers for Disease Control and Prevention) are the neuraminidase (NA) inhibitors zanamivir, oseltamivir, peramivir, and the PA subunit inhibitor baloxavir [8] (Figure 1). However, current NA inhibitors are prescribed to uncomplicated patients with growing resistance within 48 h of infection [9]. Moreover, baloxavir is not indicated for patients under 12 years of age, and currently, strains resistant to baloxavir have developed [10]. The development of new anti-influenza drugs that have novel mechanisms of action, good therapeutic effects, low levels of drug resistance, and good safety is therefore critical.

The non-nucleoside polymerase inhibitor pimodivir (also known as JNJ-63623872, previously known as VX-787) inhibits the transcription progress by targeting the highly conserved site in the cap-binding site of the PB2 subunit of the influenza RNA-dependent RNA polymerase (RdRp) polymerase [11]. Based on an in vitro cell model with an EC_50_ ranging from 0.13 nM to 3.2 nM, VX-787 showed potent activity against several influenza A virus strains (emerged from 1933 to 2009) [12]. Moreover, VX-787 has no cytotoxicity when incubated in virus-free cells [12]. A phase I clinical trial showed no safety issues with the oral dose of VX-787 (600 mg, bid) in healthy volunteers [13], and a phase Ⅱ clinical trial reported that VX-787 could not only reduce the viral load in patients but also had no serious adverse effects [14]. Regrettably, however, its development was discontinued in September 2020 as its efficacy, confirmed in Phase Ⅲ clinical trials, was insufficient to provide benefits above the currently available standard of care [15].

**Figure 2:**
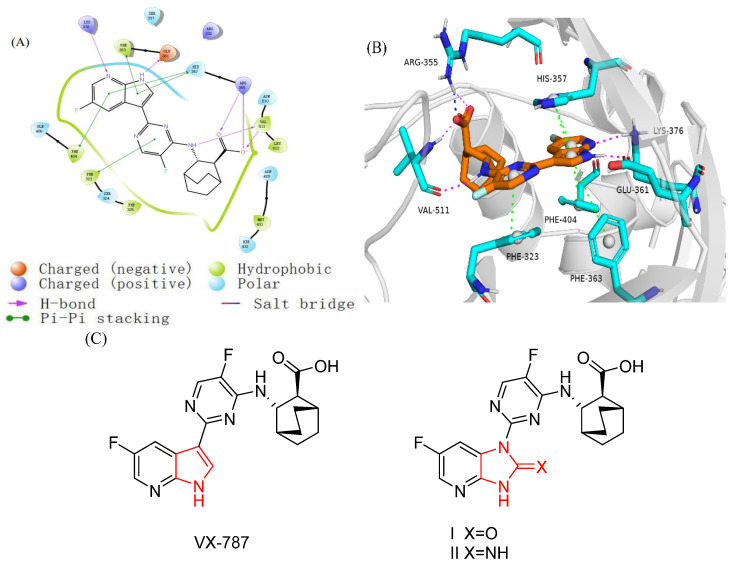
The 2D representation (**A**) and 3D summary (**B**) of the interaction analysis results of the binding mode of VX-787 to PB2, given by the crystal structure of the PB2/VX-787 protein complex (PDB 5WL0). The π–π stacking interaction is shown as a green dashed line, the intermolecular hydrogen bond as a magenta dashed line, and the salt bridge as a blue dashed line. (**C**) Structures of VX-787 and the designed comp. **I** and comp. **II**.

The binding mode of VX-787 and PB2 has previously been elucidated in detail [16]. As shown in Figure 2A,B, the X-ray crystal structure (PDB ID 5WL0) confirmed that VX-787 binds tightly to the cap-binding site of PB2. A significant role is played by the azaindole fragment in maintaining the binding of VX-787 to PB2. It forms hydrogen bonds between the protein residues and Lys376 and Glu361; stacks between the side chains of His357, Phe323, Phe363, and Phe404; and forms a typical sandwich structure with His357 and Phe404 [17]. Among three species (human, rat, mouse), VX-787 displayed the optimum liver microsome stability, although it was not optimally cleared from mouse plasma, suggesting there is another metabolic pathway besides CYP-mediated metabolism [13]. It has also been suggested that VX-787 analogs containing azaindole fragments metabolize at the C-2 position of azaindole in the presence of aldehyde oxidase (AO) to form 2-hydroxy-7-azaindole in vivo [18]. Accordingly, we speculated that VX-787 might also be an AO substrate. AO is a type of flavoprotein containing molybdenum (Mo), which requires the participation of flavin adenine dinucleotide and Mo pterin coenzyme for catalytic reactions [19,20]. It plays an important role in drug metabolism owing to its unique structure, distribution, and substrate selectivity [21,22]. Drugs that act as AO substrates typically exhibit high metabolic clearance rates, resulting in low exposure and, thus, have reduced efficacy in humans [23]. Recent studies have also shown that as a result of AO oxidation, reactive oxygen species and toxic metabolites can also be produced, which can cause liver injury and nephrotoxicity [16]. Therefore, blocking the labile site of the azaindole fragment of VX-787 so as to increase systemic exposure and reduce possible toxicity caused by AO metabolic processes are feasible optimization strategies for PB2 inhibitors. Based on this strategy, we replaced the azaindole fragment of VX-787 with 2,3-dihydro-imidazopyridines substituted with carbonyl or imine at the 2nd position (comp. I and comp. **II**, Figure 2C) in an attempt to block potential metabolic hotspots while maintaining nearly the same interactions with PB2 as that of the azaindole fragment in order to obtain PB2 inhibitors with good antiviral activities and high AO metabolic stabilities. 

## 2. Results

### 2.1. Molecular Docking 

**Figure 3:**
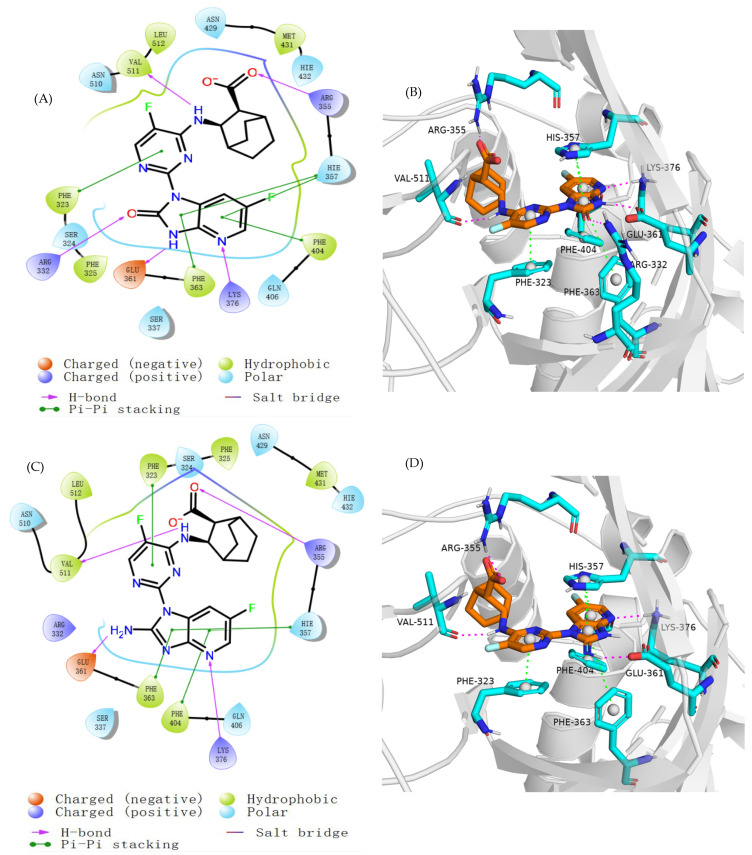
The 2D representation (**A**) and 3D summary (**B**) of the interaction analysis results of the PB2/comp. **I** protein complex. Proposed binding models of comp. **II** with PB2 protein shown as a 2D conformation (**C**) and 3D summary (**D**). The π–π stacking interaction is shown as a green dashed line, the intermolecular hydrogen bond as a magenta dashed line, and the salt bridge as a blue dashed line.

To examine the feasibility of our understanding, molecular docking of VX-787, comp. **I**, and comp. **II** with the cap-binding site of PB2 was performed using the Glide module of the Maestro version 10.7, (Schrodinger LLC, New York, NY, USA). The LigPrep calculation results showed that the 2-oxo-2,3-dihydro-imidazopyridine fragment of comp. **I** mainly exists in the form of 1,3-dihydro-2H-imidazo[4,5-b]pyridin-2-one, where the carbon-oxygen double bond is located outside the ring (Figure 3A,B), while the 2-2-imino-2,3-dihydro-imidazopyridine fragment of comp. **II** exists in the form of 1H-imidazo[4,5-b]pyridin-2-amine, where the carbon-nitrogen double bond is located inside the ring (Figure 3C,D). As mentioned above, Glide docking was performed with XP precision using the Glide grid for the generated receptor. The ligands were flexible with default parameters, and post-docking minimization was performed. The highest-ranked pose of the crystal structure (XP GScore of −11.519) was compared with the original pose of VX-787; the calculated root mean square deviation (RMSD) value was 0.2636 Å (Figure S1), suggesting that the docking process could accurately predict the binding pose of the true substrate.

### 2.2. Chemistry

**Scheme 1:**
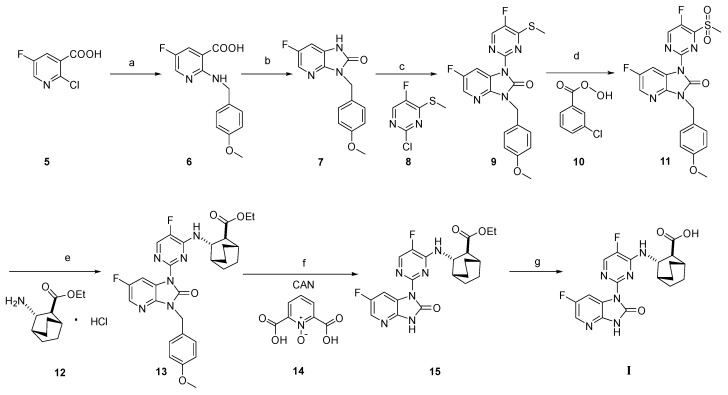
Synthesis route of comp. **I**. (a) p-methoxybenzylamine, NaHCO_3_, n-pentanol, 130 °C, 24 h, 51.8%; (b) TEA, DPPA, dry-tert-Butanol, 80 °C, reflux, 10 h, 76.3%; (c) Sodium bis(trimethylsilyl)amide, dry-THF, −30 °C, 12 h, 96%; (d) 3-chlorobenzoperoxoic acid, DCM, −10 °C, 4 h, 56%; (e) **12**, DIPEA, DMF, nitrogen atmosphere, 80 °C, 18 h, 37%; (f) cerium ammonium nitrate, CH_3_CN, H_2_O, rt, 18 h, 63%; (g) LiOH, THF, H_2_O, 50 °C, 7 h, 33%.

VX-787 was synthesized according to a previously published method [16]. As shown in Scheme 1, comp. **I** was synthesized using commercially available 2-chloro-5-fluoronicotinic acid. Nucleophilic substitution of 2-chloro-5-fluoronicotinic acid (**5**) with p-methoxybenzylamine resulted in compound **6** (51.8%) [24]. Compound **6** was allowed to react with diphenylphosphonium azide (DPPA) under basic conditions to form isocyanates via Curtius rearrangement, followed by intramolecular cyclization to yield 1,3-dihydro-2H-imidazo[4,5-b]pyridin-2-one (**7**) with 76.3% yield [25]. Compound **7** was coupled with compound **8** (prepared by nucleophilic substitution of 2,4-dichloro-5-fluoropyrimidine with sodium methanethiolate) [25] under basic conditions to obtain compound **9** with a yield of 96% [16]. Compound **9** was then oxidized using m-chloroperoxybenzoic acid (**10**) to yield sulfone compound **11** with 56% yield [26]. Compound **12** was prepared with reference to the literature method [27], and its configuration was determined to be (1R, 2S, 3S, 4R) (${\left[\alpha \right]}_{D}^{20}$== −17.4 (c, 1.0, MeOH); the literature value was${\left[\alpha \right]}_{D}^{20}$= −17.5 (c, 1.0, MeOH)). Its ee value was determined to be −98.75% by chiral chromatographic analysis (Figure S6-2)). In the presence of DIPEA, compound **12** underwent a substitution reaction with compound **11** to form compound **13** with 37% yield [28]. Deprotection of the p-methoxybenzyl (PMB) group of compound **13** with cerium ammonium nitrate (CAN) afforded a desired compound **15** with 63% yield [29]. Hydrolysis of the ester of compound **15** using lithium hydroxide in a mixture of THF and water afforded the desired target, comp. **I** [16]. In summary, the synthesis of comp. **I** was accomplished in seven steps under relatively mild conditions, with an overall yield of 1.63%.

**Scheme 2:**
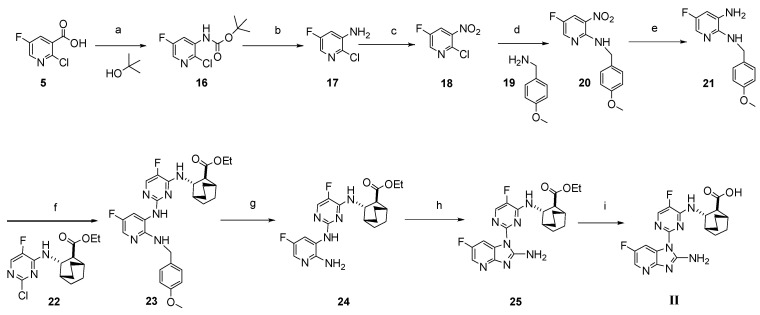
Synthesis route of comp. **II**. (a) DPPA, TEA, tert-butanol, nitrogen atmosphere, rt, 12 h, 45.4%; (b) TFA, DCM, 40 °C, 30 min, 99%; (c) H_2_O_2_, H_2_SO_4_, rt, 2 h, 59.7%; (d) 4-methoxybenzylamine, CsCO_3_, dioxane, 101 °C, 3 h, 77.3%; (e) ammonium formate, K_2_CO_3_; Fe, EtOH, H_2_O, 100 °C, 5 h, 79%; (f) **21**, (9,9-dimethyl-9H-xanthene-4,5-diyl)bis(diphenylphosphane), Cs_2_CO_3_, Pd(OAc)_2_, 140 °C, 30 min, microwave, 20%; (g) anisole, TFA, DCM, rt, 24 h, 41.3%; (h) bromoacetonitrile, MeOH, H_2_O, rt, 24 h, 10%; (i) LiOH, THF, H_2_O, 50 °C, 7 h, 45%.

The synthesis of comp. **II** was also initiated using 2-chloro-5-fluoronicotinic acid (**5**), as shown in Scheme 2. The compound 2-chloro-5-fluoronicotinic acid (**5**) was first subjected to Curtius rearrangement to form isocyanate and was then allowed to react with tert-butanol to form compound **16** (45.4%) [18]. The protective tert-butoxy group was removed under acidic conditions to obtain amino **17** with a 99% yield [30]. Oxidation of the amino group of compound **17** to the nitro group, using hydrogen peroxide in concentrated sulfuric acid, resulted in compound **18** [31]. The newly generated nitro group enhances the substitution activity of ortho-chlorine. Next, the chlorine in compound **18** was substituted with p-methoxybenzylamine to obtain compound **20** with a 77.3% yield [24]. The nitro group of **20** was reduced to an amino group using iron powder and ammonium formate to obtain **21** with a yield of 79% [32]. The ethyl(1R,2S,3S,4R)-3-((2-chloro-5-fluoropyrimidin-4-yl) amino) bicclo [2.2.2] octane-2-arboxylate (**22**) was prepared according to the literature [16]. Subsequently, compound **22** was coupled with compound **21** under the conventional microwave reaction conditions at 140 °C, using the catalyst (9,9-dimethyl-9H-xanthene-4,5-diyl)bis(diphenylphosphane) to generate compound **23** with **a** 20% yield [16]. The PMB-protecting group of the amino group was removed under acidic conditions (TFA) to generate compound **24** with a yield of 41.3% [33]. Compound **24** was then cyclized in the presence of bromoacetonitrile to afford 1H-imidazo[4,5-b]pyridin-2-amine (**25**) [34]. Finally, hydrolysis of the ester with lithium hydroxide in THF-H_2_O (1:1) at 50 °C, gave the desired comp. **II** with 45% yield [16]. In summary, the synthesis of comp. **II** was accomplished in nine steps under relatively mild conditions with an overall yield of 0.15%.

### 2.3. Biochemical Assays

#### 2.3.1. SPR Competitive Binding Assay

Biomolecular interaction analysis based on surface plasmon resonance (SPR) assays was performed using VX-787 and comp. **I** and **II** at concentrations ranging from 20 to 0.156 µM to investigate their competitive binding affinities with PB2 (Table 1).

#### 2.3.2. Cytopathic Effect (CPE) Inhibition Assay and Cytotoxicity Assay

The antiviral activities of comp. **I** and comp. **II** against influenza virus A/Puerto Rico/8/1934 (H1N1) and A/Hong Kong/8/68 (H3N2) were tested in MDCK cells by a cytopathic effect (CPE) inhibition assay. Oseltamivir and VX-787 were used as a positive control. Comp. **I** and **II** were found to rescue cells from CPE induced by influenza viruses at non-cytotoxic concentrations (Table 1).

#### 2.3.3. Metabolic Stability

**Figure 4:**
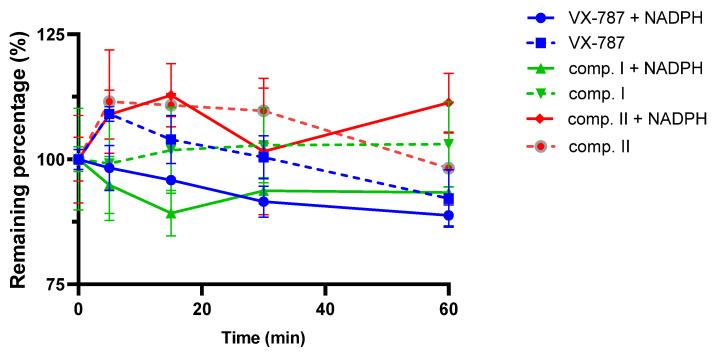
Residual percentage of VX-787 and comp. **I**/**II** in rat liver microsomes. Error bars are represented as mean ± SD, *n* = 3.

**Figure 5:**
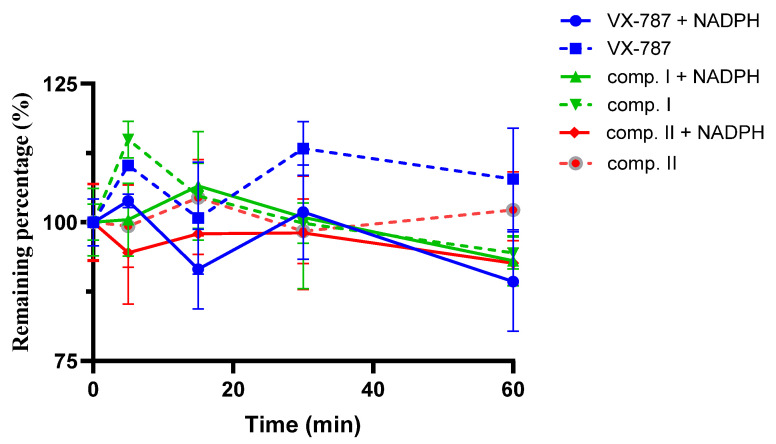
Residual percentage of VX-787 and comp. **I**/**II** in human liver microsomes. Error bars are represented as mean ± SD, *n* = 3.

In vitro metabolic stability of comp. **I** and comp. **II** were tested in rats (Figure 4 and Table S1) and human liver microsomes (Figure 5 and Table S2), using VX-787 as a positive control. The control without NADPH was also used to reveal any NADPH-independent enzymatic degradation or chemical instability. VX-787, comp. **I**, and comp. **II** underwent degradation in the presence of NADPH but remained intact in the absence of NADPH, indicating that NADPH-dependent enzymes (CYP450 enzymes) are responsible for their degradation.

**Figure 6:**
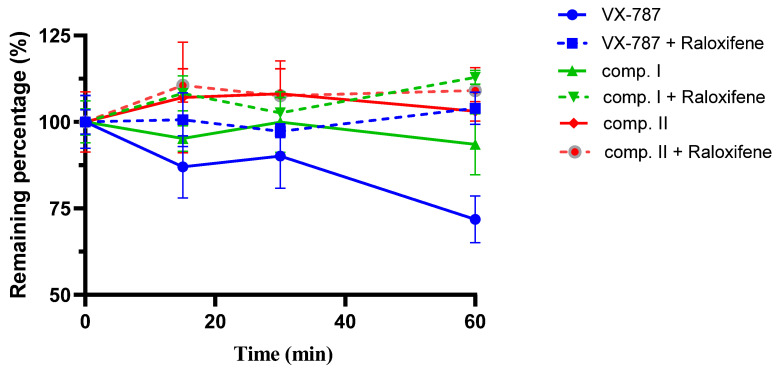
Residual percentage of VX-787 and comp. **I**/**II** in 0.5 mg/mL human liver cytoplasm. Error bars are represented as mean ± SD, *n* = 3.

Human liver cytosolic protein was incubated with VX-787, comp. **I**, and comp. **II** for 60 min at 37 °C, with and without the AO inhibitor raloxifene, to determine whether AO-mediated metabolism was modulated. Table S3 and Figure 6 summarize the results.

Regarding metabolic stability, comp. **I** and comp. **II** showed better liver microsome stability in both species (human/rat) and better human cytoplasmic stability than VX-787 (Table 2).

These results showed that the conversion of azaindole to 2-substituted 2,3-dihydro-imidazoline significantly improved the metabolic stability of comp. **I** and comp. **II**, compared to that of VX-787. These results also suggested that VX-787 is indeed an AO substrate and that the conversion of azaindoles to 2-substituted 2,3-dihydro-imidazopyridine can reduce substrate sensitivity to AO. Comp. **II** was nearly unaffected by AO aldehyde oxidase, whereas comp. **I** remains as a substrate for AO-mediated metabolism, thus suggesting that other AO metabolic sites still exist for comp. **I**, and should be further investigated in future optimization studies.

### 2.4. Molecular Dynamics (MD) Simulation

**Figure 7:**
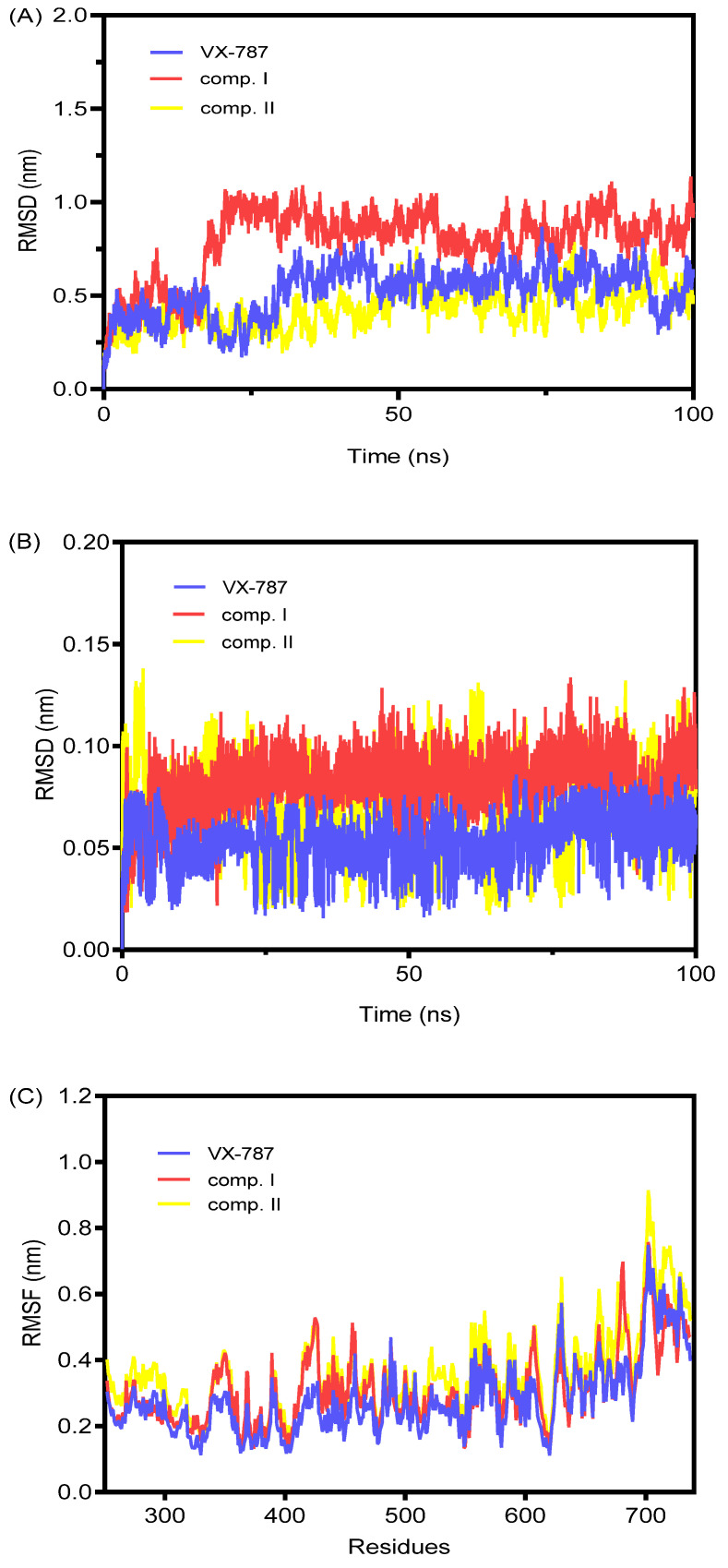

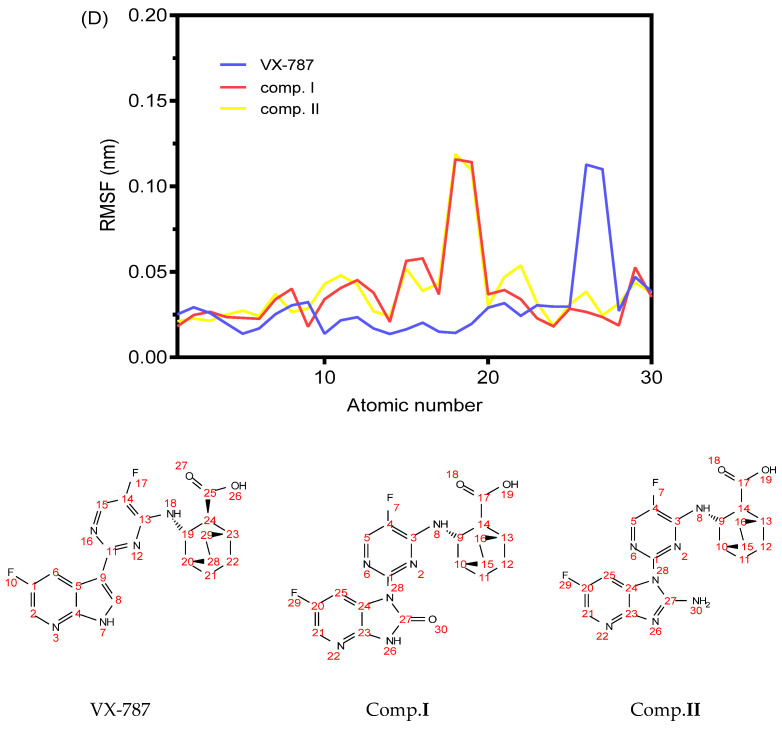
Stabilities of VX-787, comp. **I**, and comp. **II** in PB2 protein during 100 ns molecular dynamics (MD) simulations. (**A**) RMSD values of PB2 protein backbone of the PB2/ligand complexes. (**B**) RMSD of Lig fit Prot values for VX-787, comp. **I** and comp. **II**. (**C**) RMSF values of amino acids of PB2/ligand complexes. (**D**) The RMSF values of atomic positions of VX-787, Comp. **I** and Comp. **II**.

To analyze the interactions between influenza PB2 and VX-787, comp. **I**, comp. **II**, the structural complexes of influenza PB2 docked with VX-787, comp. **I**, and comp. **II** were assessed by MD simulation using GROMACS software (2021.3) [35]. The PB2/ligand complex models were placed in the orthorhombic box at a buffer distance of 10 angstroms, and a hydration model was established using the TIP4P water model. Each simulation system was neutralized by adding an appropriate number of ions (Cl^-^). For each system, the energy was minimized by using the steepest descent algorithm, followed by 1000 ps ensemble equilibrations with NVT and NPT (at 300 K and 1 bar). For each of the equilibrated systems, a production simulation of 100 ns was carried out due to the trade-off between simulation accuracy and computing power. Based on the trajectory data of the production simulations, root mean square deviations (RMSDs) and root mean square fluctuations (RMSFs) were calculated (Figure 7).

To estimate the free binding energies between VX-787, comp. **I**, comp. **II** and PB2, molecular mechanics/Poisson–Boltzmann surface area (MM/PBSA) analyses were performed. MM/PBSA is a common method used to determine the free binding energy between a protein and a ligand. In this method, the polar energy term is approximated using the Poisson–Boltzmann (PB) equation, the nonpolar energy term is approximated by the solvent-accessible surface area (SASA) equation, and the entropy term uses normal mode analysis. Binding energies can be calculated reliably using this method [36].

Essentially, the MM/PBSA method evaluates the free binding energy between a protein and ligand from MD simulation snapshots using the energy terms of the MD force field. For single snapshots of the MD trajectory, the binding-free energy of the ligand to the protein (∆G_bind_) was calculated from MD simulations of the protein complex as the average difference between the free energies of the complex state (∆G_comp_) and the unbound states of the protein (∆G_rec_) and the ligand (∆G_lig_):

∆G_bind_ = ∆G_comp_ − (∆G_rec_ + ∆G_lig_) (1)

The ∆G_Bind_ of protein–ligand complex can be evaluated using the following approximation based on the second law of thermodynamics:

∆G_bind_ =∆H − T∆S ≈ ∆Egas + ∆Gsol − T∆S ≈ ∆E_gas_ +∆G_sol_ (2)

For non-covalently bound protein–ligand complex systems, the ∆E_gas_ component is the vacuum interaction energy derived from non-bonded interactions and consists of van der Waals energy (∆E_vdw_) and electrostatic energy (∆E_ele_) contributions.

∆E_gas_ = ∆E_vdw_ + ∆E_ele_ (3)

The ∆G_solv_ component is the solvated free energy, composed of polar (∆E_pb_) and nonpolar (∆E_surf_) solvation components.

∆G_solv_ = ∆E_pb_ + ∆E_surf_ (4)

**Figure 8:**
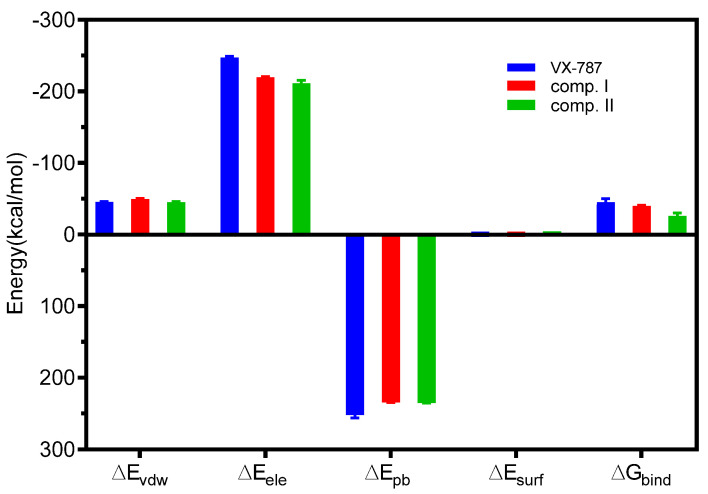
Calculated energy contribution decomposition values of binding−free energies by the MMPBSA method (All in kcal/mol).

In order to perform the MM/PBSA analysis, we extracted 1000 snapshots from the last 10 ns MD simulationsusing thegmx_MM/PBSA tool(Gromacs) [37], where all the simulated systems reached equilibrium [38]. The results are shown in Figure 8 and Table S4. A correlation can be found between the calculated ΔG_bind_ and the equilibrium dissociation constant (K_D_) determined by the SPR method (Table 1).

**Figure 9:**
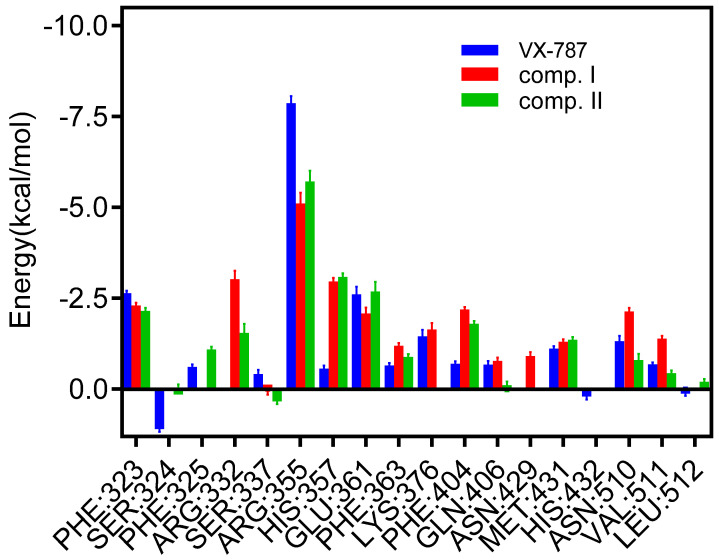
Per−residue binding energy decomposition of VX-787, comp. **I**, and comp. **II**.

The results of the per-residue MM/PBSA energy decomposition analysis for residues within the distance of 4 Å to the ligand are shown in Figure 9 and Table S5. The results showed that the residues Phe323, Arg355, and Glu361 play key roles in maintaining the binding between VX-787, comp. **I**, comp. **II**, and PB2 protein. The residues His357, Phe363, Phe404, Met431, Asn510, and Val511 are important for maintaining the binding between the ligand and the PB2 protein.

## 3. Discussion

### 3.1. Molecular Docking Analysis

Docking results showed that the binding mode of comp. **I** and comp. **II** with PB2 were similar to those of VX-787 and PB2 (Figure 3). The XP GScore values of the highest-ranked pose of comp. **I** and comp. **II** were −10.550 and −10.475, respectively, which is very close to that of VX-787. The RMSDs of these two poses, from the original pose of VX-787, were 0.6617 and 2.3213 Å, respectively (Figure S2). Comp. **I** and comp. **II** bound to the cap-binding pocket of PB2, and their flat fused-ring structures perfectly matched the shape features of the binding pocket. The bicyclo[2.2.2]octane-2-carboxylic acid fragment and 5-fluoropyrimidin-4-amine fragment of comp. **I** and comp. **II**, similar to that of VX-787, retained hydrogen bonds, ionic bridges, hydrophobicities, and π–π stacking with PB2. In addition, the 2-substituted 2,3-dihydro-imidazopyridine fragments of comp. **I** and comp. **II** retained nearly the same interactions with PB2 as that of the azaindole ring of VX-787, forming hydrogen bonds with the protein residues Lys376 and Glu361, and interacting with side chains His357, Phe232, Phe363, and Phe404 via π–π stacking interactions. A slight difference is that the carbonyl group of the 1,3-dihydro-2H-imidiazo[4,5-b]pyridin-2-one fragment of comp. **I** can also form additional hydrogen bonds with Arg332, and 1H-imidazo[4,5-b]pyridin-2-amine of comp. **II** forms a hydrogen bond with Glu361 via a proton on the exocyclic amino group rather than via an intracyclic proton, as in the case with azaindole of VX-787. Based on the above analysis, although the docking scores of comp. **I** and comp. **II** are slightly lower than those of VX-787, we believe that it is still meaningful to synthesize them and investigate their anti-influenza activities.

### 3.2. Biochemical Assay Analysis

#### 3.2.1. SPR Competitive Binding Assay Analysis

The results showed that compounds **I** and **II** bound to the PB2 protein with K_D_ values of 1.398 μM and 1.670 μM, respectively, compared to the K_D_ value of 0.152 μM for VX-787. This indicated that both comp. **I** and **II** have good affinities for PB2 but are not as strong as VX-787.

#### 3.2.2. CPE Inhibition Assay and Cytotoxicity Assay Analysis

Comp. **I** inhibited influenza A/Puerto Rico/8/1934 (H1N1) and A/Hong Kong/8/68 (H3N2) in a dose-dependent manner with IC_50_ values of 0.07 ± 0.02 μM and 0.04 ± 0.01 μM, respectively. The IC_50_ values of comp. **II** against influenza A/Puerto Rico/8/1934 (H1N1) and A/Hong Kong/8/68 (H3N2) viruses were 0.09 ± 0.05 μM and 0.07 ± 0.03 μM, respectively (Table 1). Neither compound significantly differed in its antiviral activity against H1N1 or H3N2 viruses. Although comp. **I** and comp. **II** were not as active against H1N1 and H3N2 as VX-787, they were significantly more active than oseltamivir against H1N1 and nearly similar to oseltamivir against H3N2. In addition, comp. **I** and **II**, similar to VX-787 and oseltamivir, had CC_50_ values greater than 200 μM, thus indicating low cytotoxicity.

As a result of both analyses, the CPE inhibition and SPR competitive binding results were consistent: the stronger the binding of the compound to PB2, the higher its inhibitory activity against influenza viruses H1N1 and H3N2. This provided sufficient evidence to prove that the inhibitory activity of comp. **I** and **II** against the influenza virus could be achieved by inhibiting the activity of PB2.

#### 3.2.3. Metabolic Stability Analysis

In the presence of NADPH, both comp. **I** and **II** showed a decrease in the remaining percentage after incubation in rat or human liver microsomes, but the decrease was not as pronounced as that for VX-787. At 60 min, the concentrations of comp. **II** slightly changed in the rat liver microsomes, the concentration of comp. **I** was 93% of the initial concentration, and that of VX-787 was only 88% of the initial concentration with NADPH (Table S1). In human liver microsomes, the percentages of comp. **I** (93.07%) and comp. **II** (92.65%) were higher than that of VX-787 (89.33%) at 60 min with NADPH (Table S2).

All tested compounds were stable in the human liver cytosol in the presence of raloxifene. Comp. **I** and comp. **II** appeared to be more stable than VX-787 without raloxifene in the cytosol. At 60 min, the concentration of comp. **II** barely changed, and the concentration of comp. **I** was 93% of the initial concentration, whereas the concentration of VX-787 was only 72% of the initial concentration (Table S3). VX-787 suffered from short T_1/2_, and its half-lives in the liver microsomes of humans and rats were 349.29 min and 249.46 min, respectively. Moreover, its half-life in human cytoplasm was 150 min. However, both comp. **I** and comp. **II** showed longer T_1/2_ (all greater than 800 min) in human and rat liver microsomes and human cytoplasm. Consequently, the clearances of comp. **I** (0.53 mL/min/kg in rats, <0.1 mL/min/kg in humans) and comp. **II** (<0.1 mL/min/kg in rat, 0.46 mL/min/kg in human) in microsomes were significantly lower when compared to that of VX-787 (1.72 mL/min/kg in rat, 1.79 mL/min/kg in human).

### 3.3. MD Simulation Analysis

The variation trends of the RMSD values of proteins and ligands are an important basis for understanding the simulation stability of protein–ligand complexes. A plot of the RMSD values of the heavy atoms of the PB2 backbone, over time, for each assay system, is shown in Figure 7A. All three systems reached equilibrium after 40 ns of simulation, and the RMSD values of the PB2 protein backbone fluctuated around 0.2 nm and 1.2 nm. The results indicated that, in all three PB2–ligand complex systems, the RMSD of the Cα atom of the PB2 protein was stable during the simulation. The RMSD of the Lig fit profile of ligands in the MD simulations is presented in Figure 7B. There was only a small fluctuation of the RMSD value of the ligands (from 0.02 to 0.13 nm), which indicated that the interaction of VX-787, comp. **I**, comp. **II**, and the protein were always stable during the 100 ns simulation, and the ligands were always bound to the binding pocket.

The RMSF for the residues was the time-averaged fluctuation of the square deviation of a designated set of residue atoms over the entire simulation time. It provides information on the fluctuations in each amino acid residue in the protein, and large fluctuations in amino acids imply great flexibility in the complex. The RMSFs of the PB2 amino acid residues in each MD simulation are shown in Figure 7C. Amino acid residues farther from the cap-binding site (AA > 530) in the PB2 protein had higher RMSF than amino acid residues closer to the cap-binding site (AA < 530). PB2 binding to ligands reduces the flexibility of amino acid residues adjacent to binding sites, suggesting that PB2 binds them firmly. For the amino acids close to the binding site, their RMSF values when bound to VX-787 were lower than their RMSF values when bound to comp. **I** and **II**, which revealed that the PB2/VX-787 complex is more stable than comp. **I** or **II** bound to PB2. This is consistent with our test results, where comp. **I** and **II** showed less affinity for PB2 than VX-787, as demonstrated by the SPR assay. Overall, the amino acid residues adjacent to the cap-binding site (AA < 530) with large fluctuations were concentrated in only a few fragments. The RMSF values of most of the amino acids adjacent to the cap-binding site were less than 4.0 Å, which further proved that these PB2–ligand complexes were stable during the simulation. The RMSF values of atomic positions of VX-787, Comp. **I**, and Comp. **II** have been presented in Figure 7D. It can be observed that the RMSF profiles of the three investigated compounds display similar characteristics. Except for carboxyl oxygen atoms (No.18 and No.19 atoms of Comp. **I** and Comp. **II** and No. 26 and No. 27 atoms of VX-787), other atoms fluctuate slightly. These results showed that the conformations of VX-787, Comp. **I**, and Comp. **II** all remained close to their initial structure.

We also conducted a trajectory clustering analysis to estimate the most populated representative structure in each MD simulation. The structure with the most neighbors in the structural cluster was selected as the representative structure for each complex (Figure S3). Comparing the representative conformations of the three systems with the initial conformations of the respective MD simulations (i.e., the docking conformations of PB2 with VX-787, Comp. **I**, and Comp. **II**), it can be found that the representative conformations are similar to their corresponding docking conformations ((Figure 2, Figure 3 and Figure S3). This further indicated that the conformation of the complexes of VX-787, Comp. **I**, Comp. **II** and PB2 did not undergo significant changes, and that the binding mode of VX-787, Comp. **I**, Comp. **II**, and PB2 remained basically unchanged during the MD simulation (Figure S3).

As shown in Figure 8 and Table S4, the lower the value of the K_D_, the lower the ΔG_bind_ between the protein and ligand. This indicates that the free binding energy assessed using the MM/PBSA method can accurately predict the affinity of the PB2 protein to the ligands. Subsequently, we also found that the correlation between the calculated ΔG_bind_ and the K_D_ values was not particularly good. This may be due to the following reasons: First, MM/PBSA is a method used for the approximate calculation of binding-free energy, which ignores the contribution of entropy during calculation, and its accuracy in some systems is on the order of 10 kJ/mol (approximately 2.4 kcal/mol). Second, the MM/PBSA method was used to measure the thermodynamic stability of the model without considering kinetic stability, such as the energy barrier that occurs when the ligand binds to PB2, while the K_D_ measures a kinetic process. In addition, owing to the high computational cost and low prediction accuracy, there was a large deviation between the results obtained by our dynamic simulation and those obtained in the real world. For all three PB2–ligand complex systems, the electrostatic energy (ΔE_ele_) had large negative values, whereas ΔE_ele_ was reversed by the less favorable polar solvent-free energy (∆E_pb_, Figure 8 and Table S4). The absolute values of the sum of ΔE_ele_ and ∆E_pb_ for all three systems were positive. This indicates that ligand binding to the PB2 cap-binding site is not driven mainly by electrostatic force (polar) [39]. The values for the nonpolar solvation (ΔE_surf_) fraction of the three systems were small (∆E_pb_, Figure 8 and Table S4) while the ΔE_vdw_ fraction of the three systems was significantly larger; thus, it can be concluded that ligand binding to PB2 is mainly driven by van der Waals interactions. In our employed MM/PBSA model, the values of the ΔE_vdw_ fraction and the nonpolar solvation (ΔE_surf_) fraction of the three systems were not significantly different. The ΔE_ele_ value of the VX-787/PB2 system was significantly more negative than that of the other two systems (the absolute value difference is 27.06 kcal/mol and 35.62 kcal/mol, respectively). Although ∆E_pb_ significantly offsets this difference, the absolute value of the sum of ΔE_ele_ and ∆E_pb_ for the VX-787/PB2 system (+4.59 kcal/mol) was significantly smaller than the absolute value of the sum of ΔE_ele_ and ∆E_pb_ for the PB2/comp. **I** (+13.95 kcal/mol) and PB2/comp. **II** (+23.39 kcal/mol) systems. The results suggested that, although VX-787, comp. **I**, and comp. **II** bound to PB2 are primarily more driven by van der Waals interactions, the difference in electrostatic (polar) interactions is the main reason for VX-787 having a better affinity for PB2 than comp. **I** and **II**.

Per-residue MM/PBSA energy decomposition analysis results are shown in Figure 9 and Table S5. These results are consistent with the binding mode and the structure–activity relationship information of VX-787 provided in the literature [40]. These results also showed that the binding modes of VX-787, comp. **I**, comp. **II**, and PB2 are essentially the same, which is in line with our expectations. Based on the per-residue MM/PBSA energy decomposition analysis, it appears that VX-787, comp. **I**, and comp. **II** have slightly different bindings to PB2. (1) Both Lys376 and Gln406 contributed significantly to VX-787 and comp. **I** binding to PB2, but had little effect on the binding of comp. **II** to PB2. (2) Residues His357 and Phe404 contributed significantly more to the binding of comp. **I** and comp. **II** to PB2 than VX-787 to PB2. (3) Arg332 contributed more to the binding of comp. **I** and comp. **II** to PB2, but not to the binding of VX-787 to PB2. These five residues—Lys376, Phe404, Gln406, His357, and Arg332—have direct contact with the azaindole fragment of VX-787 or the 2-substituted 2,3-dihydro-imidazopyridine fragments of comp. **I** and **II**. Therefore, although comp. **I** and **II** can maintain similar interactions to PB2 as VX-787, the structural change from azaindoles to 2-substituted 2,3-dihydro-imidazopyridine has a substantial impact on how these two compounds bind to PB2. Unexpectedly, the structural change of the azaindoles to 2-substituted 2,3-dihydro-imidazopyridine also affected the contribution of Arg355 to the binding-free energy. As compared to PB2/comp. **I** or PB2/comp. **II**, Arg355 contributed a much higher amount to the binding-free energy of PB2/VX-787, as shown in Figure 9. These results indicated that VX-787 has good structural adaptability to PB2. Although comp. **I** and comp. **II** maintained similar interactions with PB2 as VX-787, comp. **I** and comp. **II** have lower structural adaptability to PB2 than VX-787 to PB2, which suggests that the affinities of comp. **I** and **II** for PB2 are lower than those of VX-787 for PB2. This difference in the structural adaptability of comp. **I** and **II** with VX-787 are mainly due to the difference in the electrostatic (polar) distribution.

Our molecular dynamics simulation and MM/PBSA calculation results show that: (1) the bindings of ligands to PB2 are primarily driven by van der Waals interactions, (2) the difference in the electrostatic (polar) distribution is the main reason for VX-787 to have a better affinity for PB2 than Comp. **I** and Comp. **II**. This suggests that, when designing PB2 inhibitors, special attention should be paid to the adaptability of the overall structure of the inhibitor molecule to the structure of PB2 protein, particularly the adaptability of the electrostatic (polar) interaction of the inhibitor. At the same time, the polar surface area of the molecule should be minimized to maximize van der Waals interactions with the PB2 protein.

## 4. Materials and Methods

### 4.1. Chemistry

#### General Procedures

All reagents’ solvents and starting materials were obtained from Bide Pharmatech Ltd. (Beijing, China), Beijing Inokai Technology Co., Ltd. (Beijing, China), Beijing Anai Jisesheng Technology Co., Ltd. (Beijing, China), Beijing Yihe Technology Co., Ltd. (Beijing, China), and Sinopec Chemical Reagent Co., Ltd. (Beijing, China). No further purification was required. Purity assessment for final compounds was based on analytical HPLC: 150 × 4.6 mm Agilent Technologies 1260 infinity Diamonsil C18 column, 5 μm. Mobile phases are as follows: A, H_2_O with 0.5% formic acid; B, acetonitrile; gradient, 70% A and 30% B over 20 min at a flow rate of 1 mL/min. Optical purity assessment for benzoylated compound 12 was based on analytical HPLC: 0.46 cm × 15 cm Agilent Technologies 1260 infinity Daicel Chiralcel ODH column, 5 μm. Mobile phases are as follows: 95% Hexane (with 0.5% HCOOH) and 5% IPA (0.1%DEA) over 15 min at a flow rate of 1.0 mL/min. The crude reaction mixtures were concentrated under reduced pressure by removing the organic solvents in a rotary evaporator. Reactions were monitored by thin layer chromatography (TLC) using Kieselgel 60 F254 (E. Merck) plates and a UV detector for visualization. Flash column chromatography was performed with a Biotage medium and high-pressure integrated purification separator. All reported yields are of purified products. Mass spectra were recorded on an API3000LC/MS spectrometer. NMR spectra were recorded at 25 °C on a JNM-ECA-400 superconducting NMR instrument at 400 MHz or a Bruker Avance 600 (600 MHz) instrument for ^1^ H and 101 MHz for ^13^C and 376 MHz for ^19^F.

5-fluoro-2-((4-methoxybenzyl)amino)nicotinic acid (**6**)

NaHCO_3_ (1260 mg, 15 mmol) was dissolved in 10 mL n-amyl alcohol, and 2-chloro-5-fluoronicotinic acid (**5**) (877.7 mg, 5 mmol) were added to the reaction system while stirring. Then, p-methoxylbenzylamine (1.3 mL, 10 mmol) was added to the reaction system drop by drop, and nitrogen was added to protect the mixture. The reaction was stirred at 130 °C for 12 h until TLC detection of the reaction was complete, and the heating was stopped at the end of the reaction. After cooling to room temperature, the mixture of 2.5 mL water and 2.5 mL methanol was added and stirred at room temperature for 1 h. The pH of the solution was 5, and yellow-green solids were precipitated, filtered, washed with 8 mL water, and washed with 10 mL diethyl ether. Synthesized as a white solid in 51.8% yield. ^1^H NMR (400 MHz, DMSO-*d*_6_) δ 8.31 (d, *J* = 3.1 Hz, 1H), 7.92 (dd, *J* = 8.9, 3.1 Hz, 1H), 7.25 (d, *J* = 8.6 Hz, 2H), 6.88 (d, *J* = 8.7 Hz, 2H), 4.56 (s, 2H), 3.72 (s, 3H). ESI-MS (*m*/*z*) calculated for C_14_H_14_FN_2_O_3_ 277.09, found 277.10 [M+H]^+^.

6-fluoro-3-(4-methoxybenzyl)-1,3-dihydro-2H-imidazo[4,5-b]pyridin-2-one (**7**)

The reactant **6** (616 mg, 2.2 mmol) was added droplet by droplet to anhydrous tert-butanol (10 mL). Next, 1eq TEA was added, and then DPPA (665 mg,2.42 mmol) was added droplet by droplet under nitrogen protection. Then, the temperature was raised to 80 °C for reflux overnight for approximately 10 h until the reaction was completely monitored by TLC. After cooling to room temperature, the solid was precipitated and filtered. There were no product points in the filtrate and a small number of impurities in the solid. After washing with DCM, a yellow solid was obtained 45.8 mg with a yield of 76.32%. m.p. 167–168 °C .^1^H NMR (400 MHz, DMSO-*d*_6_) δ 11.42 (s, 1H), 7.93 (t, *J* = 2.4 Hz, 1H), 7.35 (dd, *J* = 8.8, 2.5 Hz, 1H), 7.30–7.22 (m, 2H), 6.90–6.82 (m, 2H), 4.92 (s, 2H), 3.70 (s, 3H). ESI-MS (*m*/*z*) calculated for C_14_H_13_FN_3_O_2_ 274.09, found 274.10 [M+H]^+^, 296.08 [M+Na]^+^.

2-chloro-5-fluoro-4-(methylthio)pyrimidine (**8**)

2,4-dichloro-5-fluoropyrimidine (3 g,18 mmol) was dissolved in 30 mL THF cooled to −30 °C, and sodium methanethiolate (1.35 g,19.3 mmol) was added at a low temperature for 12 h until TLC detection of the reaction was complete. After the reaction was restored to room temperature, 30 mL saturated sodium chloride solution was added to the reaction solution and extracted with ethyl acetate (3 × 10 mL); the organic layer was dried and concentrated. An amount of 2.4 g was synthesized as a light orange solid by reduced pressure distillation with a yield of 96%. ^1^H NMR (400 MHz, Chloroform-*d*) δ 8.07 (s, 1H), 2.59 (s, 3H). ESI-MS (*m*/*z*) calculated for C_5_H_4_ClFN_2_S 177.98.

6-fluoro-1-(5-fluoro-4-(methylthio)pyrimidin-2-yl)-3-(4-methoxybenzyl)-1,3-dihydro-2H-imidazo[4,5-b]pyridin-2-one (**9**)

Compound **7** (0.53 g, 2 mmol) was dissolved in double (trimethylsilyl alkyl) sodium amino (2 mL, 2 M), 2 mL anhydrous THF was added for ultrasonic degasification for 3 min, and then argon was passed under the page by glass drip irrigation for 5 min. Reactant **8** (1.78 g, 10 mmol) was added for microwave reaction and set to 130 °C for 10 min. At the end of the reaction, it was cooled to room temperature, and the residue was purified by silica gel chromatography (petroleum ether/ethyl acetate, 5:1) to give the compound **9** (796.8 mg, 96%) as a light blue liquid. ^1^H NMR (400 MHz, Chloroform-*d*) δ 8.29 (d, *J* = 1.4 Hz, 1H), 8.05–8.01 (m, 1H), 7.98 (dd, *J* = 8.8, 2.6 Hz, 1H), 7.52 (d, *J* = 8.7 Hz, 2H), 6.83 (d, *J* = 8.7 Hz, 2H), 5.14 (s, 2H), 3.76 (s, 3H), 2.71 (s, 3H). ESI-MS (*m*/*z*) calculated for C_19_H_16_F_2_N_5_O_2_S 416.09, found 416.09 [M+H]^+^, 438.07 [M+Na]^+^.

6-fluoro-1-(5-fluoro-4-(methylsulfonyl)pyrimidin-2-yl)-3-(4-methoxybenzyl)-1,3-dihydro-2H-imidazo[4,5-b]pyridin-2-one (**11**)

Compound **9** (880 mg, 2.118 mmol) was dissolved in 70 mL dichloromethane, stirred and cooled to −11 °C. The 3-chlorobenzoperoxoic acid (**10**) (50%) 1.05eq was dissolved in the reaction system, and the reaction began. The temperature was kept below −10 °C for 4 h. After the reaction, 5 mL 10% NaHSO_3_ was added to quench the reaction and stirred for approximately 30 min. The reaction solution was washed with saturated sodium bicarbonate and saturated salt water, respectively, and the organic phase was dried with anhydrous sodium sulfate. Synthesized as a light-yellow solid in 56% yield. ^1^H NMR (400 MHz, DMSO-*d*_6_) δ 9.42 (d, *J* = 1.7 Hz, 1H), 8.23–8.21 (m, 1H), 8.19 (d, *J* = 9.1 Hz, 1H), 7.35 (d, *J* = 8.7 Hz, 2H), 6.89 (d, *J* = 8.8 Hz, 2H), 5.06 (s, 2H), 3.71 (s, 3H), 3.55 (s, 3H). ESI-MS (*m*/*z*) calculated for C_19_H_16_F_2_N_5_O_4_S 448.08, found 448.04 [M+H]^+^, 470.02 [M+Na]^+^.

(1R,2S,3S,4R)-Ethyl 3-aminobicyclo[2.2.2]octane-2-carboxylate hydrochloride (**12**)

Compound **12** was prepared with reference to the literature method [27], and its configuration was determined to be (1R, 2S, 3S, 4R) (${\left[\alpha \right]}_{D}^{20}$= –17.4 (c, 1.0, MeOH), the literature value was${\left[\alpha \right]}_{D}^{20}$= –17.5 (c, 1.0, MeOH)), and its ee value was determined to be −98.75% by chiral chromatographic analysis. ^1^H NMR (600 MHz, DMSO-*d*_6_) δ 8.21 (s, 3H), 4.13 (m, 2H), 3.59 (s, 1H), 2.55 (d, *J* = 5.8 Hz, 1H), 1.91(q, J = 2.9 Hz, 1H), 1.81 (m, 2H), 1.69 (m, 1H), 1.51 (m, 3H), 1.38 (q, *J* = 13.9, 13.4 Hz, 2H), 1.29 (m,1H), 1.21 (t, *J* = 7.1 Hz, 3H). ESI-MS (*m*/*z*) calculated for C_11_H_19_NO_2_ 198.27, found 198.15 [M+H]^+^.

ethyl(1R,2S,3S,4R)-3-((5-fluoro-2-(6-fluoro-3-(4-methoxybenzyl)-2-oxo-2,3-dihydro-1H-imidazo[4,5-b]pyridin-1-yl)pyrimidin-4-yl)amino)bicyclo[2.2.2]octane-2-carboxylate (**13**)

Compound **11** (700 mg,1.57 mmol) was dissolved in 30 mL DMF and compound **12** (1.2 eq) was added while stirring. Then, DIPEA (2.2 eq) was added drop by drop under the condition of nitrogen protection. After that, the internal temperature of the system was controlled at 80 °C for 18 h until the reaction was complete. Then, the organic phase was washed with saturated salt water and dried with anhydrous sodium sulfate. Column chromatography of petroleum ether:ethyl acetate 3:1–2:1 produced the product point, and was synthesized as a yellow solid in 37% yield. ^1^H NMR (400 MHz, DMSO-*d*_6_) δ 8.26 (d, *J* = 3.3 Hz, 1H), 8.12 (d, *J* = 1.8 Hz, 1H), 8.01–7.96 (m, 1H), 7.95 (d, *J* = 3.8 Hz, 1H), 7.32 (d, *J* = 8.6 Hz, 2H), 6.88 (d, *J* = 8.7 Hz, 2H), 5.01 (s, 2H), 4.07–3.96 (m, 2H), 3.70 (d, *J* = 2.7 Hz, 3H), 2.89 (s, 2H), 2.73 (s, 2H), 1.95 (s, 1H), 1.87 (s, 1H), 1.73 (m, 1H), 1.51 (m, 2H), 1.37 (m, 3H),1.11 (t, *J* = 7.1 Hz, 3H). ESI-MS (*m*/*z*) calculated for C_29_H_31_F_2_N_6_O_4_ 565.23, found 565.24 [M+H]^+^.

(1R,2S,3S,4R)-3-((5-fluoro-2-(6-fluoro-2-oxo-2,3-dihydro-1H-imidazo[4,5-b]pyridin-1-yl)pyrimidin-4-yl)amino)bicyclo[2.2.2]octane-2-carboxylic acid (**15**)

Compound **13** (112.8 mg, 0.2 mol) was dissolved in 2 mL acetonitrile, and 6eq 1-(l1-oxidaneyl)-1l4-pyridine-2,6-dicarboxylic (**14**) was added while stirring after stirring evenly, 3 eq ammonium cerium nitrate was dissolved in 2 mL water and added to the reaction liquid drop by drop for 18 h at room temperature. An amount of 30 mL saturated sodium chloride solution was added to the reaction solution and extracted with ethyl acetate (3 × 10 mL); the organic layer was dried and concentrated. The product was separated by flash column chromatography and then recrystallized to obtain the target compounds 53 mg. Synthesized as a yellow solid in 63% yield. ^1^H NMR (400 MHz, DMSO-*d*_6_) δ 11.93 (s, 1H), 8.25 (d, J = 3.4 Hz, 1H), 8.10 (d, *J* = 7.2 Hz, 1H), 8.04–8.00 (m, 1H), 7.88 (dd, *J* = 9.3, 2.5 Hz, 1H), 4.55 (s, 1H), 4.10–3.96 (m, 2H), 2.91 (s, 1H), 1.95 (s, 2H), 1.72 (d, *J* = 6.4 Hz, 2H), 1.54 (t, *J* = 19.2 Hz, 3H), 1.39 (s, 3H), 1.12 (t, *J* = 7.1 Hz, 3H). ESI-MS (*m*/*z*) calculated for C_21_H_23_F_2_N_6_O_3_ 445.17, found 445.18 [M+H]^+^, 467.15 [M+Na]^+^.

ethyl(1R,2S,3S,4R)-3-((5-fluoro-2-(6-fluoro-2-oxo-2,3-dihydro-1H-imidazo[4,5-b]pyridin-1-yl)pyrimidin-4-yl)amino)bicyclo[2.2.2]octane-2-carboxylate (**I**)

Compound **14** (50 mg,0.11 mmol) was dissolved in 1.4 mL tetrahydrofuran, and then 0.17 mL lithium hydroxide solution (2 M) was added drop by drop. The reaction was heated to 49 °C in the water bath, and the reaction was at constant temperature for 7 h until TLC showed the end of the reaction, and the reaction was cooled to room temperature for post-treatment. An amount of 2 M citric acid was added to adjust the pH to neutral. The aqueous layer was extracted with dichloromethane (40 mL × 3), and the combined organic layers were dried over anhydrous MgSO_4_ and concentrated in vacuo. The product was purified by flash column chromatography (petroleum ether/ethyl acetate, 8:1) to give the product (15.1 mg, 33% yield, 100.0% purity) as a white powder. ^1^H NMR (400 MHz, DMSO-*d*_6_) δ 8.20 (d, *J* = 3.4 Hz, 1H), 8.00 (s, 2H, exchangeable), 7.91 (d, *J* = 9.3 Hz, 1H), 4.59 (s, 1H), 2.69 (s, 1H), 2.00 (s, 1H), 1.84 (s, 1H), 1.67 (d, *J* = 35.0 Hz, 3H), 1.50 (s, 3H), 1.39–1.27 (m, 2H). ^13^C NMR (101 MHz, DMSO-*d*_6_) δ 157.05, 154.65, 152.90, 152.77, 150.50, 145.35, 139.11, 128.34, 128.08, 123.60, 107.90, 51.73, 48.79, 29.24, 28.73, 25.95, 24.24, 21.76, 19.81. ^19^F NMR (376 MHz, DMSO-*d*_6_) δ −136.90, −156.70. HRMS (ELI) calculated for C_19_H_19_F_2_N_6_O_3_ 417.1408, found 417.1482 [M+H]^+^.

tert-butyl (2-chloro-5-fluoropyridin-3-yl)carbamate (**16**)

We measured 90 mL anhydrous tert-butanol in a 250 mL round bottom flask and stirred while adding 2-chloro-5-fluoronicotinic acid (8.77 g, 50 mmol), and then 1eq TEA was added to stir evenly. Under the protection of nitrogen, 1.2eq DPPA was added drop by drop, and the reaction was completely monitored by TLC for 12 h at room temperature. The product was purified by flash column chromatography ((petroleum ether/ethyl acetate, 10:1) to give the product as a white solid. (5.587 g, 45.4%). ^1^H NMR (400 MHz, Chloroform-*d*) δ 8.39 (dd, *J* = 10.1, 2.7 Hz, 1H), 7.91 (dd, *J* = 2.8, 0.5 Hz, 1H), 7.04 (s, 1H), 1.53 (s, 9H). ESI-MS (*m*/*z*) calculated for C_10_H_13_ClFN_2_O_2_ 247.06, found 247.09 [M+H]^+^.

2-chloro-5-fluoropyridin-3-amine (**17**)

Add trifluoroacetic acid (3.8 eq) into 50 mL dichloromethane and stir well. Add compound **16** (5.587 g, 0.02 mmol) into the reaction system and heat up to the internal 40 °C to start the reaction, and the reaction ends after 27 h. First, the reaction solution was spun dry, and then 20 mL water and 40 mL ethyl acetate were added to neutralize the excess trifluoroacetic acid with saturated sodium bicarbonate solution. After neutralizing the acid, the organic phase was separated, and the anhydrous sodium sulfate was dried and spun and recrystallized with petroleum ether ethyl acetate, yielding 3.4 g white crystal product with a yield close to 100%.^1^H NMR (400 MHz, Chloroform-*d*) δ 7.66 (d, *J* = 2.6 Hz, 1H), 6.79 (ddd, *J* = 9.2, 2.6, 0.6 Hz, 1H), 4.03 (s, 2H). ESI-MS (*m*/*z*) calculated for C_5_H_5_ClFN_2_ 147.00, found 147.99 [M+H]^+^.

2-chloro-5-fluoro-3-nitropyridine (**18**)

Add 2.6 mL hydrogen peroxide to a 50 mL three-mouth bottle, stir and add 5.3 mL concentrated sulfuric acid to cool the system to 0 °C. Take to compound **17** (240 mg, 1.6 mmol) and dissolve it in 3 mL concentrated sulfuric acid, then add to the reaction system drop by drop. Gradually rise to room temperature until TLC detection of the reaction was complete. After lowering the temperature to −5 °C, concentrated ammonia was slowly added to the system, and the pH was adjusted to 7–8. The combined organic layers were dried over anhydrous MgSO_4_ and concentrated in vacuo. The product was purified by flash column chromatography to give the white solid (186 mg, 59.7%). ^1^H NMR (400 MHz, Chloroform-*d*) δ 8.58–8.52 (m, 1H), 8.04 (dd, *J* = 6.6, 2.8 Hz, 1H). ESI-MS (*m*/*z*) calculated for C_5_H_3_ClFN_2_O_2_ 176.98, found 176.01 [M+H]^+^

5-fluoro-*N*-(4-methoxybenzyl)-3-nitropyridin-2-amine (**20**)

Compound **18** and p-methoxylbenzylamine (2 eq) were added to 22 mL dioxane and cesium carbonate with 2 eq was refluxed at 101 °C for 3 h until TLC detection of the reaction was complete. Then, it was cooled to room temperature and the cesium carbonate solids were filtered out with diatomite. The filter cake was washed with DCM to reduce product loss. The white solid was separated by flash column chromatography (77.27%). ^1^H NMR (400 MHz, Chloroform-*d*) δ 8.40 (d, *J* = 2.9 Hz, 1H), 8.35 (s, 1H), 8.20 (dd, *J* = 8.0, 2.9 Hz, 1H), 7.29 (d, *J* = 8.6 Hz, 2H), 6.91–6.86 (m, 2H), 4.75 (d, *J* = 5.5 Hz, 2H), 3.81 (s, 3H). ESI-MS (*m*/*z*) calculated for C_13_H_13_FN_3_O_3_ 278.09, found 278.10 [M+H]^+^

5-fluoro-N2-(4-methoxybenzyl)pyridine-2,3-diamine (**21**)

Compound **20** (1 eq), ammonium formate (10 eq), potassium carbonate (4 eq), and iron powder (8 eq) were added to the mixture solvent of ethanol and water (5:1), and the reaction was completed by heating and reflux for 5 h. After cooling to room temperature, the iron powder was removed by filtration of diatomite, ethanol was removed by rotary steaming, and 30 mL saturated sodium chloride solution was added to the reaction solution and extracted with ethyl acetate (3 × 10 mL); the organic layer was dried and concentrated. The white solid was separated by flash column chromatography (79%).^1^H NMR (400 MHz, Chloroform-*d*) δ 7.62 (d, *J* = 2.6 Hz, 1H), 7.32 (d, *J* = 8.6 Hz, 2H), 6.91–6.86 (m, 2H), 6.69 (dd, *J* = 9.1, 2.6 Hz, 1H), 4.49 (s, 2H), 4.02 (s, 1H), 3.81 (s, 3H), 3.38 (s, 2H). ESI-MS (*m*/*z*) calculated for C_13_H_15_FN_3_O 248.11, found 248.09 [M+H]^+^

ethyl(1R,2S,3S,4R)-3-((5-fluoro-2-((5-fluoro-2-((4-methoxybenzyl)amino)pyridin-3-yl)amino)pyrimidin-4-yl)amino)bicyclo[2.2.2]octane-2-carboxylate (**23**)

ethyl(1R,2S,3S,4R)-3-((2-chloro-5-fluoropyrimidin-4-yl)amino)bicyclo[2.2.2]octane-2-carboxylate and (9,9-dimethyl-9H-xanthene-4,5-diyl)bis(diphenylphosphane), Cs_2_CO_3_, Pd(OAc)_2_ were added to the mixture solvent of 1,4-dioxane by microwave, and heat up to the internal 140 °C to start the reaction, and the reaction ends after 30 min. The white solid was separated by flash column chromatography (20%). ^1^H NMR (400 MHz, DMSO-*d*_6_) δ 8.41 (s, 1H), 8.07 (dd, *J* = 11.1, 2.7 Hz, 1H), 7.89 (d, *J* = 3.8 Hz, 1H), 7.66 (d, *J* = 2.7 Hz, 1H), 7.55 (d, *J* = 6.9 Hz, 1H), 7.27 (d, *J* = 8.6 Hz, 2H), 6.87 (d, *J* = 8.7 Hz, 2H), 6.64 (t, *J* = 5.2 Hz, 1H), 4.42 (d, *J* = 5.1 Hz, 2H), 4.08–4.00 (m, 2H), 3.72 (s, 3H), 2.88 (d, *J* = 6.8 Hz, 1H), 1.92 (s, 1H), 1.76 (s, 1H), 1.71 (d, *J* = 8.1 Hz, 2H), 1.61 (d, *J* = 5.9 Hz, 1H), 1.45 (d, *J* = 8.5 Hz, 2H), 1.38 (d, *J* = 11.1 Hz, 3H), 1.17 (t, *J* = 7.1 Hz, 1H), 1.13 (t, *J* = 7.1 Hz, 3H). ESI-MS (*m*/*z*) calculated for C_28_H_33_F_2_N_6_O_3_ 539.25, found 539.25 [M+H]^+^, 561.23 [M+Na]^+^.

ethyl(1R,2S,3S,4R)-3-((2-((2-amino-5-fluoropyridin-3-yl)amino)-5-fluoropyrimidin-4-yl)amino)bicyclo[2.2.2]octane-2-carboxylate (**24**)

Trifluoroacetic acid (8 eq) was added to dichloromethane and stirred evenly. Compound **23**(1 eq) and anisole (1 eq) were added to the reaction system and the reaction was finished at room temperature for 24 h. First, the reaction solution was spun dry, and then 20 mL water and 40 mL ethyl acetate were added to neutralize the excess trifluoroacetic acid with saturated sodium bicarbonate solution. After neutralizing the acid, the organic phase was separated. The product was purified by flash column chromatography ((petroleum ether/ethyl acetate, 1:1) to give the product **24** (white solid, 41.33%).^1^H NMR (400 MHz, DMSO-*d*_6_) δ 8.21 (s, 1H), 8.13 (d, *J* = 11.3 Hz, 1H), 7.88 (d, *J* = 3.8 Hz, 1H), 7.53 (d, *J* = 2.7 Hz, 2H), 5.81 (s, 2H), 4.03–3.96 (m, 2H), 3.56 (s, 1H), 2.84 (s, 1H), 1.95 (s, 1H), 1.90 (s, 1H), 1.74 (s, 1H), 1.67 (d, *J* = 7.9 Hz, 1H), 1.45 (d, *J* = 4.7 Hz, 2H), 1.35 (d, *J* = 5.1 Hz, 2H), 1.19 (s, 1H), 1.14 (d, *J* = 7.1 Hz, 1H), 1.11 (d, *J* = 8.3 Hz, 3H). ESI-MS (*m*/*z*) calculated for C_20_H_25_F_2_N_6_O_2_ 419.19 found 419.20 [M+H]^+^, 441.20 [M+Na]^+^.

ethyl (1R,2S,3S,4R)-3-((2-(2-amino-6-fluoro-1H-imidazo[4,5-b]pyridin-1-yl)-5-fluoropyrimidin-4-yl)amino)bicyclo[2.2.2]octane-2-carboxylate (**25**)

The intermediates **24** (1 eq) and bromoacetonitrile (3 eq) were added to the mixed solvent methanol:water (1:1) reaction system at 0 °C, and the reaction was kept at 0 °C for 15 min, followed by 24 h at room temperature. Intermediate **25** was obtained as a white solid, and the yield was less than 10%. ^1^H NMR (400 MHz, DMSO-*d*_6_) δ 8.39 (d, *J* = 7.3 Hz, 1H), 8.31 (d, *J* = 2.8 Hz, 1H, exchangeable), 8.28 (s, 1H, exchangeable), 8.27 (s, 2H, exchangeable), 8.12–8.08 (m, 1H), 4.65 (t, *J* = 7.4 Hz, 1H), 4.09 (dd, *J* = 7.1, 3.0 Hz, 2H), 3.04 (d, *J* = 6.8 Hz, 1H), 2.03 (s, 1H), 1.90 (s, 1H), 1.77 (t, *J* = 10.9 Hz, 2H), 1.63 (d, *J* = 9.8 Hz, 1H), 1.54 (s, 1H), 1.51–1.40 (m, 3H), 1.25 (d, *J* = 7.8 Hz, 1H), 1.16 (t, *J* = 7.1 Hz, 3H). ESI-MS (*m*/*z*) calculated for C_21_H_24_F_2_N_7_O_2_ 444.19, found 444.20 [M+H]^+^

(1R,2S,3S,4R)-3-((2-(2-amino-6-fluoro-1H-imidazo[4,5-b]20yridine-1-yl)-5-fluoropyrimidin-4-yl)amino)bicyclo[2.2.2]octane-2-carboxylic acid (**II**)

Compound **25** (50 mg, 0.11 mmol) was dissolved in 1.4 mL tetrahydrofuran and then 0.17 mL lithium hydroxide solution (2 M) was added drop by drop. The reaction was heated to 49 °C in a water bath, and the reaction was completed for 7 h at a constant temperature, and the reaction was cooled to room temperature for post-treatment. Add 2 M citric acid to adjust pH to neutral, extract with ethyl acetate, demulsify saturated sodium chloride solution, dry with anhydrous magnesium sulfate, concentrate the organic phase (reverse phase column, methanol: water = 2:1–3:1), and obtain the final product analogue **II** (20.5 mg, 45% yield, 97.8% purity) as a white powder. m.p. 315–317 °C. ^1^H NMR (400 MHz, DMSO-*d*_6_) δ 12.52 (s, 1H, exchangeable), 8.33–8.30 (m, 2H, exchangeable), 8.26 (s, 2H, exchangeable), 8.09 (s, 1H), 4.61 (s, 1H), 2.92 (d, *J* = 6.6 Hz, 1H), 2.05 (s, 1H), 1.89 (s, 1H), 1.76 (d, *J* = 9.0 Hz, 3H), 1.62 (d, *J* = 9.2 Hz, 1H), 1.52–1.39 (m, 4H). ^13^C NMR (101 MHz, DMSO-*d*_6_) δ 157.49, 155.81, 153.50, 152.83, 152.30, 144.54, 142.01, 137.93, 129.98, 124.70, 109.65, 52.66, 49.51, 29.06, 28.61, 25.95, 24.41, 21.85, 19.90. ^19^F NMR (376 MHz, DMSO-*d*_6_) δ −140.51, −157.23. HRMS (ELI) calculated for C_19_H_20_F_2_N_7_O_2_ 416.1668, found 416.1640 [M+H]^+^

### 4.2. Surface Plasmon Resonance (SPR) Analysis

Commercial companies express and purify HIN1 PB2 CBD protein, which provides influenza A/WSN/1933 (H1N1) PB2 CBD (aa.318-486). Using a Biacore T200 (GE Healthcare) and a running buffer of which contains a solution of 1 × PBS-P + 0.02 M phosphate buffer, 2.7 mM KCl, 0.137 M NaCl, and 0.05% Tween 20, H1N1 PB2 was immobilized to a level of 4900 response units (RUs). Serial dilutions of small molecules were injected, ranging in concentration from 20 µM to 0.156 µM. GE Healthcare’s Biacore Evaluation Software was used to fit the resulting data to the affinity binding model.

### 4.3. Preparation of Influenza Virus H1N1 (A/PuertoRico/8/1934)

MDCK cells, a sensitive cell line suitable for the growth of influenza virus, were selected as virus-infected cells, DMEM + 0.2% BSA + 2 µg/mL TpcK-trypsin was selected as the virus maintenance solution. Nine-day-old chicken embryos were cultured for 2–3 days at 37 °C with virus storage fluid inoculated into their allantoic cavities. Viruses from allantoic fluid were harvested, centrifuged, and stored at −70 °C. A 10-fold gradient dilution of the virus venom was inoculated in MDCK cells with three multiple holes per gradient. In culture at 37 °C for three days, cytopathic changes were observed, and half of the virus infection (TCID50) was calculated using the Reed–Muench method.

#### 4.3.1. Cells and Viruses

The MDCK cells, purchased from the American Type Culture Collection (ATCC, Manassas, VA), were cultured in a humidity-saturated cell culture incubator at 37 °C and 5% CO_2_. Cells were usually passaged at a ratio of 1:3–1:6 for approximately 1–2 days (cells were spread on a monolayer) with 0.25% EDTA trypsin digestion for 8–10 min; the growth medium was DMEM high sugar medium containing 10% fetal bovine serum. Influenza A/Puerto Rico/8/1934 (H1N1) (PR/8) and A/Hongkong/8/68 (H3N2) (HK/68) were stored in our lab. The influenza virus growth medium was DF-12 medium containing 2 μg/mL TPCK trypsin.

#### 4.3.2. CPE Inhibition Assay

CPE inhibition assay was used to evaluate the antiviral activities of the candidate compounds against influenza A/Puerto Rico/8/1934 (H1N1) and HK/68 (H3N2) viruses in Madin–Darby canine kidney (MDCK) cells. Positive controls included oseltamivir carboxylate (OC) and VX-787. Twenty-four hours before infection, MDCK cells were seeded into 96-well plates at a density of 3 × 104 cells per well in the cell culture medium of DMEM + 10% FBS at 37 °C and 5% CO_2_. Before PBS washing twice, the medium was changed to DF-12 medium containing 2 µg/mL TPCK-trypsin. We infected cells with the influenza virus at an MOI of 0.005 with various concentrations (ranging from 0.005 µM to 100 µM by a three-fold dilution) of the test compound. CellTiter-Glo viability assay (Promega) was used to measure antiviral activity of test compounds after 72 h incubation at 37 °C in CO_2_ incubator. The concentration for 50% maximal effect (EC_50_) was calculated by Origin 8 software.

#### 4.3.3. Cytotoxicity Assay

Following the instructions in the kit manual, CellTiter-Glo viability assay was used to assess the cytotoxicity of compounds in MDCK cells. A confluent monolayer was grown in 96-well plates for 18–24 h at a density of 1.5 × 104 cells per well. Test compounds were added to cells using a three-fold dilution series in DF-12 medium containing 2 µg/mL TPCK-trypsin, with DMSO added as a control. SpectraMax M5 microplate readers (Molecular Device) were used to read the luminescence after 72 h incubation at 37 °C in CO_2_ incubator. The 50% cytotoxicity concentration (CC_50_) was calculated by Origin 8 software.

#### 4.3.4. Experimental Steps of Liver Microsome Metabolism In Vitro

As previously described, the CYP-mediated metabolic stability was evaluated as test compounds (with a final concentration of 1 μM) incubated with pooled rat or human liver microsomes (0.2 mg/mL protein) in 100 mM of potassium phosphate buffer with 3 mM of MgCl_2_, at pH 7.4 [41]. After pre-incubating for 5 min at 37 °C, the reaction was initiated with NADPH (at a final concentration of 1 mM). Negative control without NADPH and positive control with cocktail probe compounds (phenacetin, diclofenac, S-mephenytoin, bupropion, amodiaquine, dextromethorphan, and midazolam) were conducted simultaneously. The AO-mediated metabolic stability was evaluated as test compounds (with a final concentration of 1 μM) incubated with 37 °C pre-incubated pooled human hepatocyte cytosol (0.5 mg/mL protein) in 100mM of potassium phosphate buffer at pH 7.4, with or without AO inhibitor raloxifene (with a final concentration of 5 μM) [42]. Aliquots from the incubations were removed at different time points in the duration of 60 min and added into 5×volume prechilled internal standard-acetonitrile solution to stop the reactions. In order to prepare the supernatant for LC-MS/MS analysis, the supernatant was centrifuged at 15,000× *g* for 10 min and stored at −20 °C.

### 4.4. Chemoinformatics

The study was carried out on a workstation with an Intel^®^ Xeon (R) Platinum 8280L @2.26 GHz × 112 processor, 187.5 GB of RAM, an NVIDIA Corporation TU104 GPU, and a 4.5 TB hard drive running in Linux operating system. Bioinformatics software, Schrödinger (2018), and Gromacs 2021.03.

#### 4.4.1. Methods of Molecular Docking

The molecular docking procedure was performed using GlideXP in Maestro version 10.7 (Schrodinger LLC, New York, NY, USA) with the default option. Two-dimensional structure files in SDF format for VX-787, comp. **I**, and comp. **II** were generated using the 2D Sketcher of the Schrodinger software package. Then, an OPLS3 force field was applied to produce low-energy conformers, and possible states and tautomers at pH 7.0 ± 2.0 were generated using the LigPrep module (Schrodinger “suite”).

The crystal structure (PDB 5WL0) of influenza A H3N2 bound to VX-787 were downloaded from the protein data bank. The raw PBD protein structure was prepared by using the Protein Preparation Wizard (Schrodinger), adding hydrogen atoms, refining the loop region, optimizing H-bond assignment, and finally restrained energy minimization (hydrogens only) by using an OPLS-2005 force field and tautomers at pH 7.0 ± 2.0. The glide-grid was generated using the Receptor Grid Generation module. The site for docking analysis was generated using the Structural coordinates of the co-crystallized ligand VX-787. The center of VX-787 (-24.06(X), 6.77 (Y) 10.63 (Z)), was designated as the grid center, the innerbox was set to 10’10’10 (angStroms), the outerbox was set to 23.21’23.21’23.21 (angStroms). No water molecules remain in the protein, no constraints, no rotatable groups and exclude volume were set.

The docking procedure was carried out using the unchanged conformation of the receptor and flexible ligand molecules. Types of the interaction of the docked PB2 with ligand were analyzed and then the docking conformations were selected and saved based on the calculated Glide docking energy score.

#### 4.4.2. Molecular Dynamics Simulations

All molecular dynamics simulations were carried out using the Gromacs program version 2021.3 [35]. The PB2/ligand complex models were simulated via molecular dynamics using the AMBER force field99SB-ildn for the protein. To generate the ligand topologies for Gromacs, the program Acpype [43] and Ambertools21 [44] was used. The ligands were treated with generalized Amber force-field (GAFF) and the restrained electrostatic potential (RESP2) methodology was implemented for the derivation of partial atomic charges, as recommended [45]. The calculation of the RESP atom charge of ligands in both vacuum and water is completed by Multiwfn [46]. The rectangular box dimensions for periodic boundary conditions while keeping a minimum distance from any atom to the boundary of the box at 1 nm were calculated to be 8.8 nm × 8.0 nm × 6.4 nm. The TIP4P water model was used to conduct the MD simulations in explicit solvation. The Steepest descent algorithm was used for energy minimizations, and the maximum force Fmax was set not to exceed 1000 kJ mol^−1^ nm^−1^. The system was equilibrated with a 300 K temperature and a 1 bar pressure by two consecutive 1000 ps simulations with canonical NVT ensembles and isobaric NPT ensembles, respectively. Molecular dynamics simulations were run for 100 ns with stable temperature and pressure with a time step of 2 fs and long-range interaction cutoff of 1 nm.

#### 4.4.3. Trajectory Clustering Analysis

The trajectories from 500 ps to 100 ns were selected for clustering analysis. The cutoff value was set to 0.25 and the analysis method is gromos. In total, 32 clusters for PB2/VX-787 complex, 50 clusters for PB2/comp. **I** complex, and 36 clusters for PB2/comp. **II** complex were generated. The structure with the most neighbors in the structural cluster was selected as the representative structure for each complex and superposed with the initial conformations (i.e., the docking conformations of PB2 with VX-787, Comp **I**, Comp **II**) for visual inspection.

#### 4.4.4. Estimation Binding-Free Energy Using MM/PBSA

Molecular mechanics/Poisson–Boltzmann surface area (MM/PBSA) in gmx_MMPBSA tools [37,47] inbuild with GROMACS package was applied to determine the thermodynamical stability of the ligand inside the binding sites of the targets to inspect the contribution of each residue of the binding pockets. A total of 1000 frames from the last 10 ns of the MD trajectory after equilibrium were used to calculate the binding energy. The average contribution of the residues to the binding energy was calculated for each complex using different parameters, istrng = 0.15, fillratio = 4.0, radiopt = 0, inp = 1. Finally, the Python script MMPBSA.py has been used for the statistical analysis of binding-free energy and the graphical tool XMGRACE has been used for trajectory analysis.

## 5. Conclusions

In this study, in order to obtain PB2 inhibitors with good antiviral activities and high AO metabolic stabilities, two 2-substituted-2,3-dihydro-imidazopyridine VX-787 analogs were designed, synthesized, and evaluated for their anti-influenza virus activities. CPE inhibition assays showed that comp. **I** and comp. **II** were able to protect cells from influenza virus-mediated death at non-cytotoxic concentrations with EC_50_ values of 0.04 μM and 0.09 μM, respectively. A concentration-dependent association and dissociation pattern was observed in protein binding affinity assays, with K_D_ values of 1.398 μM and 1.670 μM, respectively. Metabolic stability analysis showed that, in addition to exhibiting good stability in liver microsomes, comp. **I** and comp. **II** were significantly less sensitive to AO than VX-787. These results confirmed that VX-787 is indeed a substrate of aldehyde oxidase and the metabolic stabilities of comp. **I** and comp. **II** to AO were significantly higher than that of VX-787. Unfortunately, comp. **I** and comp. **II** were less active against influenza than VX-787. Molecular docking, MD simulations, and MM/PBSA analyses results showed that, although the binding modes of the comp. **I** and comp. **II** are similar to that of VX-787, comp. **I** and comp. **II** had lower structural adaptability to PB2 than VX-787. MM/PBSA analysis also suggested that the binding of VX-787, comp. **I**, and comp. **II** to PB2 was mainly driven by van der Waals interactions, whereas the difference in electrostatic (polar) interactions was the main reason for a better affinity of VX-787 to PB2 than comp. **I** and **II**. When designing PB2 inhibitors, special attention should be paid to the adaptability of the overall structure of the inhibitor molecule to the structure of the PB2 protein, particularly the adaptability of the electrostatic (polar) interaction of the inhibitor to the PB2 protein. At the same time, the polar surface area of the molecule should be minimized to maximize the van der Waals interactions with the PB2 protein

## Figures and Tables

**Table 1:** Structures, docking scores, anti-H1N1 and -H3N2 activities, cytotoxicities, and K_D_ values of the active compounds.

Compounds	Docking Score	H1N1 IC_50_ (μM)	H3N2 IC_50_ (μM)	CC_50_ (μM)	SPR K_D_ (μM)
OC	-	2.12 ± 0.03	0.04 ± 0.04	>200	-
VX-787	−11.519	<0.003	<0.003	>200	0.152
comp. **I**	−10.550	0.07 ± 0.02	0.04 ± 0.01	>200	1.398
comp. **Ⅱ**	−10.475	0.09 ± 0.05	0.07 ± 0.03	>200	1.670

The data represent averages of three independent determinations.

**Table 2:** Mean pharmacokinetic parameters of VX-787 and comp. **I**/**II** in liver cytoplasms or microsomes of two species (human/rat). Metabolic stability assay using mouse liver microsomes.

Compounds	Microsome				Cytoplasm
	T_1/2_^a^ (min)		Cl_h_^b^ (mL/min/kg)		T_1/2_ (min)
	Rat	Human	Rat	Human	Human
VX-787	349.29	249.46	1.72	1.49	150
comp. **I**	>800	>800	0.53	<0.1	>800
comp. **II**	>800	>800	<0.1	0.46	>800

a: T_1/2_ is the half-life. b: Cl_h_ is the clearance. All experiments were performed independently at least three times (mean ± SD). The data are averages of three independent determinations.

## Data Availability

The data presented in this study are available in the references.

## Supplementary Materials

The following are available online https://www.mdpi.com/article/10.3390/molecules28041849/s1. Figure S1: Comparison between the pose of VX-787 and the original pose in the crystal structure; Figure S2: Comparison between the pose of VX-787, comp. **I** and comp. **II** in the crystal structure; Figure S3: Comparing the representative conformations (red) of MD simulations and the docking conformations (blue) of PB2 in complex with VX-787(A), Comp. **I** (B), and Comp. **II** (C); Figure S4 (S4-1–S4-44): The NMR and ESI-MS Spectra of compound **6**–**25** and Comp **I** and **II**; Figure S5 (S5-1–S5-2): HPLC analysis of Comp. **I** and comp. **II**; Figure S6 (S6-1–S6-2): Chiral HPLC analysis of Compound **12**; Table S1: Residual percentage of VX-787 and Comp. **I**/**II** in rat liver microsome; Table S2: Residual percentage of VX-787 and comp. **I**/**II** in human liver microsome; Table S3: Residual percentage of VX-787 and comp **I**/**II** in 0.5mg/mL human liver cytoplasm with and without the AO inhibitor, Raloxifene; Table S4: Calculated Binding Free Energies by the MM/PBSA Method (All in kcal/mol); Table S5: Per-residue binding energy decomposition of VX-787, comp. **I** and comp. **II**.

## Sample Availability

Samples of the compounds are not available from the authors.
