# Supplementary material for: Design, Synthesis, Biological Evaluation, and Molecular Dynamics Simulation of Influenza Polymerase PB2 Inhibitors
Source: Molecules. 2023 Feb 15;28(4):1849. doi: 10.3390/molecules28041849 (PMC9960307; doi:10.3390/molecules28041849)
Supplement: Supplementary file 1 [file molecules-28-01849-s001.zip › molecules-2035303-supplementary.pdf]

Design, Synthesis, and Biological Evaluation of Influenza Polymerase PB2 Inhibitors

Authors

Xinhong Li[aξ], Yijie Xu[aξ], WeLi[aξ], Jinjing Che[aξ],Xu Zhao[bc], Ruyuan Cao[a]*, Xingzhou Li[a]*, SongLi [a]*

Affiliations

[a]Beijing Institute of Pharmacology and Toxicology, 27 Taiping Road, Beijing 100850, China

[b] Department of Hepatology, Fifth Medical Center of Chinese PLA General Hospital, Beijing, China

[c] China Military Institute of Chinese Materia, Fifth Medical Center of Chinese PLA General Hospital, Beijing, China

[ξ] These authors contribute equally; *Correspondence author

Supporting information

Table.S1 Residual percentage of VX-787 and comp. I/II in rat liver microsome

Time (min)	VX-787		Comp.I		Comp.II	
	With NADPH	Without NADPH	With NADPH	Without NADPH	With NADPH	Without NADPH
0	99.99±0.99	99.99±2.01	100.00±2.49	100.00±10.20	100.00±4.38	100.00±8.79
5	98.25±4.53	109.05±1.48	94.84±5.74	99.15±11.37	108.93±4.89	111.54±10.34
15	95.80±0.68	103.93±4.79	89.21±4.57	101.81±8.58	112.81±6.28	110.83±2.33
30	91.51±3.09	100.41±4.32	93.67±2.64	102.83±7.53	101.58±12.69	109.70±6.48
60	88.79±2.11	92.13±5.76	93.35±1.14	103.04±8.53	111.32±5.88	98.23±6.99

Table.S2 Residual percentage of VX-787 and comp. I/II in human liver microsome

Tim (min)	VX-787		Comp.I		Comp.II	
	With NADPH	Without NADPH	With NADPH	Without NADPH	With NADPH	Without NADPH
0	99.99±4.23	100.00±0.81	100.00±3.28	100.00±6.09	99.99±7.02	100.00±6.83
5	103.84±1.21	110.26±0.79	100.49±6.61	114.92±3.30	94.51±9.30	99.31±7.43
15	91.56±7.20	100.78±10.11	106.58±9.83	104.78±5.87	97.92±3.72	104.43±6.89
30	101.85±8.49	113.32±4.83	100.90±12.88	99.87±3.65	98.09±10.21	98.40±5.82
60	89.33±8.96	107.81±9.16	93.07±4.49	94.46±2.89	92.65±4.06	102.23±6.80

Table S3. Residual percentage of VX-787 and Comp I/II in 0.5mg/ml human liver cytoplasm with and without the AO inhibitor, Raloxifene.

t (min)	VX-787	VX-787 (Raloxifene)	Comp I	Comp I (Raloxifene)	Comp II	Comp II (Raloxifene)
0	100.00±7.65	100.00±3.54	100.00±6.12	100.00±3.87	100.00±0.44	100.00±8.74
15	87.02±9.00	100.65±7.76	95.20±3.72	108.27±5.07	107.07±16.04	110.60±4.82
30	90.17±9.31	97.25±1.77	99.98±8.74	102.66±4.43	108.17±9.51	107.60±7.80
60	71.87±6.78	103.95±4,66	93.52±8.82	112.89±1.99	103.11±2.84	109.12±6.67

Table S4. Calculated Binding Free Energies by the MMPBSA Method (All in kcal/mol)

Complex	ΔE_{vdw}	ΔE_{ele}	ΔE_{pb}	ΔE_{surf}	ΔG_{bind}
VX-787	-45.91±0.36	-247.11±2.15	251.70±4.11	-4.28±0.01	-45.60±4.65
**I**	-49.70±0.80	-220.05±0.73	234.00±0.35	-4.31±0.02	-40.06±1.14
Ⅱ	-45.24±1.15	-211.49±4.01	234.87±0.27	-4.41±0.01	-26.27±4.18

Table S5. Per-residue binding energy decomposition of VX-787, compounds **I** and **II**

AA	VX-787		Comp I		Comp II	
	mean	SD	mean	SD	mean	SD
PHE:323	-2.45105	0.070451	-1.4611	0.079696	-1.52427	0.080004649
SER:324	0.949623	0.081537	0	0	0.551919	0.090021782
PHE:325	-0.51746	0.074013	0	0	-0.62683	0.081708725
ARG:332	0	0	-3.99818	0.238226	-2.03536	0.239039551
SER:337	-0.53609	0.104914	0.042302	0.092	-0.10503	0.079342268
ARG:355	-5.33388	0.291131	-4.36968	0.293115	-4.86402	0.306079758
HIS:357	-0.65673	0.08837	-1.47108	0.101679	-1.33227	0.097769133
GLU:361	-3.59706	0.204837	-1.99955	0.163666	-2.46656	0.263712761
PHE:363	-0.69731	0.070858	-1.22252	0.07494	-0.92988	0.077776686
LYS:376	-1.58935	0.188195	-1.20223	0.165018	0	0
PHE:404	-0.92445	0.067283	-0.13053	0.075936	0.296917	0.075451186
GLN:406	-0.55772	0.106038	-0.25742	0.090332	-0.06444	0.097763565
ASN:429	0	0	-0.34507	0.105826	0	0
MET:431	-0.86446	0.07481	-0.8781	0.074744	-1.11121	0.075814767
HIS:432	0.157584	0.099729	0	0	0	0
ASN:510	-1.43966	0.210984	-1.35923	0.105682	-0.33357	0.172632481
VAL:511	-1.02466	0.063237	0.405107	0.075977	0.906425	0.079220643
LEU:512	-0.10143	0.067931	0	0	-0.02067	0.080911801
compound	-22.6768	0.2687	-18.3681	0.282442	-11.3799	0.278711258

Verification of docking method

Figure S1. Comparison between the docking pose of VX-787 and the original pose in the crystal structure, in which the calculated RMSD value was 0.2636 Å. The original pose was presented as a green stick, the predicted highest ranking pose was presented as a blue stick.

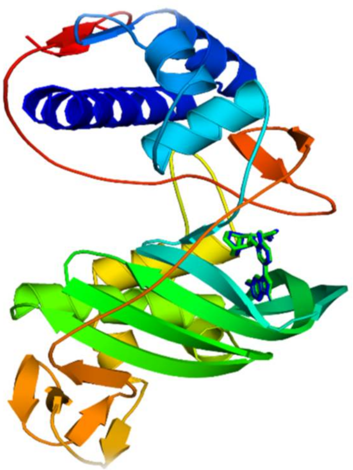

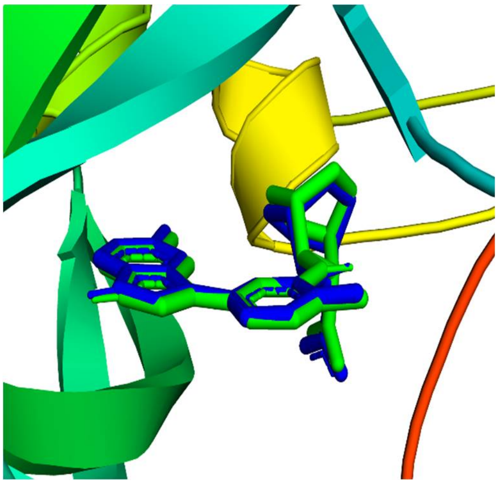

Figure S2. Comparison between the docking pose of comp. I ,comp. II and the original pose of VX-787 in the crystal structure. The RMSD of these two poses from the original pose of VX-787 were 0.6617 Å and 2.3213 Å, respectively. The VX-787 pose was presented as a green stick, the Comp. I pose was presented as a blue stick. the Comp. II pose was presented as a red stick.

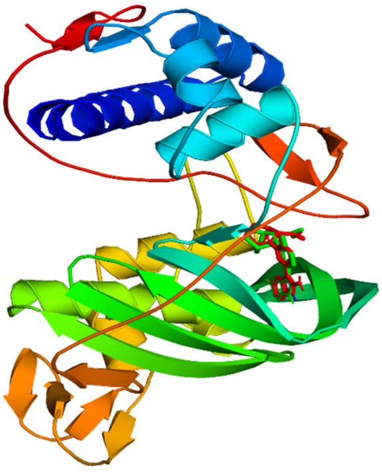

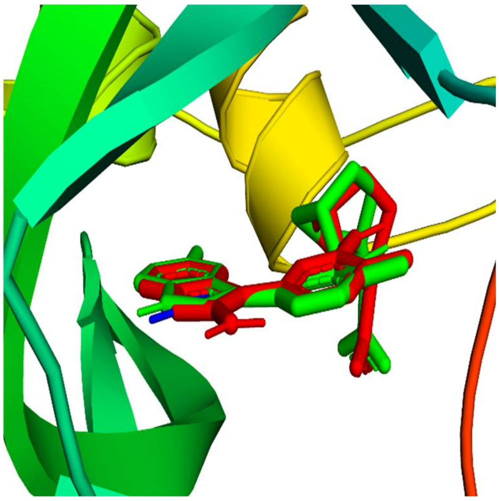

Figure S3. .Comparing the representative conformations (red) of MD simulations and the docking conformations (blue) of PB2 in complex with VX-787 (A), Comp. I (B), and Comp. II (C). The π-π stacking interaction is shown as a green dashed line, the intermolecular hydrogen bond as a magenta dashed line, and the salt bridge as a blue dashed line.

A

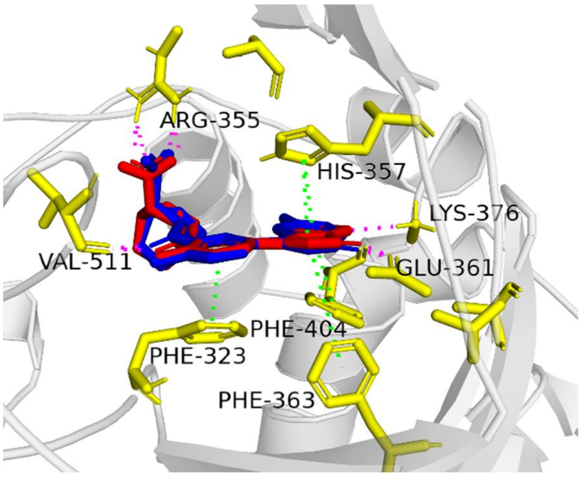

B

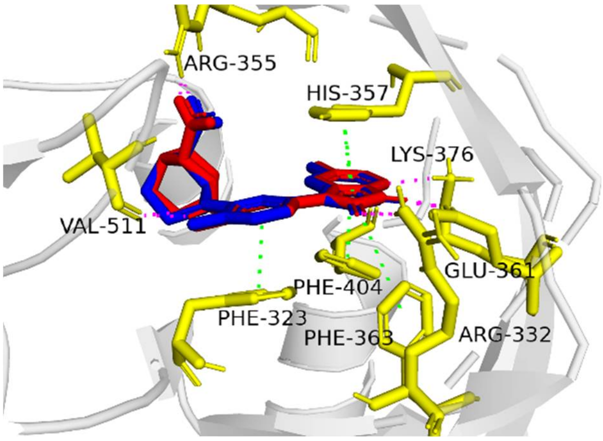

C

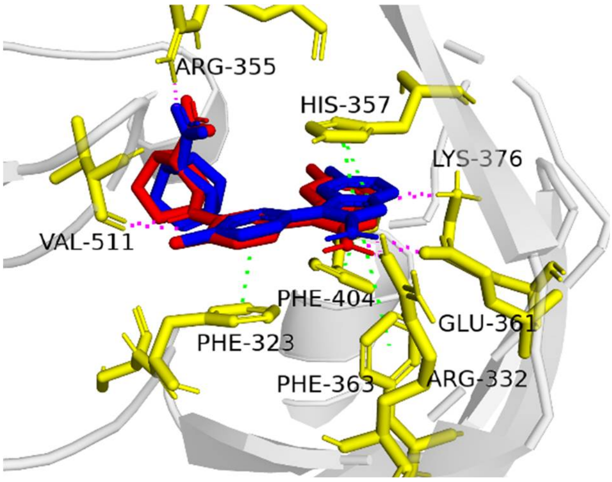

Figure S4. The NMR and ESI-MS Spectra of compound **6-25** and Comp. **I** and **II**

Figure S4-1. The ^{1}H-NMR Spectra of compound **6**

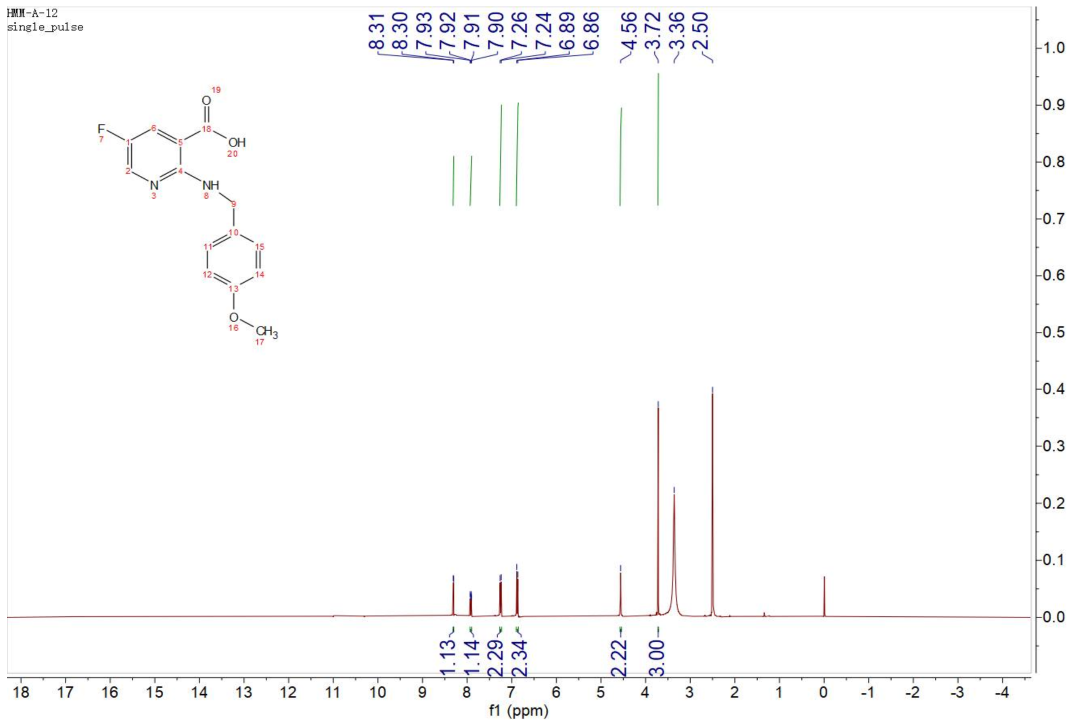

Figure S4-2. The ESI-MS Spectra of compound **6**

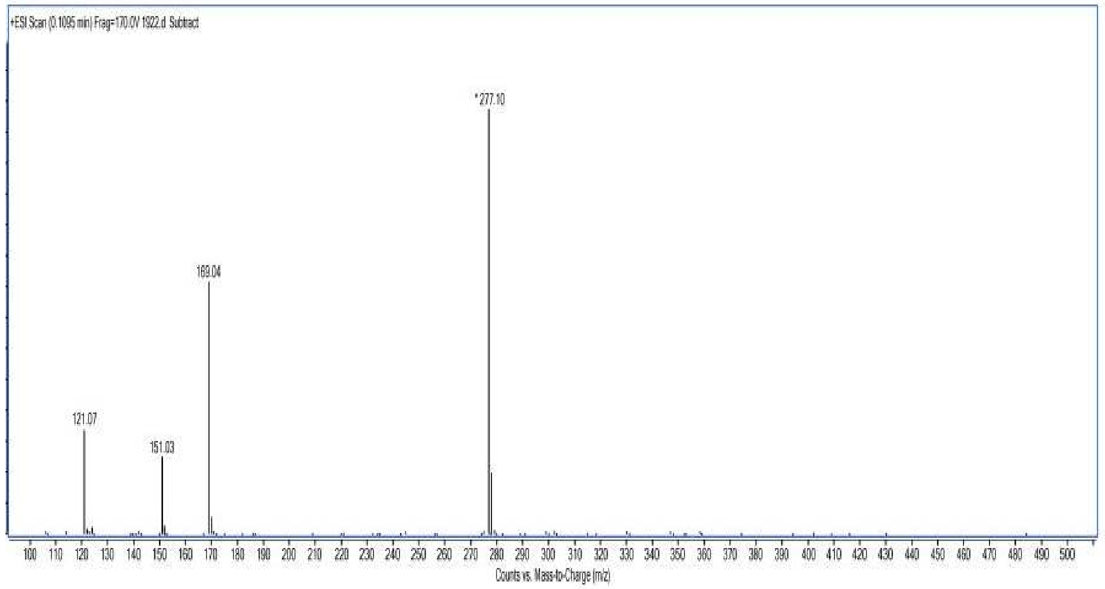

Figure S4-3. The ^{1}H-NMR Spectra of compound **7**

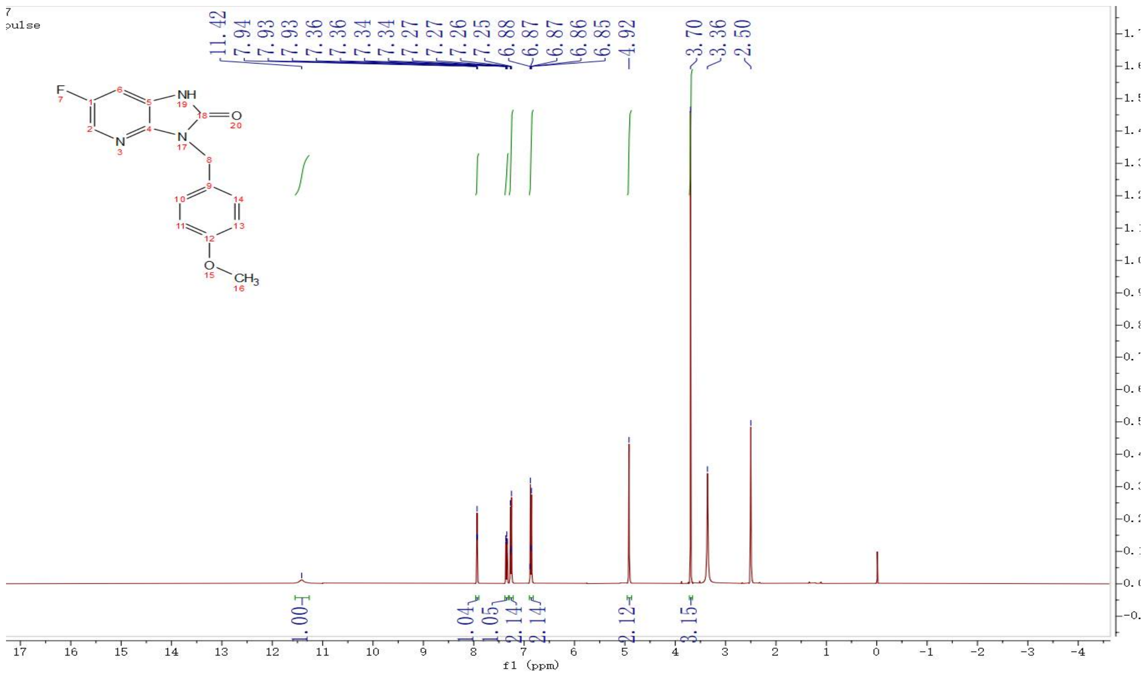

Figure S4-4. The ESI-MS Spectra of compound **7**

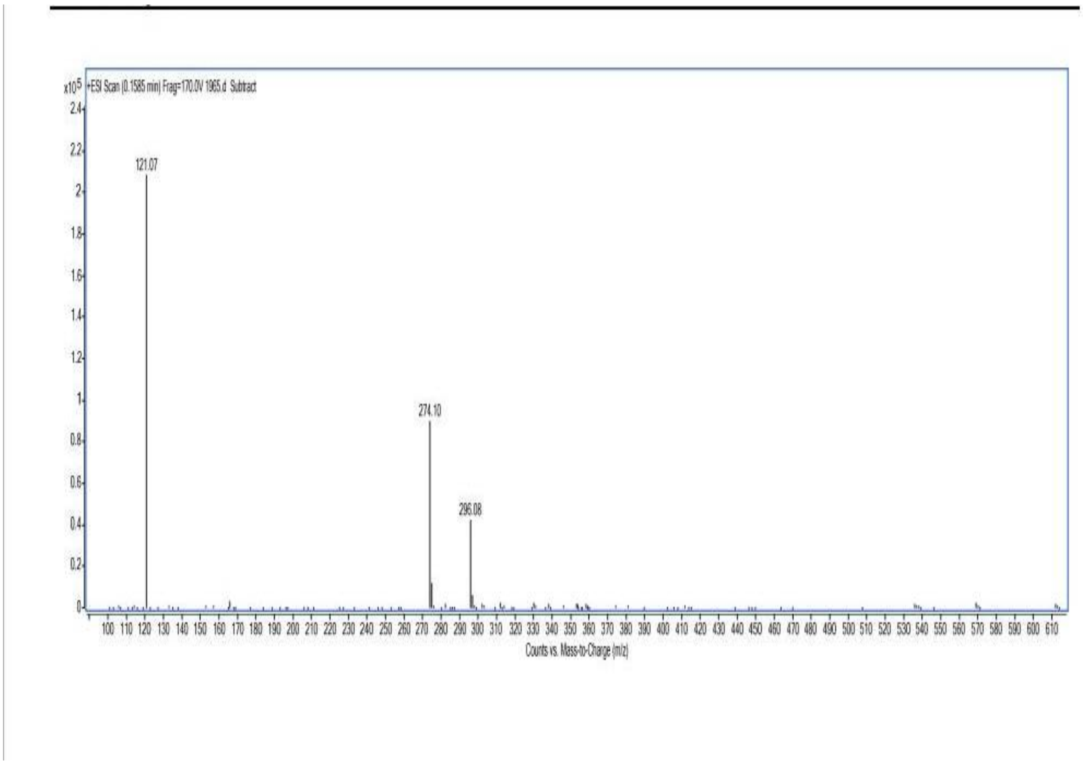

Figure S4-5. The ^{1}H-NMR Spectra of compound **8**

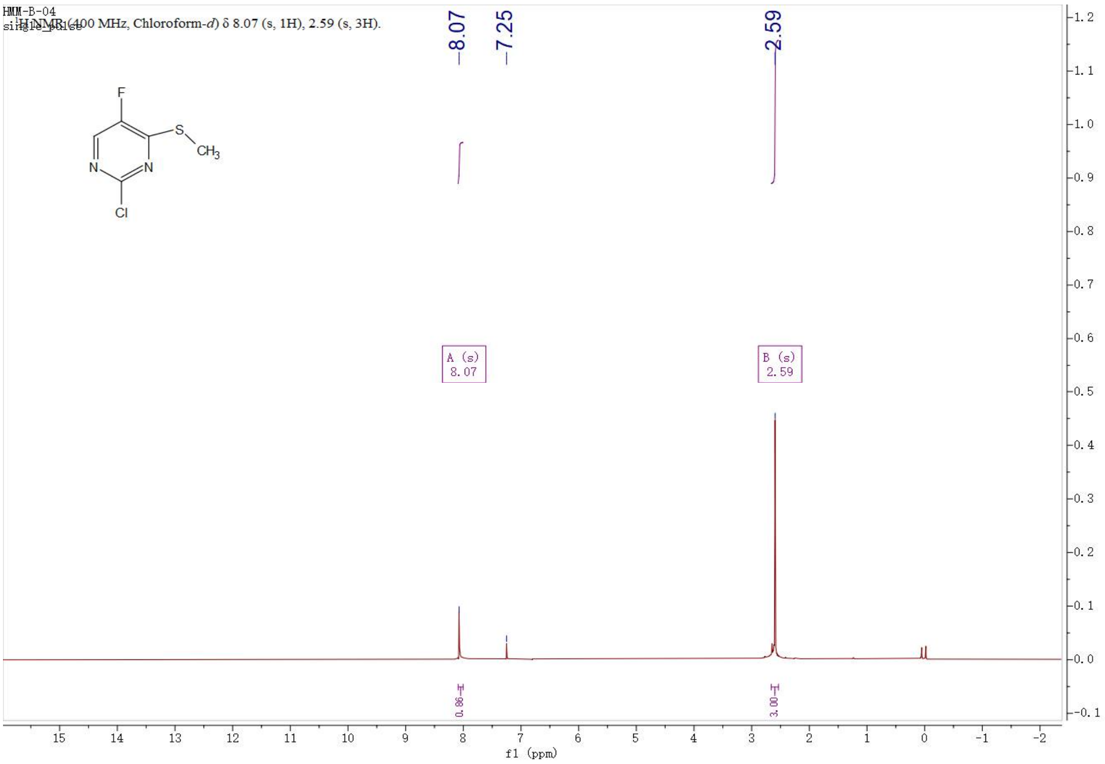

Figure S4-6. The ^{1}H-NMR Spectra of compound **9**

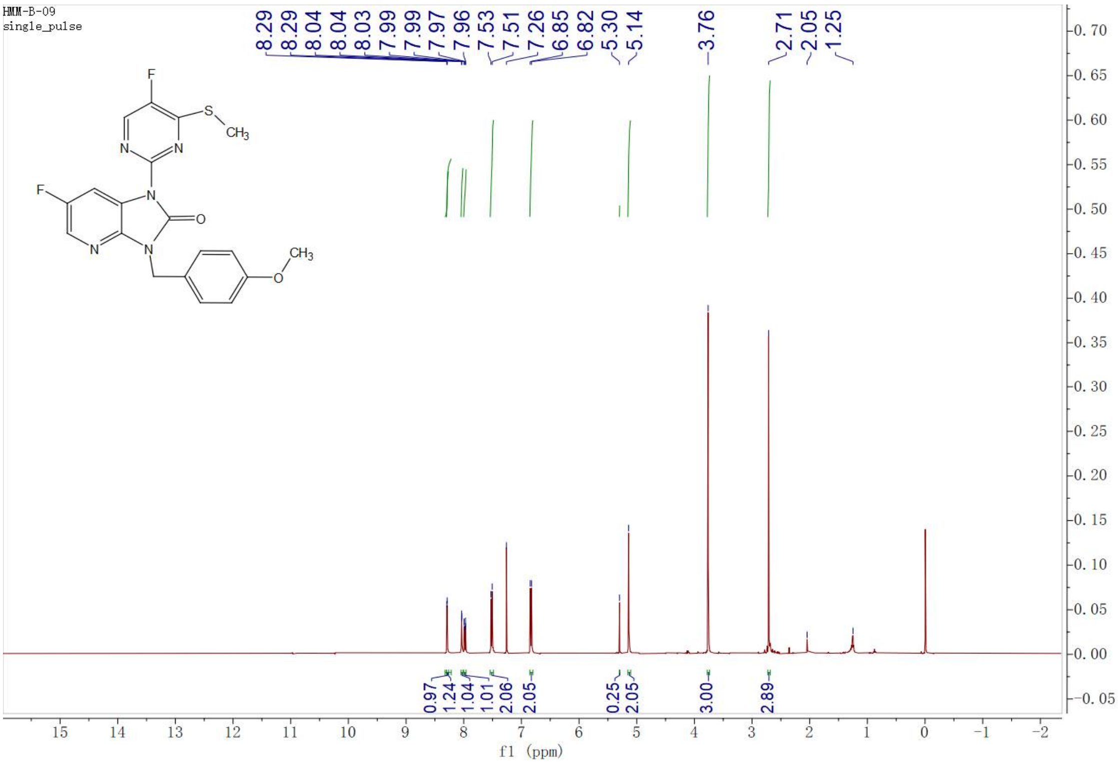

Figure S4-7. The ESI-MS Spectra of compound **9**

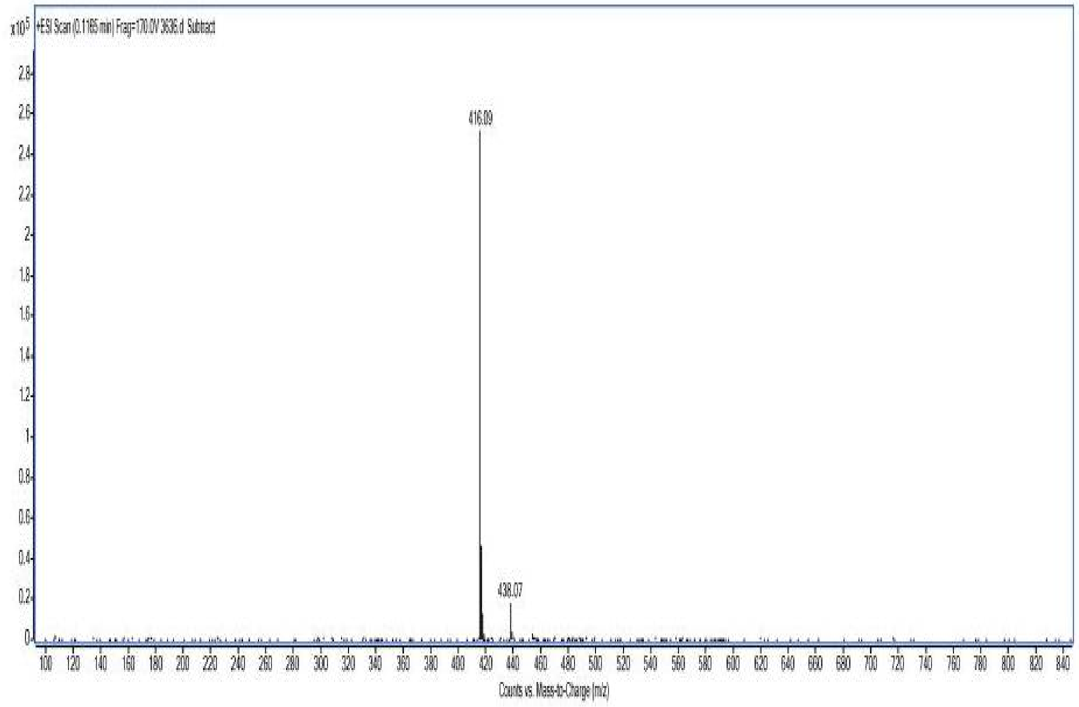

Figure S4-8. The ^{1}H-NMR Spectra of compound **11**

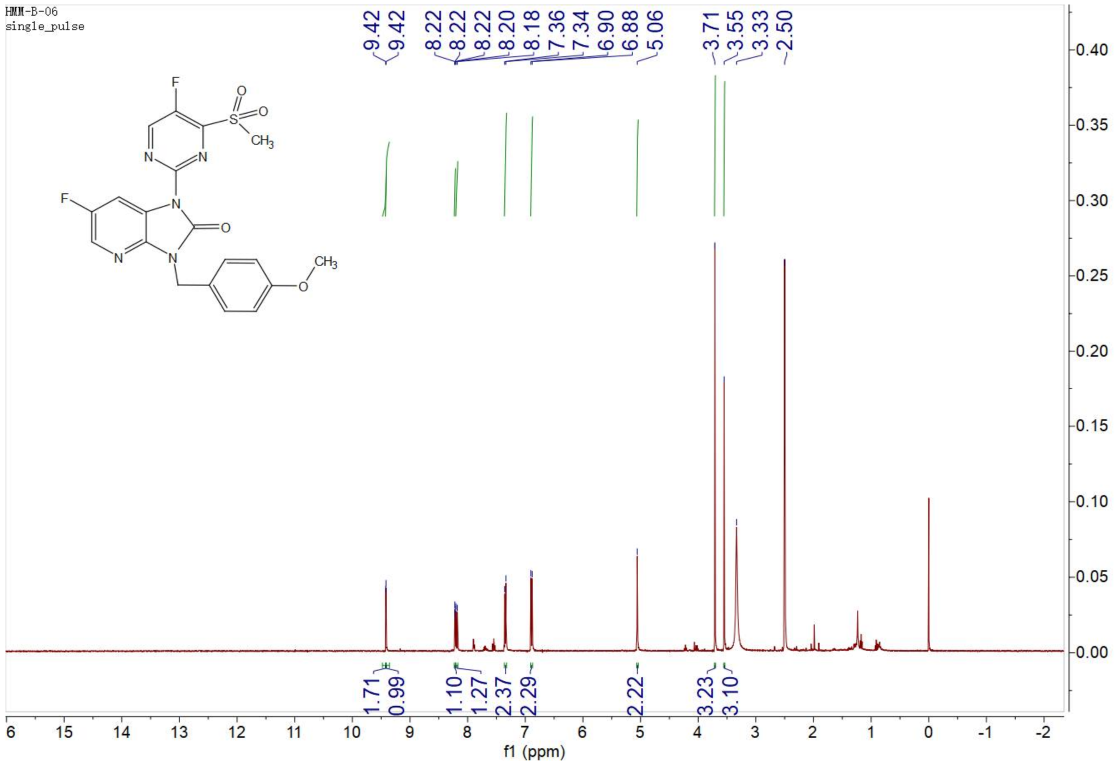

Figure S4-9. The ESI-MS Spectra of compound **11**

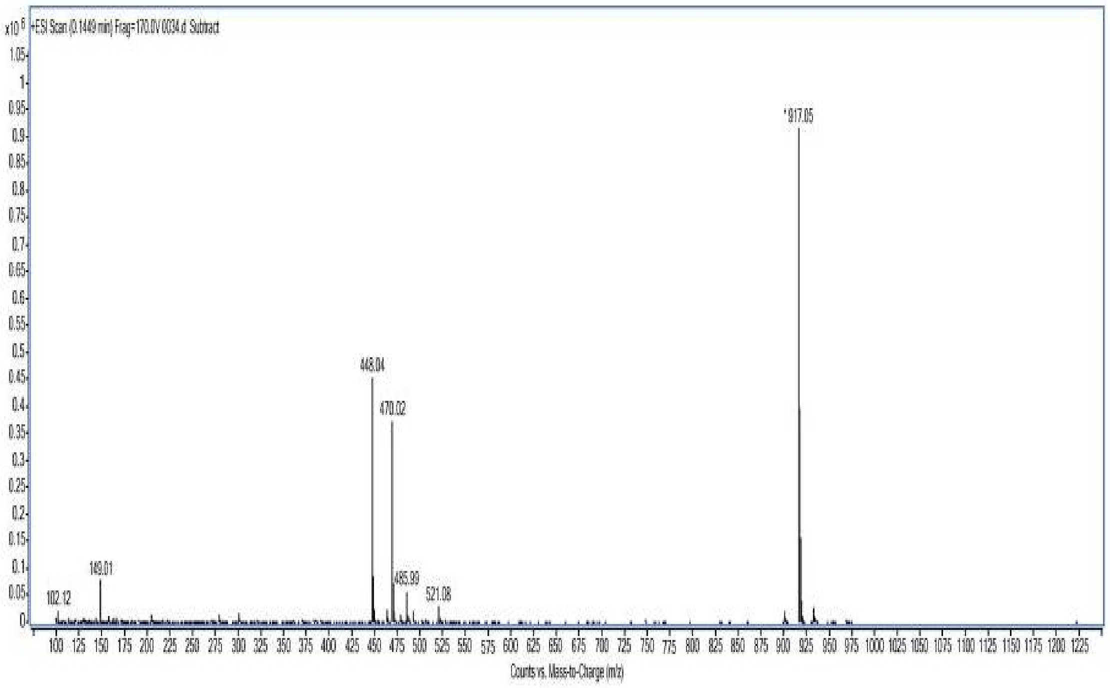

Figure S4-10. The ^{1}H-NMR Spectra of compound **12**

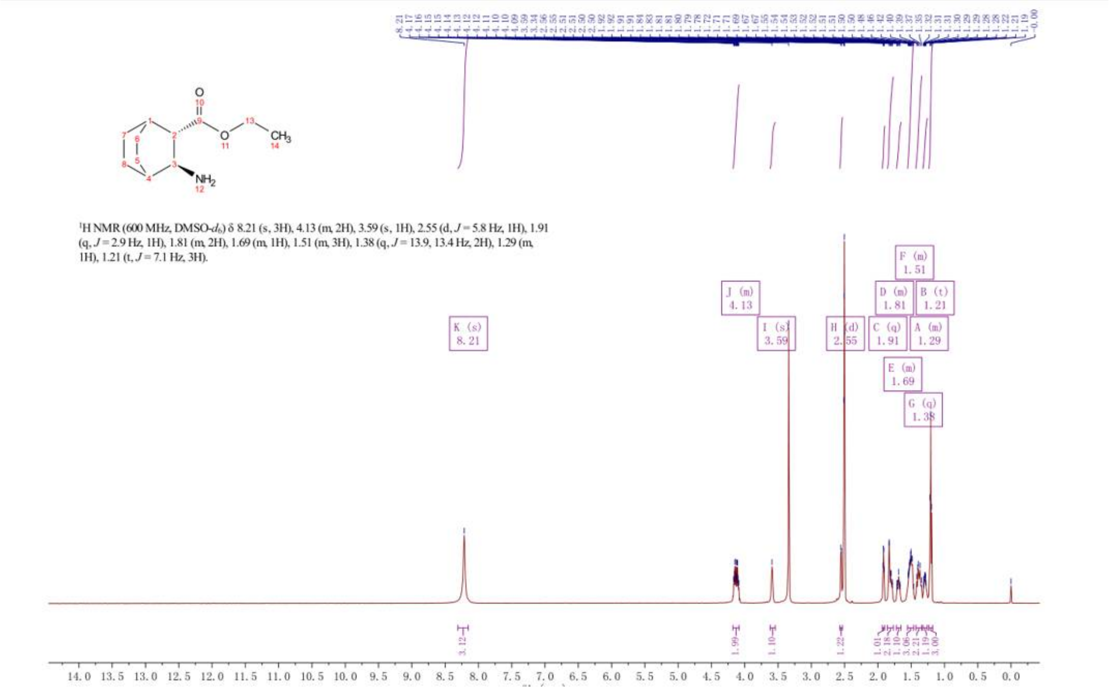

Figure S4-11. The ESI-MS Spectra of compound **12**

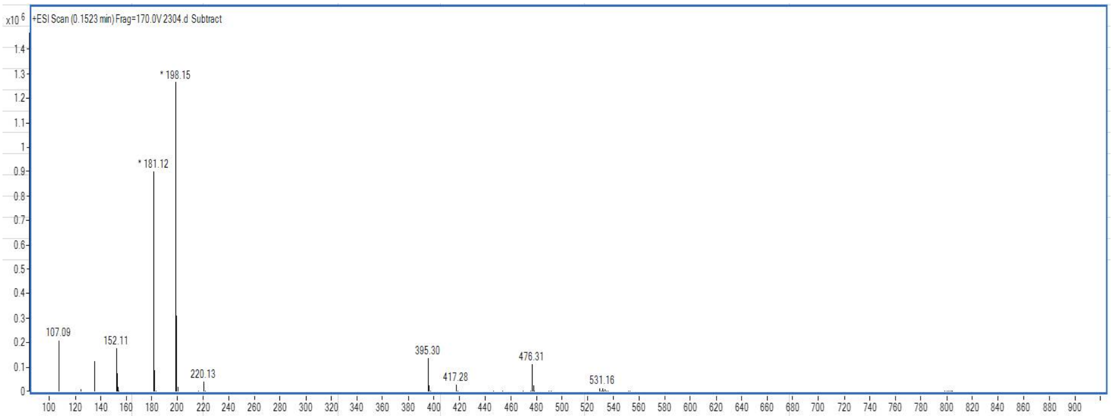

Figure S4-12. The ^{1}H-NMR Spectra of compound **13**

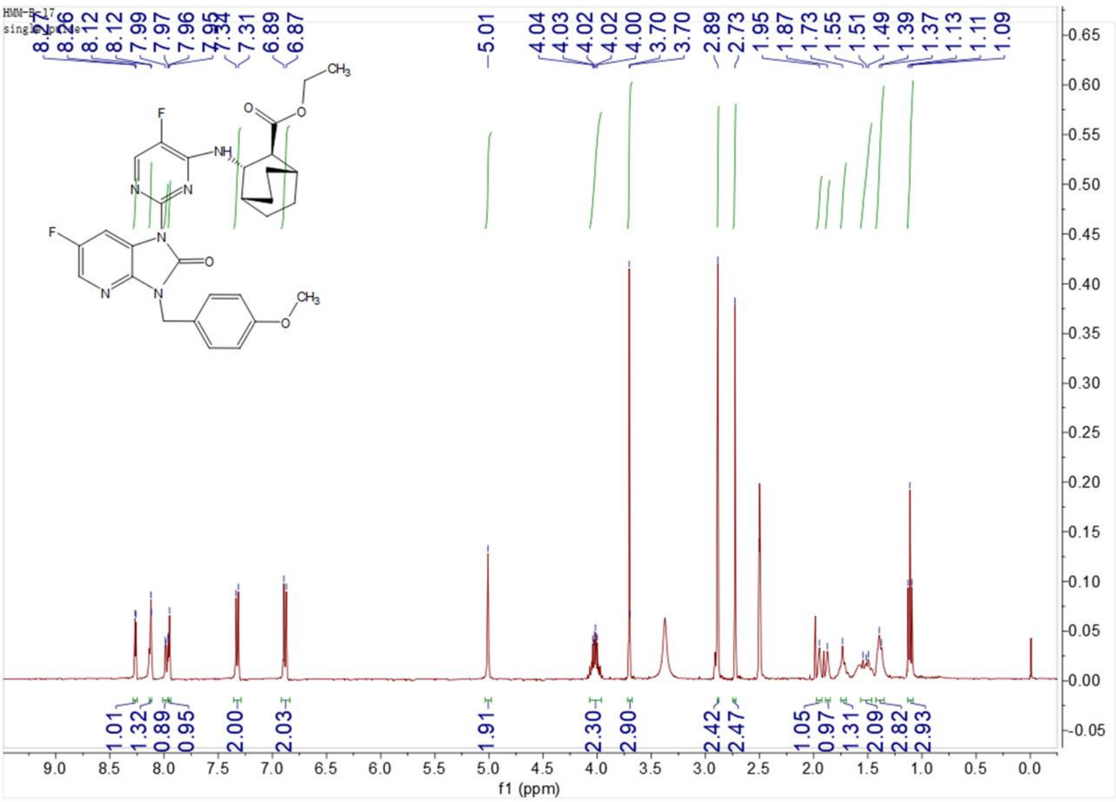

Figure S4-13. The ESI-MS Spectra of compound **13**

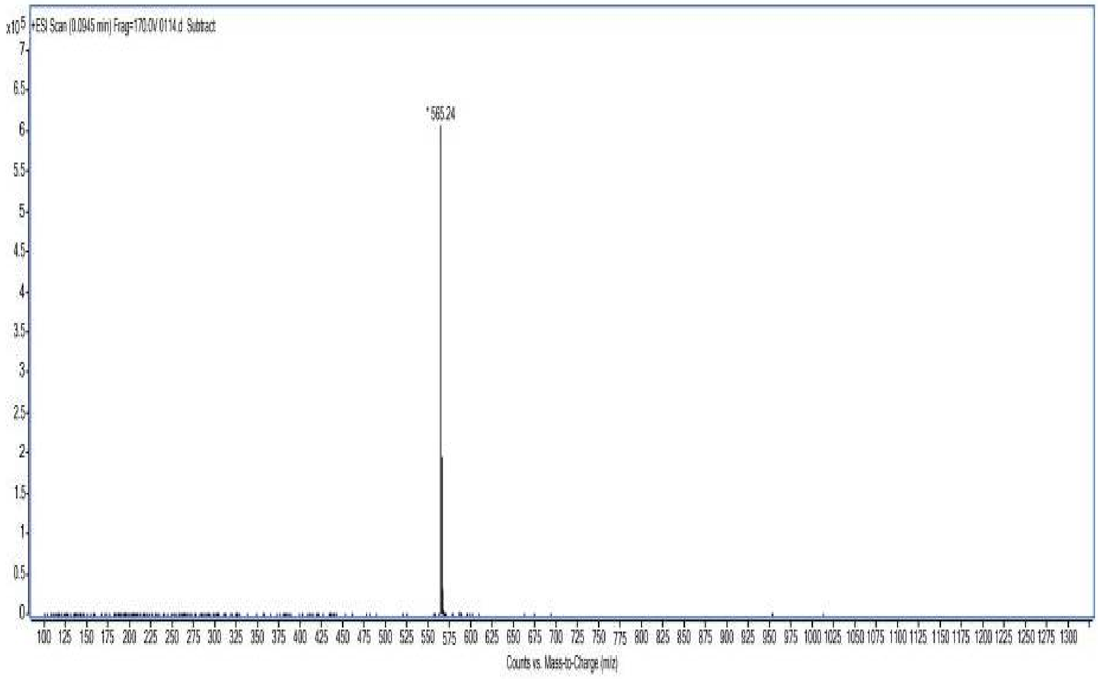

Figure S4-14. The ^{1}H-NMR Spectra of compound **15**

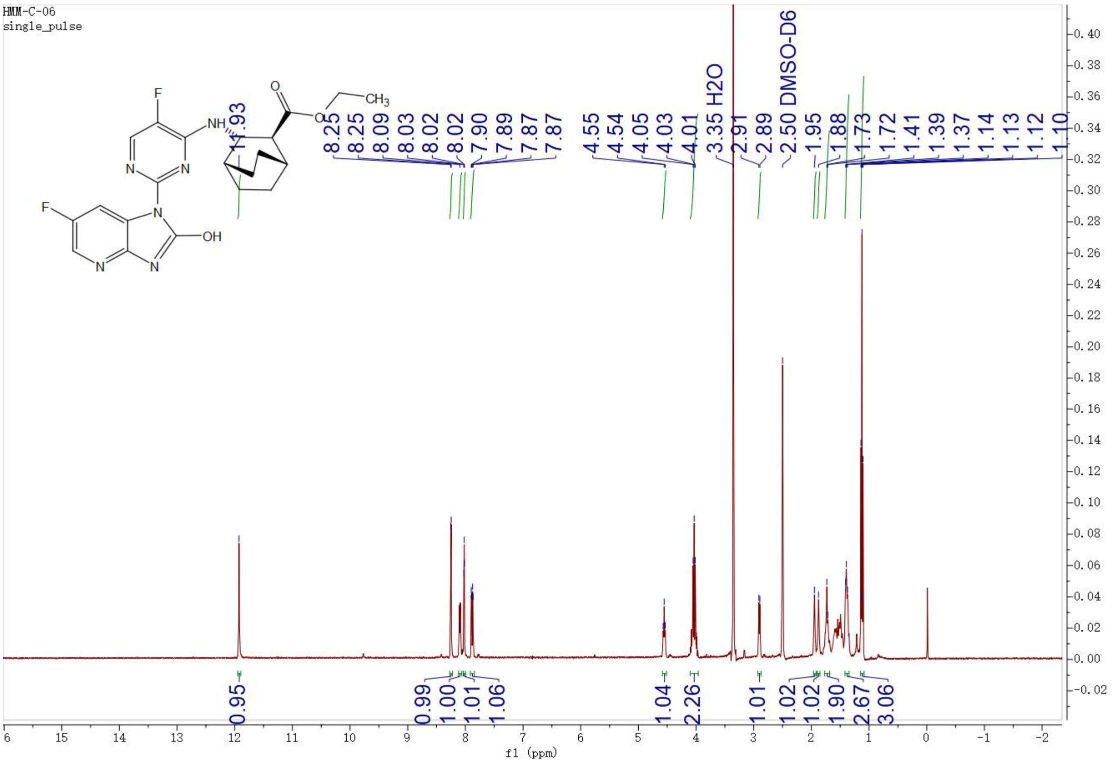

Figure S4-15. The ESI-MS Spectra of compound **15**

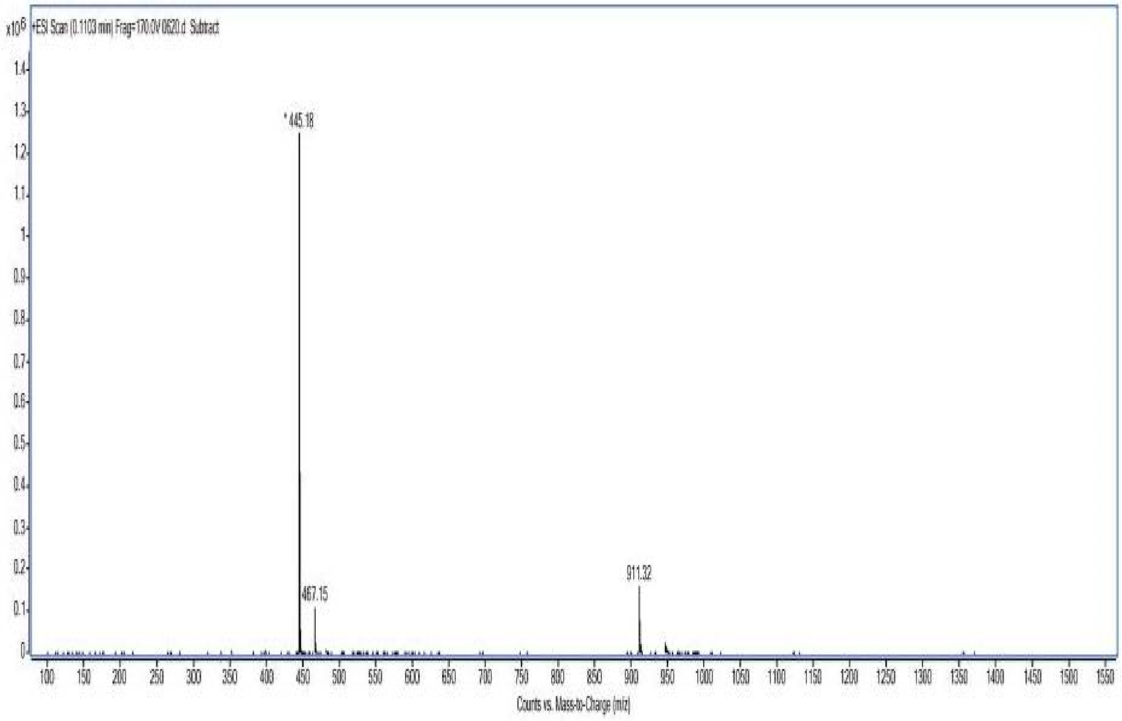

Figure S4-16. The ^{1}H-NMR Spectra of compound **16**

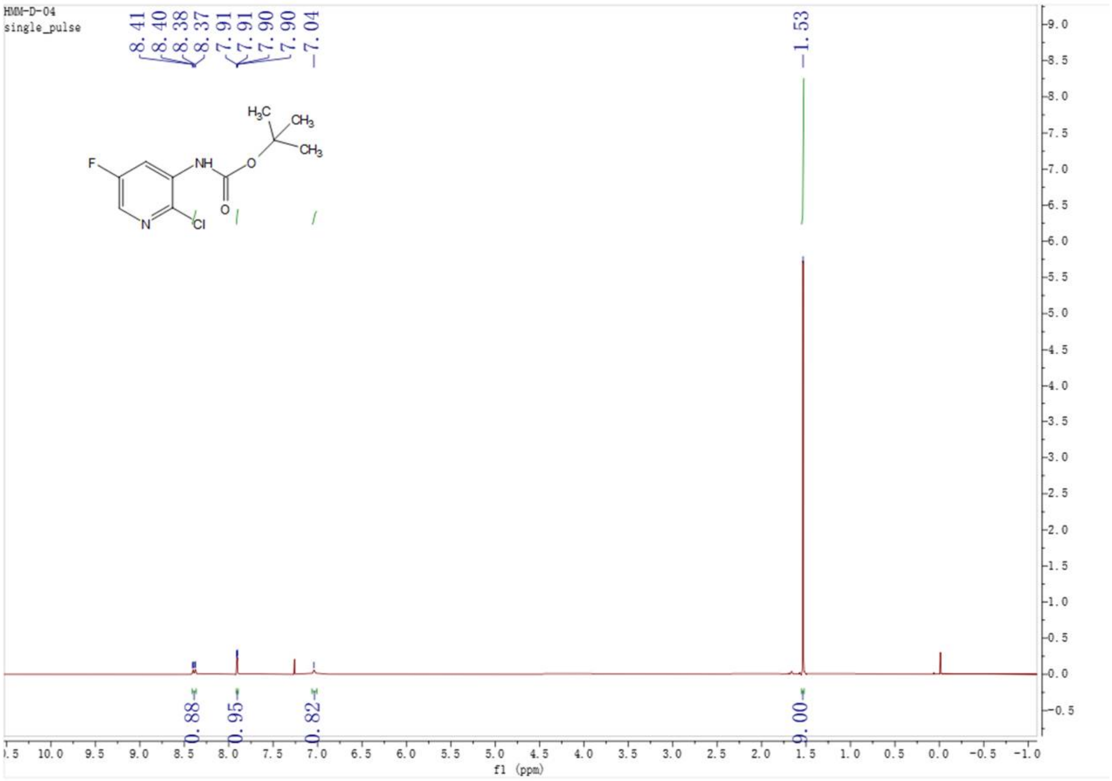

Figure S4-17. The ESI-MS Spectra of compound **16**

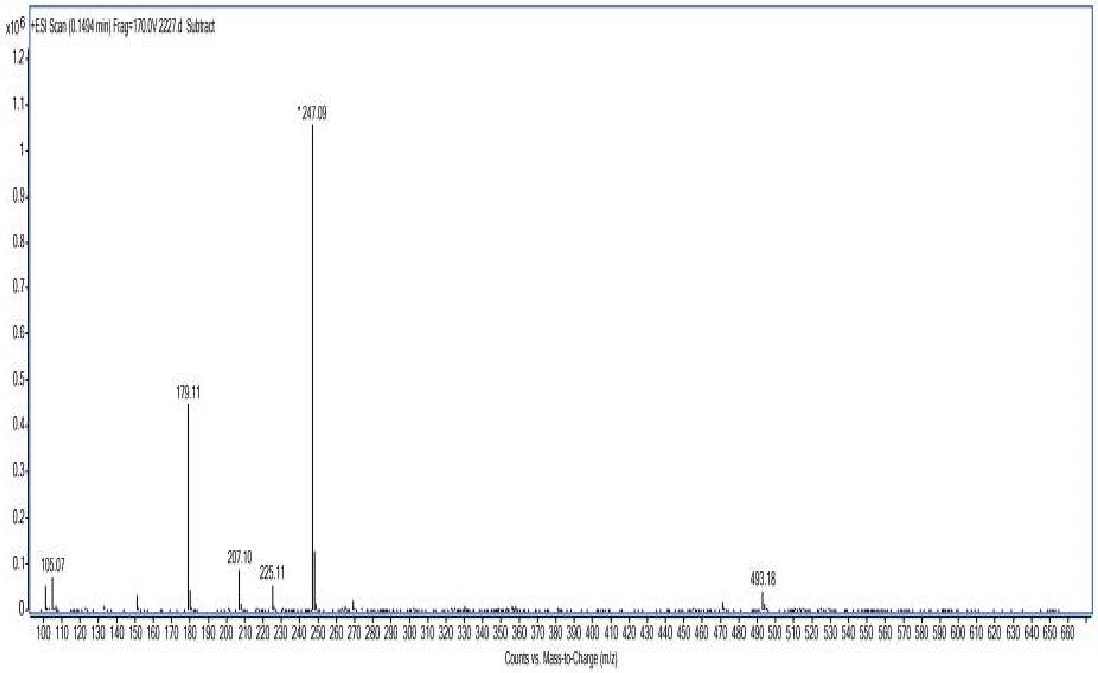

Figure S4-18. The ^{1}H-NMR Spectra of compound **17**

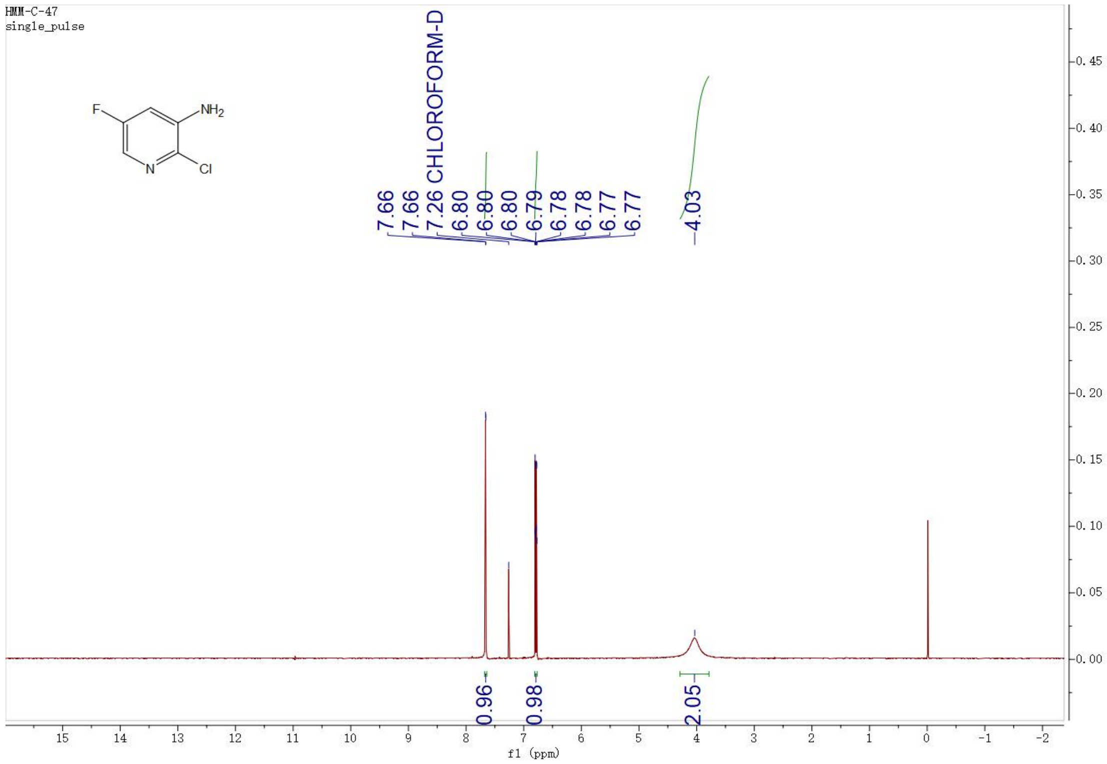

Figure S4-19. The ESI-MS Spectra of compound 17

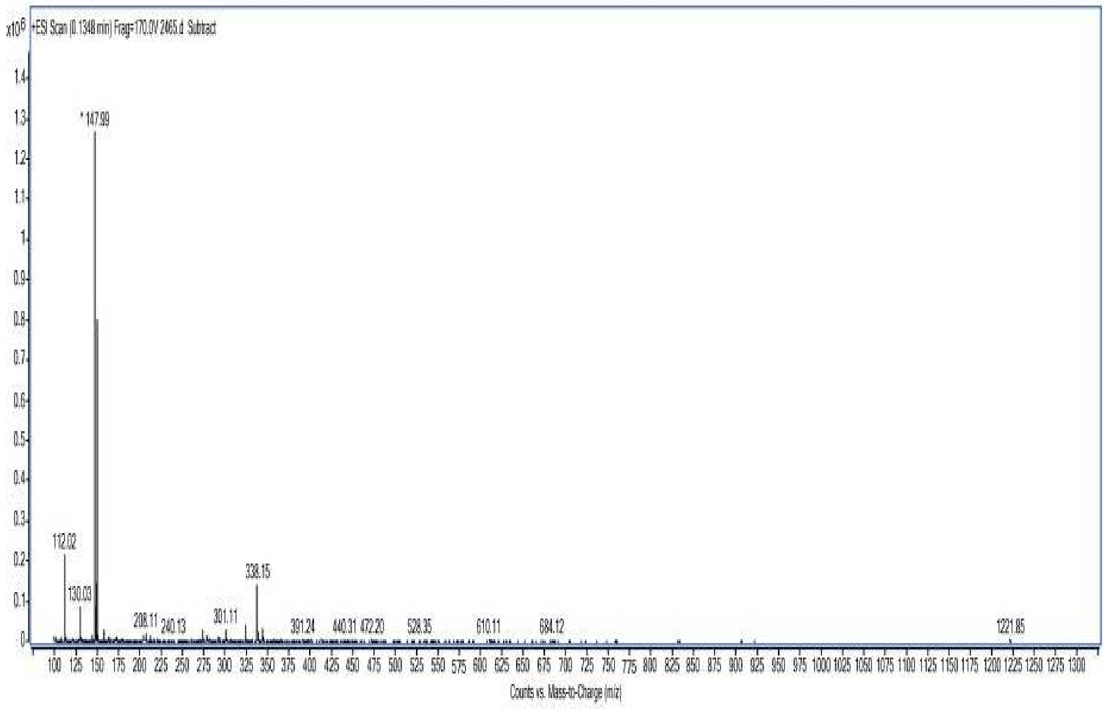

Figure S4-20. The ^{1}H-NMR Spectra of compound **18**

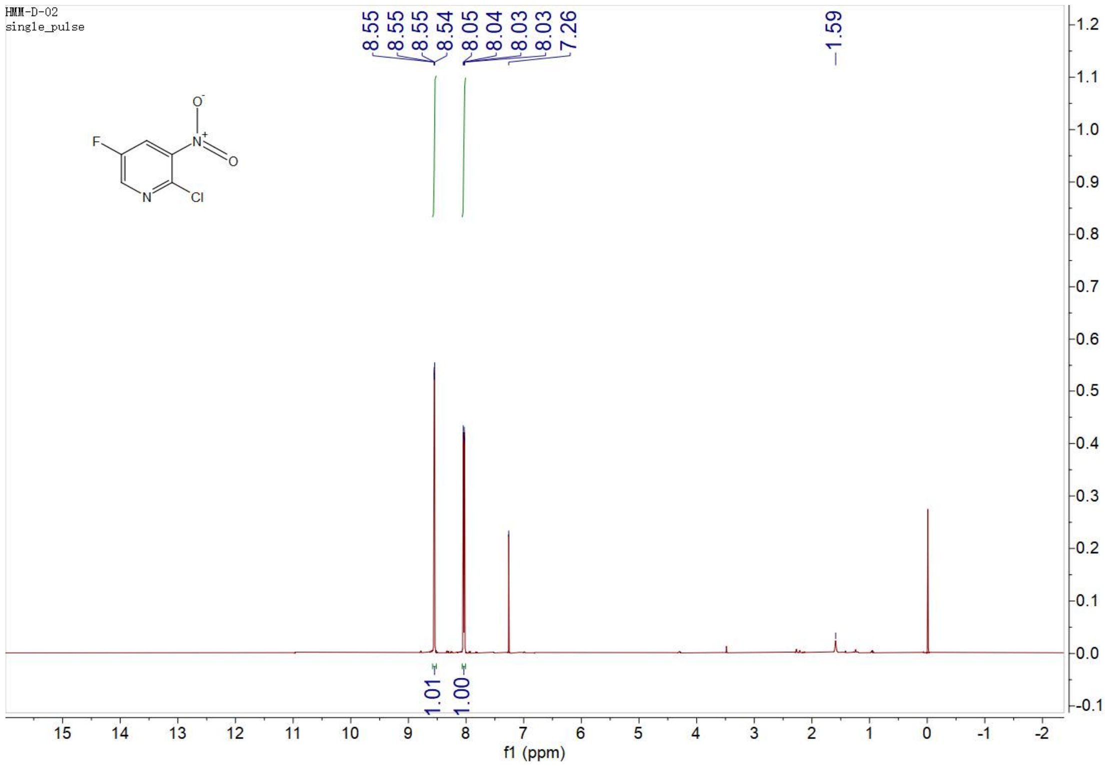

Figure S4-21. The ESI-MS Spectra of compound **18**

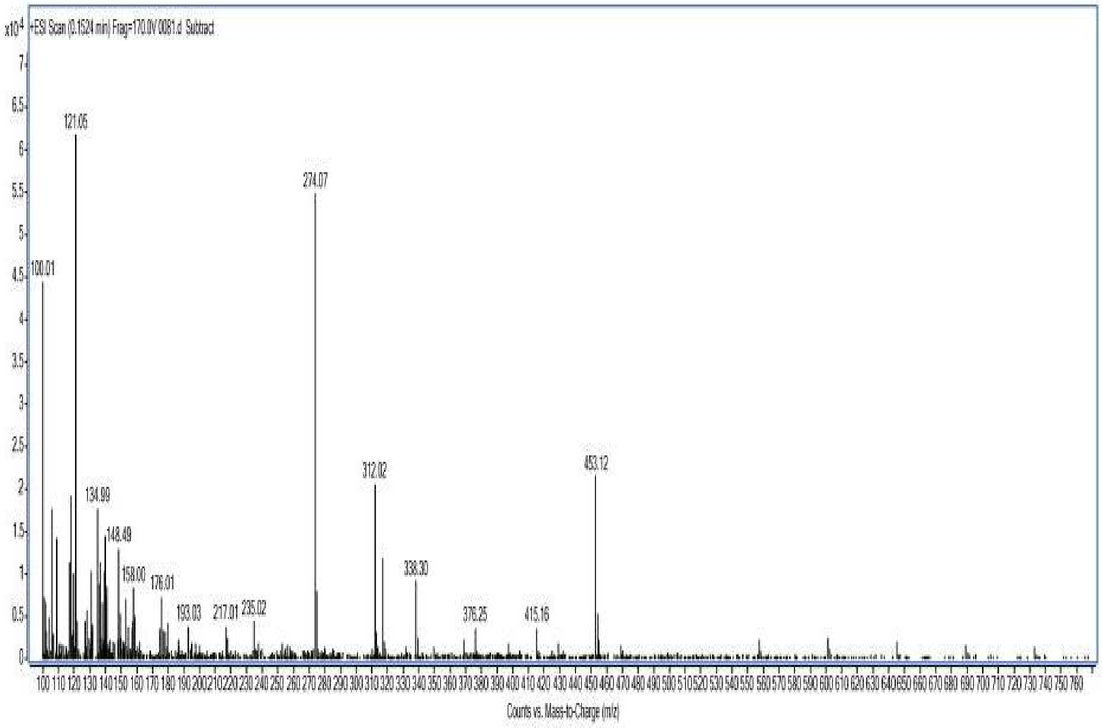

Figure S4-22. The ^{1}H-NMR Spectra of compound **20**

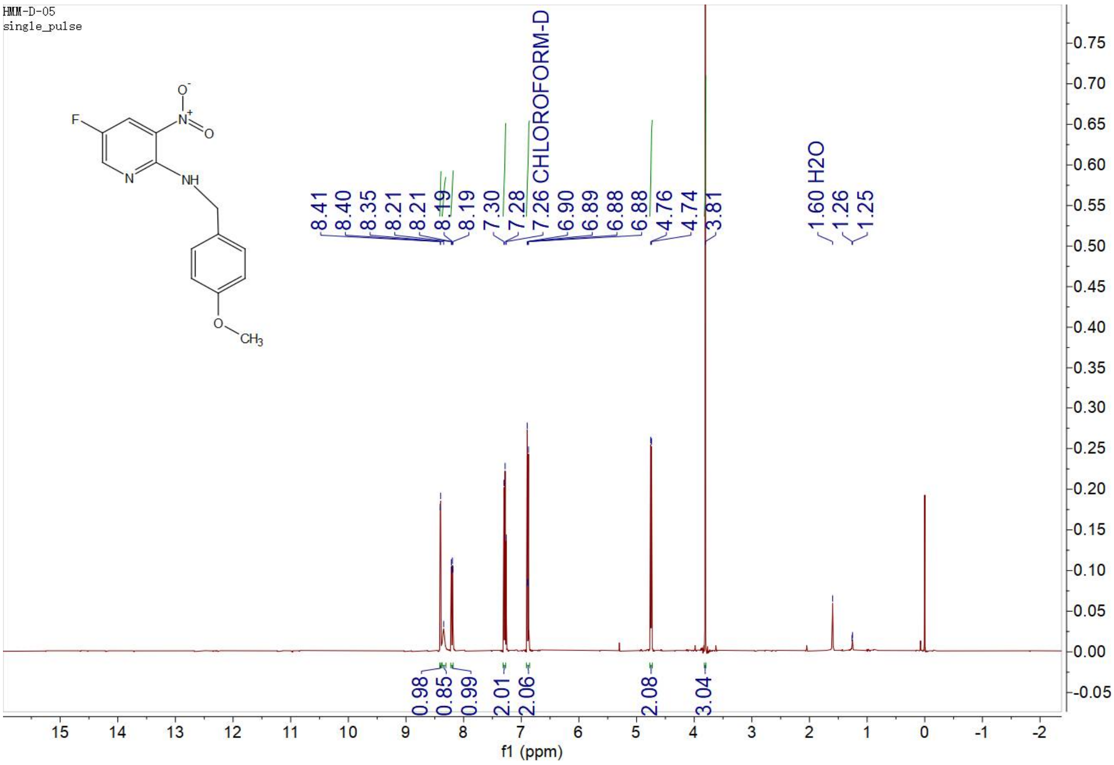

Figure S4-23. The ESI-MS Spectra of compound **20**

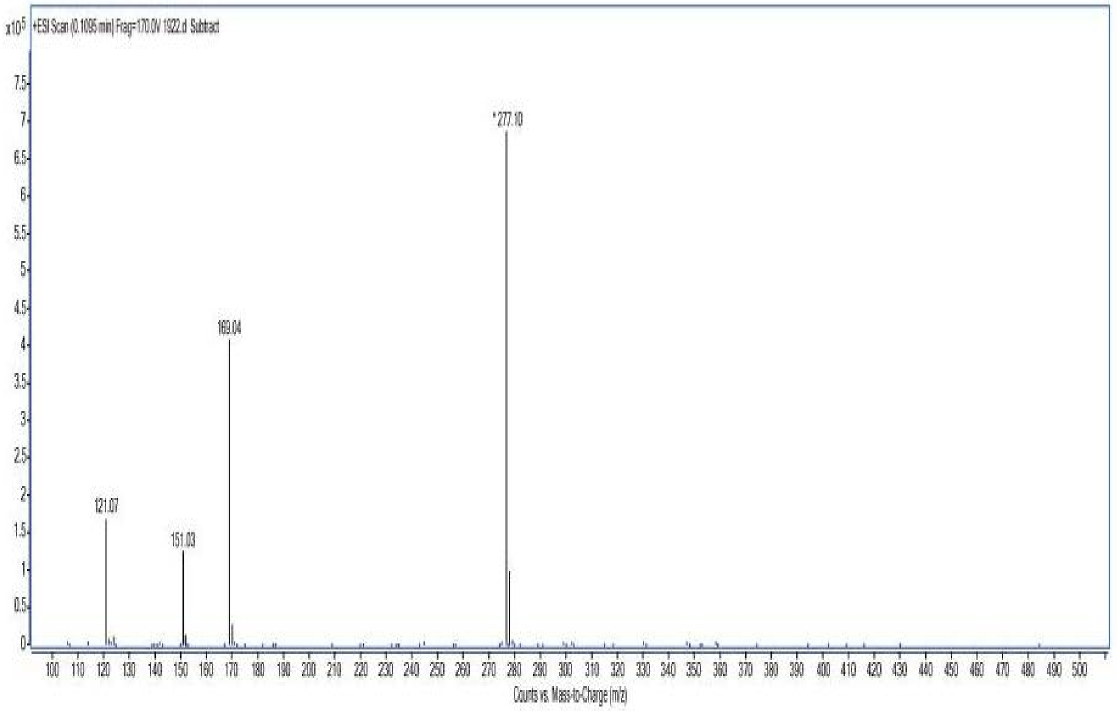

Figure S4-24. The ^{1}H-NMR Spectra of compound **21**

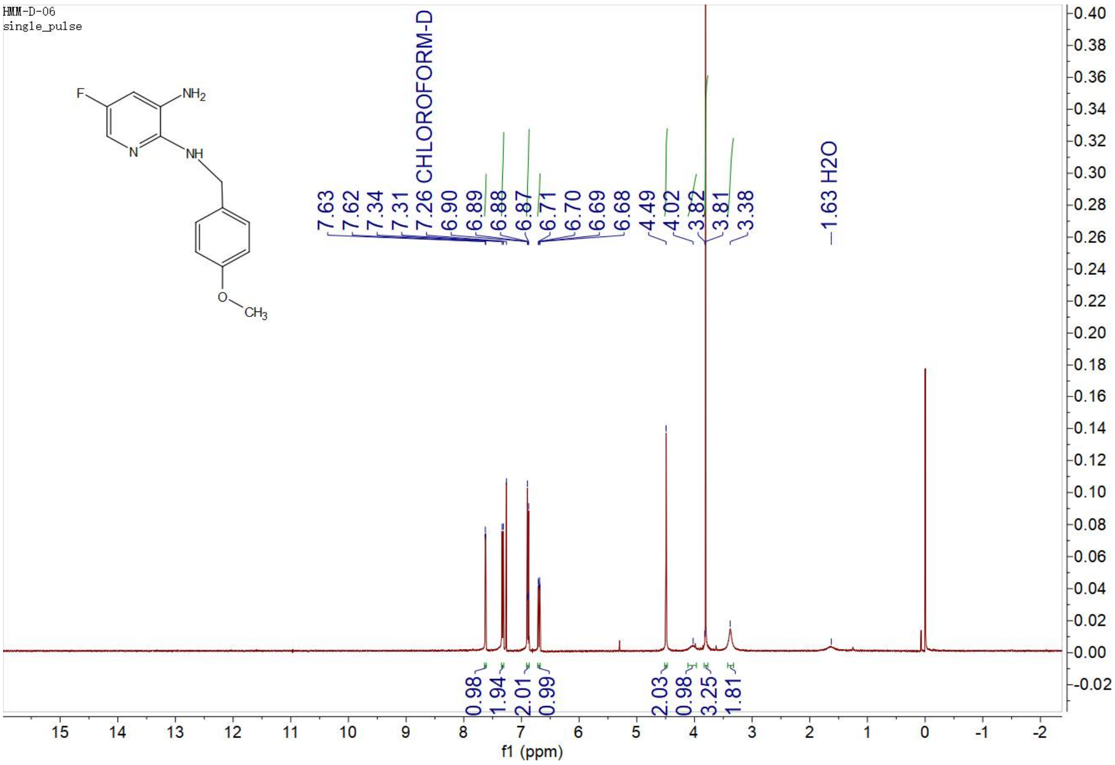

Figure S4-25. The ESI-MS Spectra of compound **21**

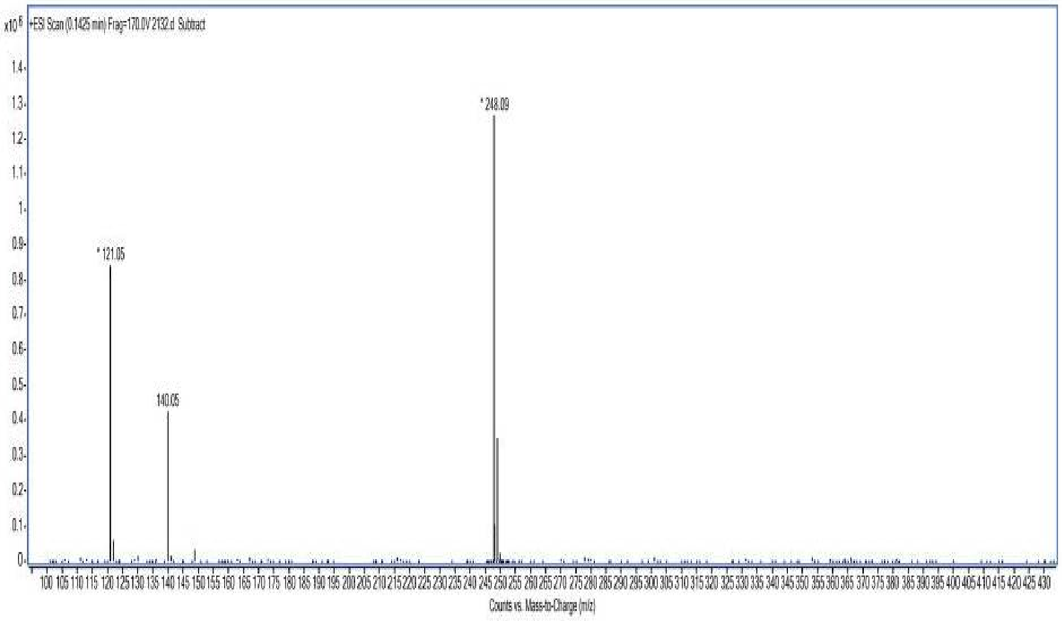

Figure S4-26. The ^{1}H-NMR Spectra of compound **23**

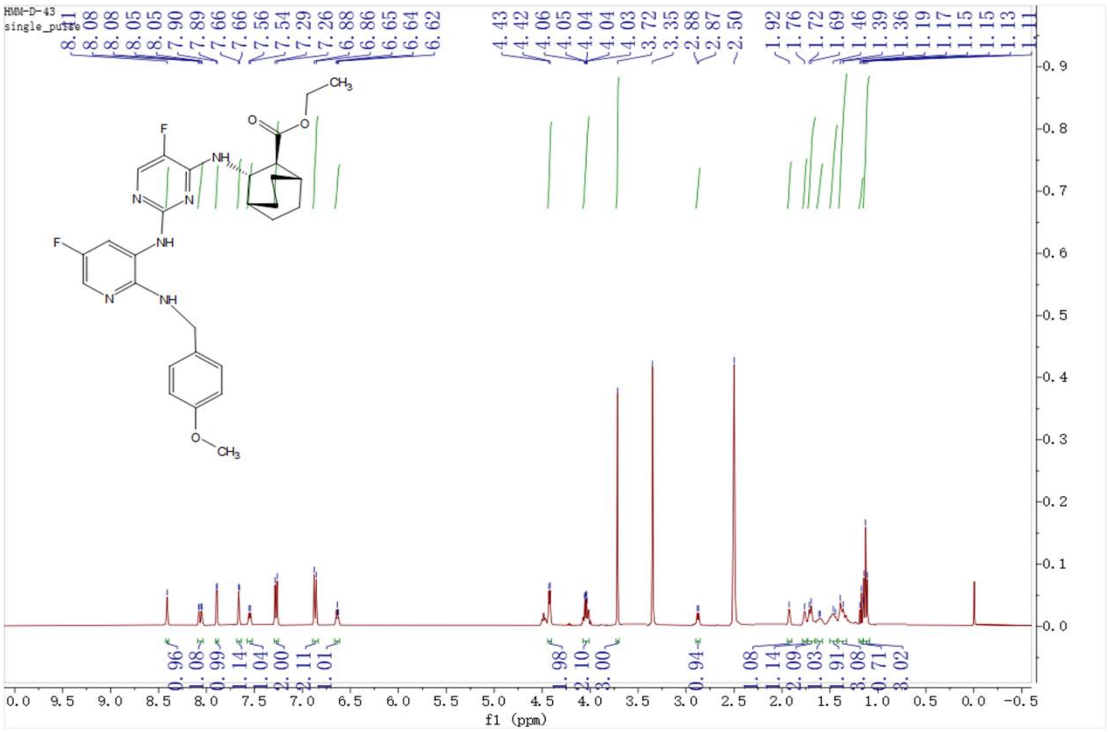

Figure S4-27. The ESI-MS Spectra of compound **23**

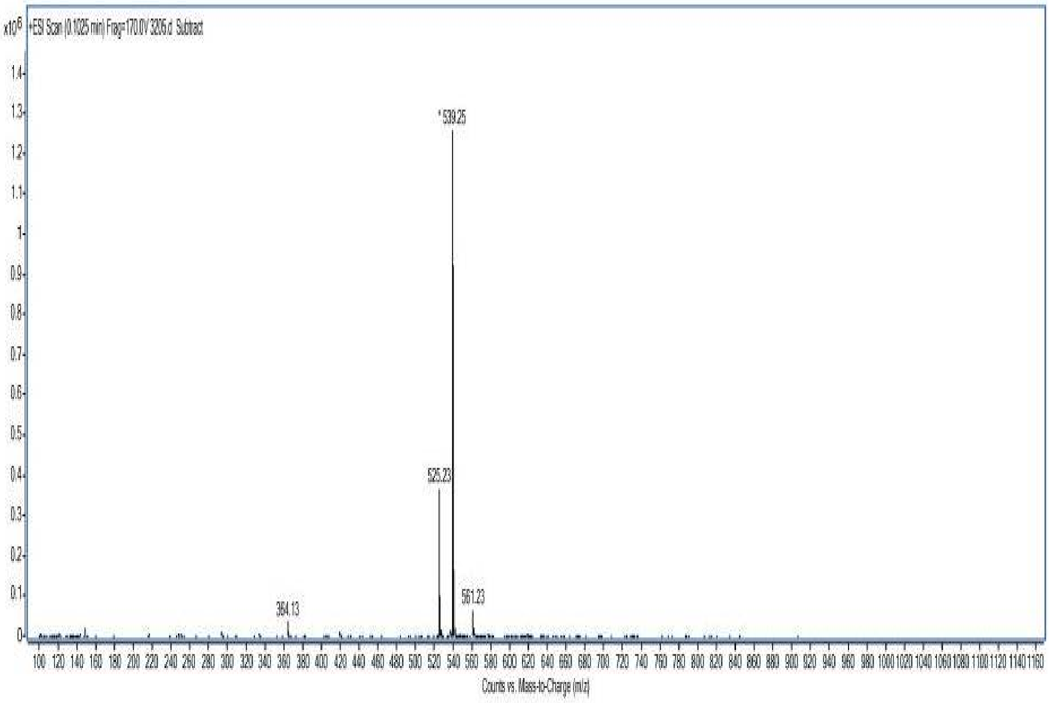

Figure S4-28. The ^{1}H-NMR Spectra of compound **24**

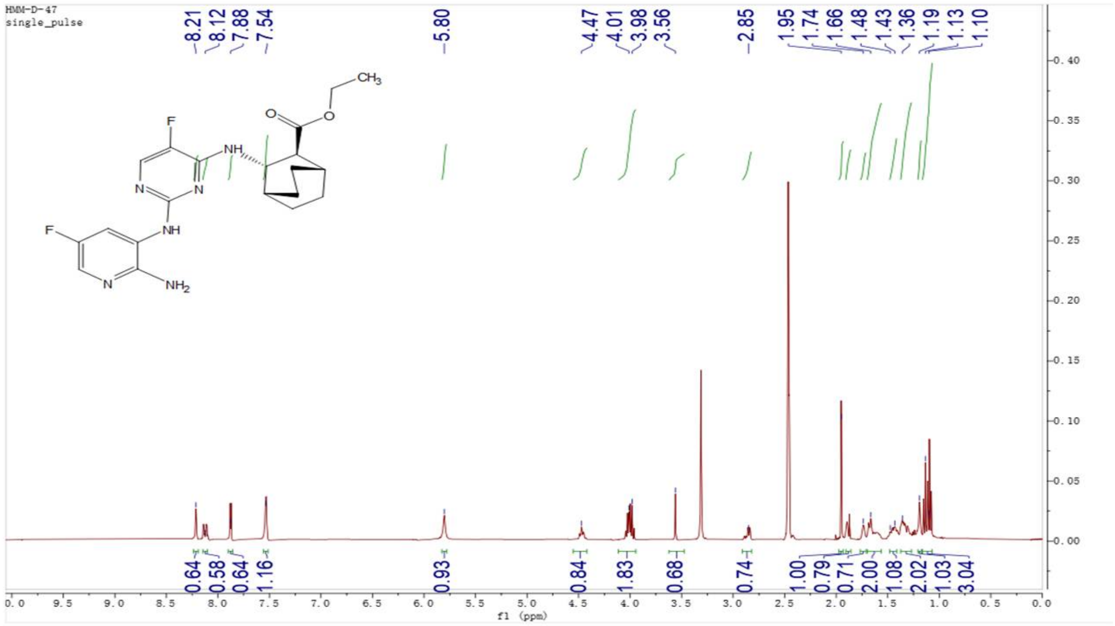

Figure S4-29. The ESI-MS Spectra of compound **24**

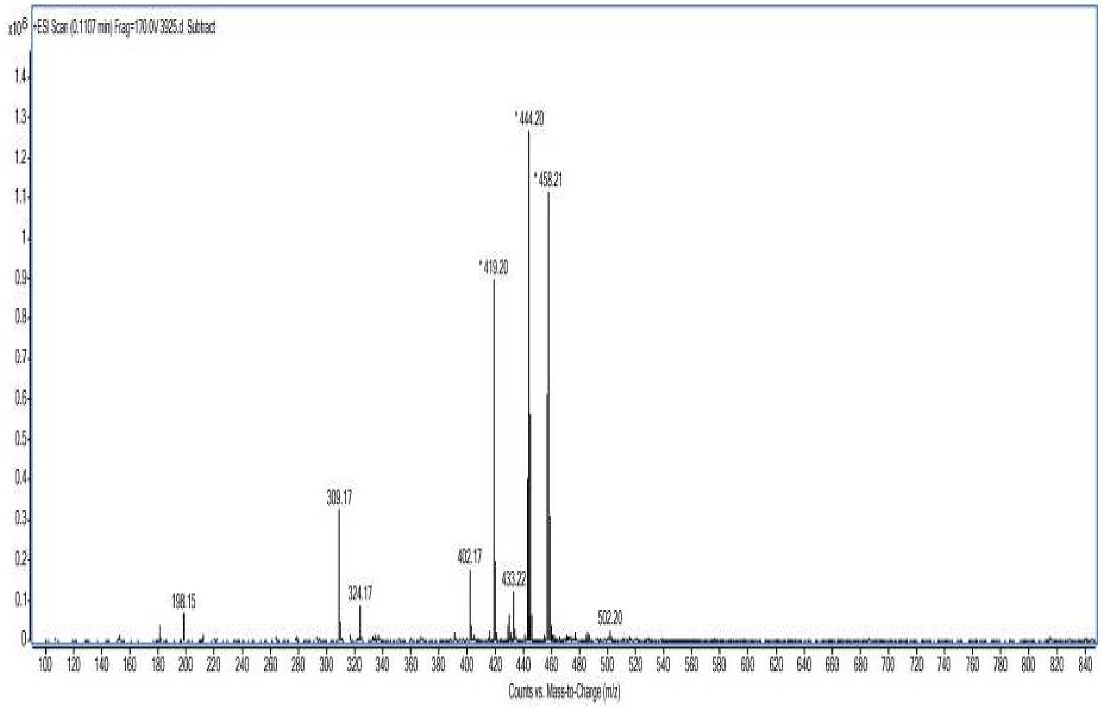

Figure S4-30. The ^{1}H-NMR Spectra of compound **25**

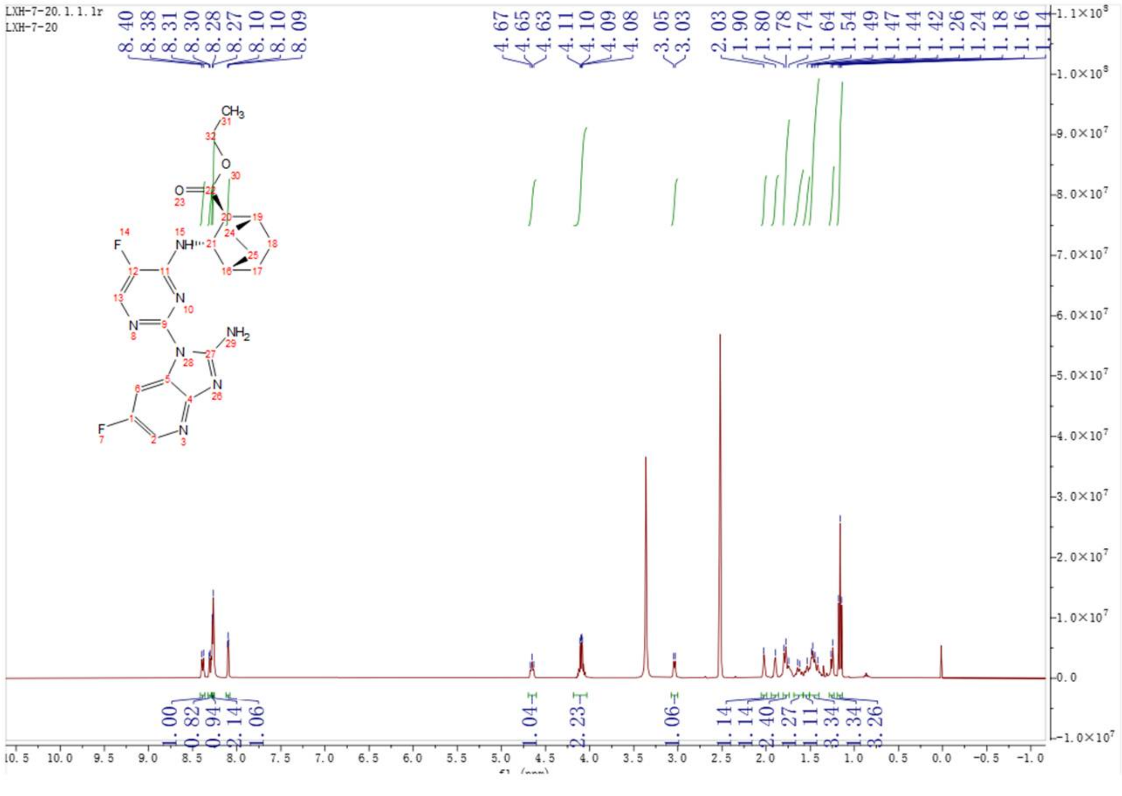

Figure S4-31. The ^{1}H-NMR Spectra of compound **25** (D_2O exchangeable)

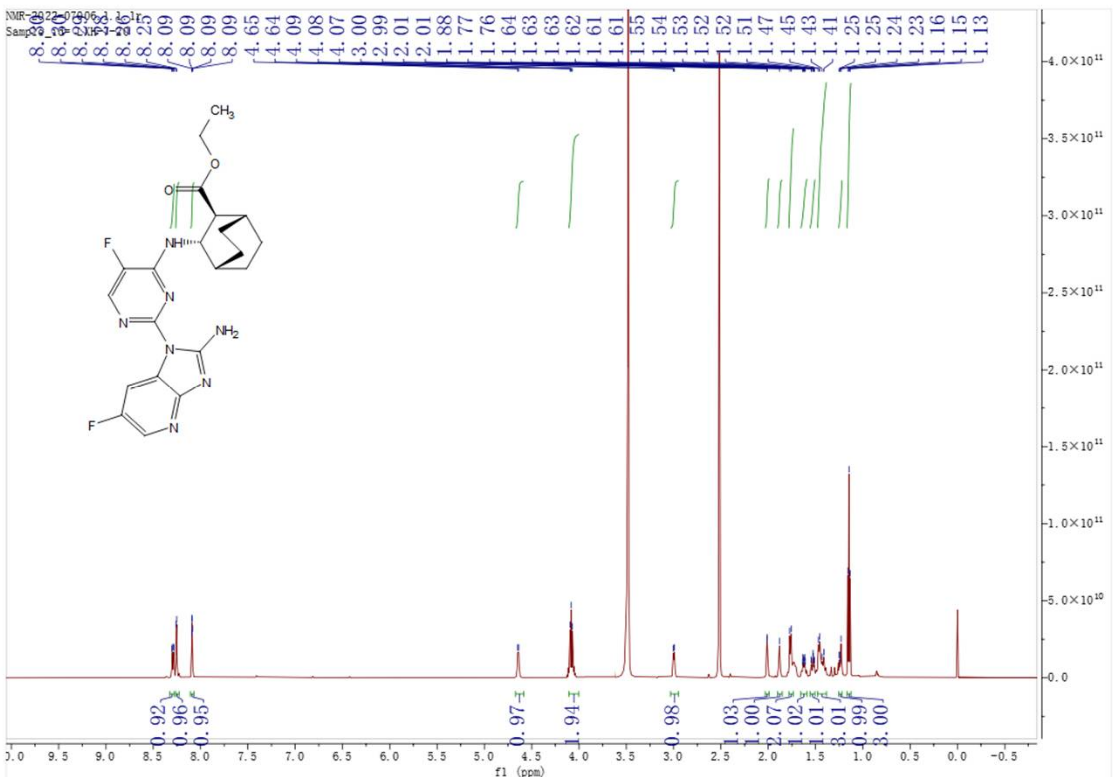

Figure S4-32. The ESI-MS Spectra of compound **25**

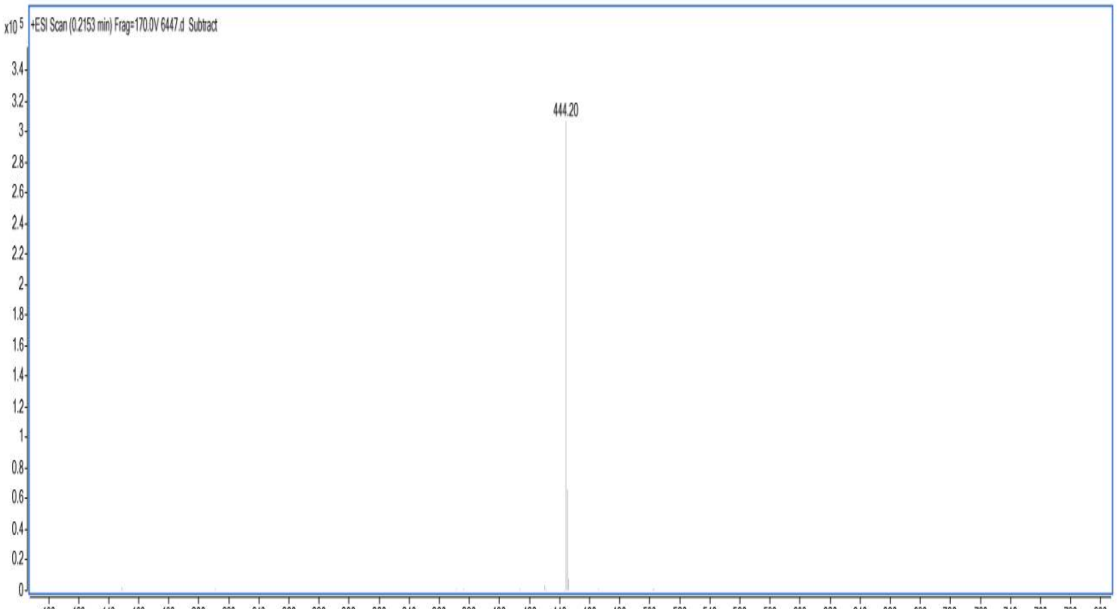

Figure S4-33. The ^{1}H-NMR Spectra of compound **I**

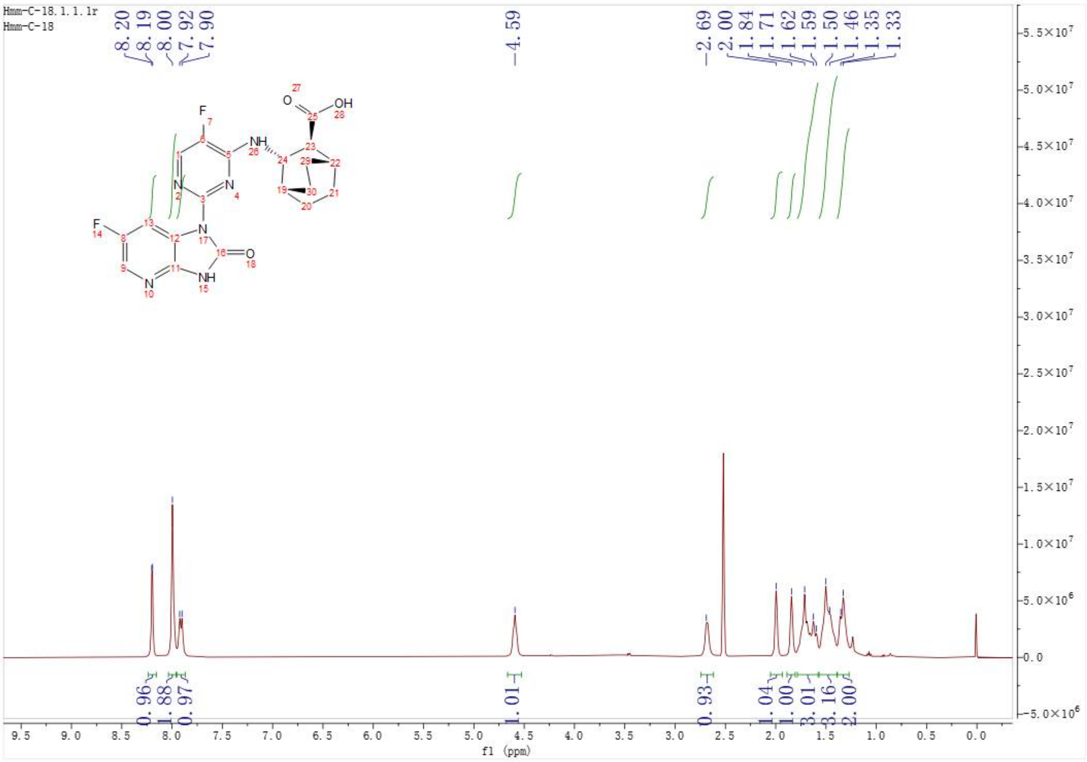

Figure S4-34 The ^{1}H-NMR Spectra of compound **I** (D_2O exchangeable)

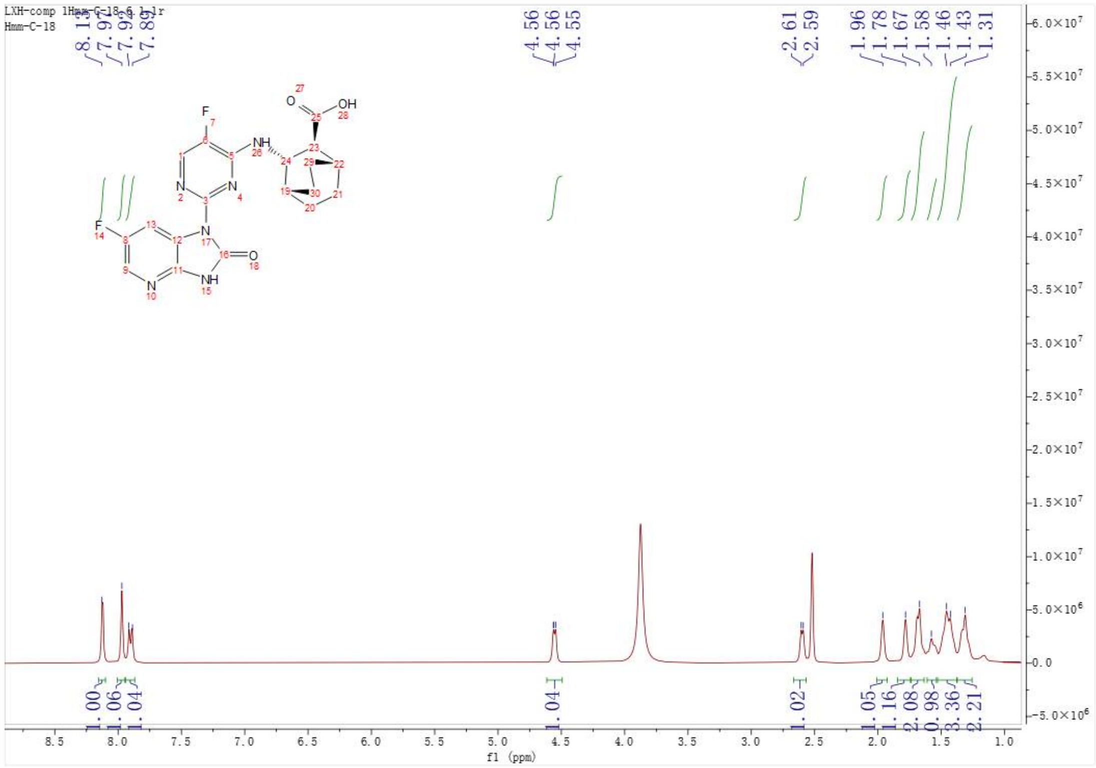

Figure S4-35. The ^{13}C-NMR Spectra of compound **I**

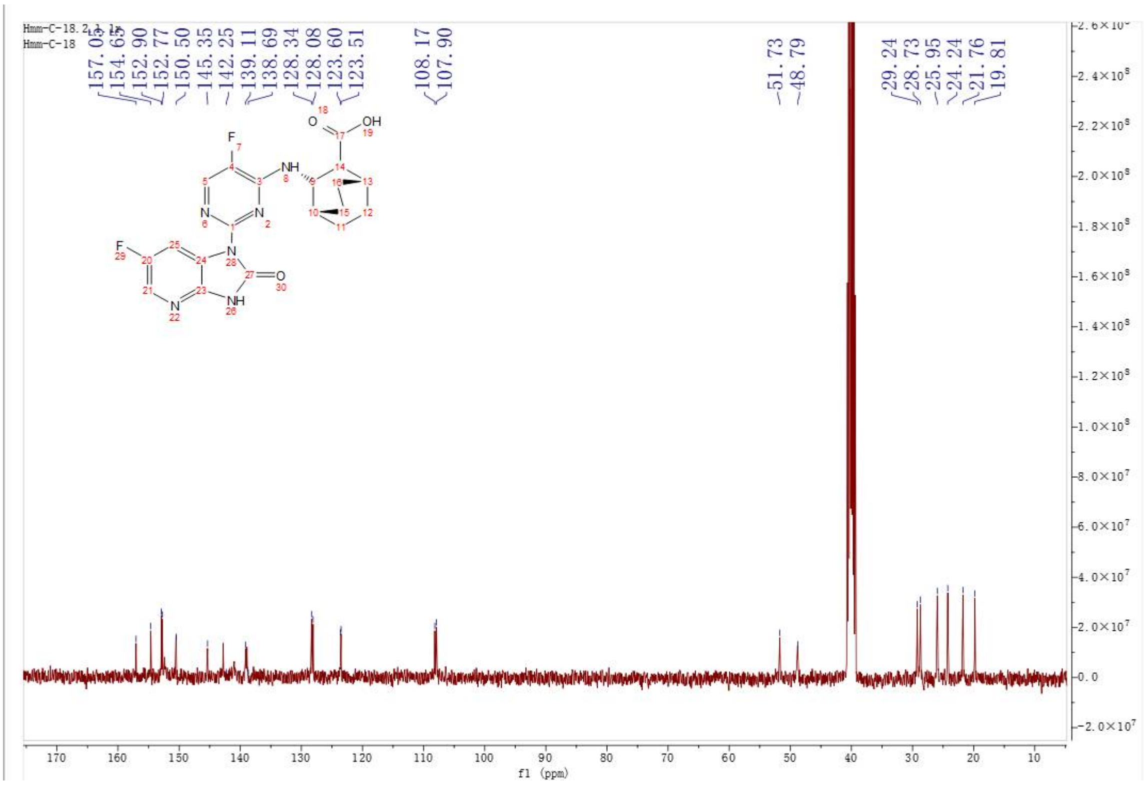

Figure S4-36. The ^{19}F-NMR Spectra of compound **I**

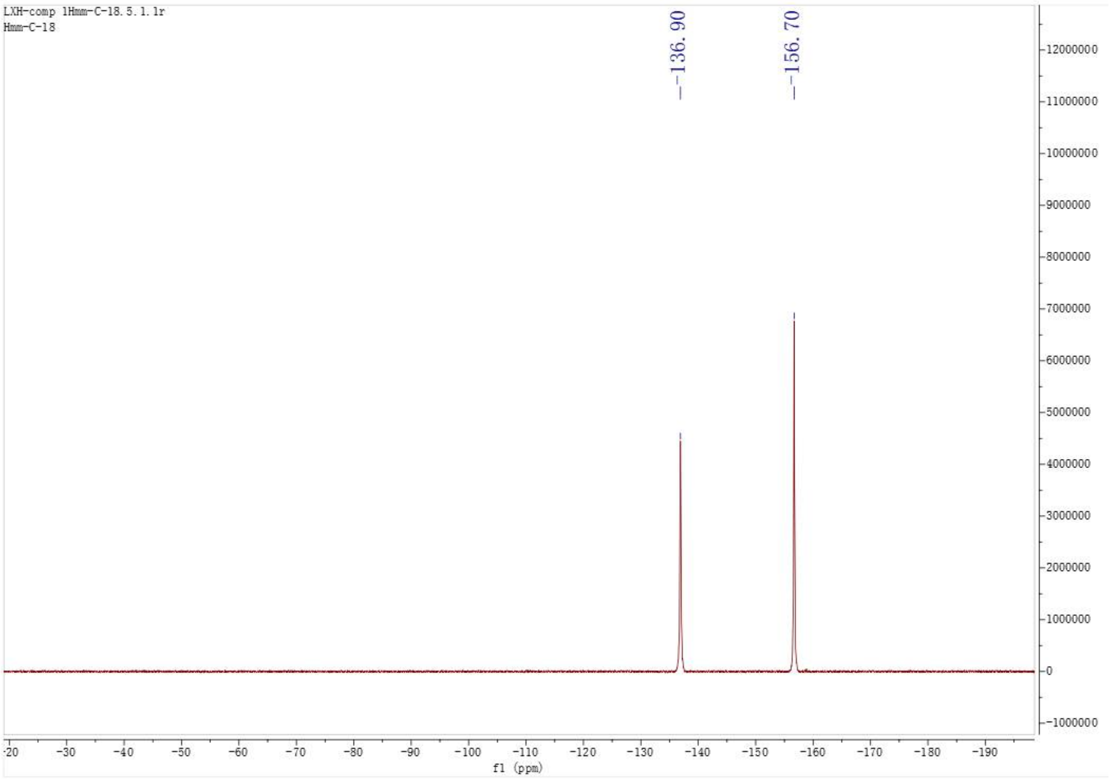

Figure S4-37. The ESI-MS Spectra of compound **I**

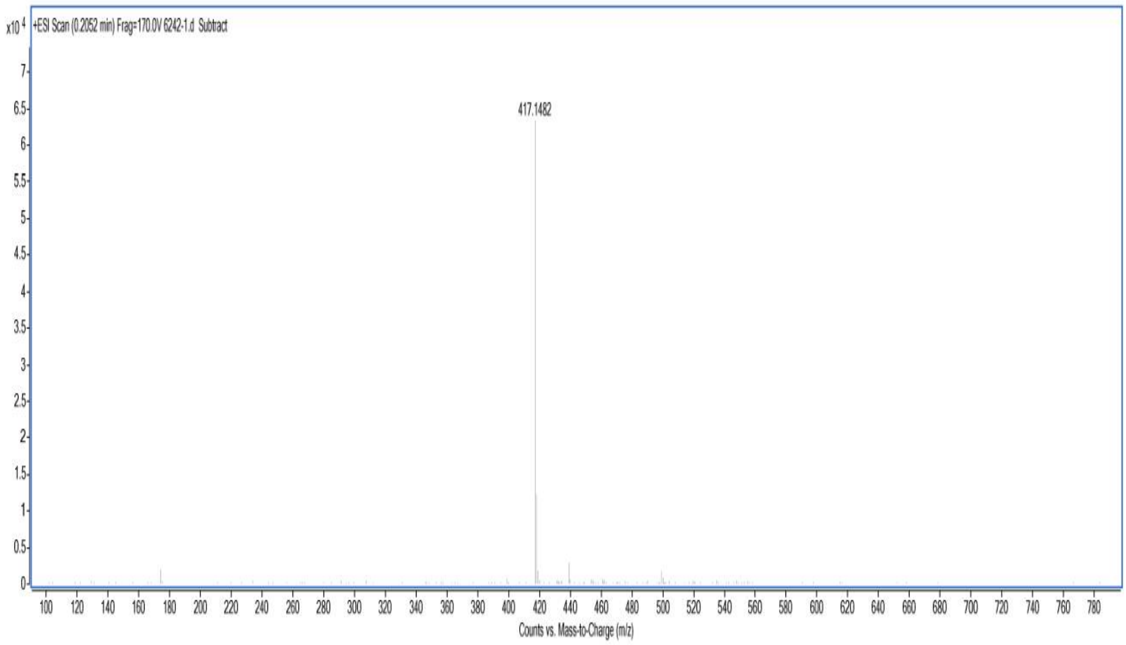

Figure S4-38. The HSQC Spectra of compound **I**

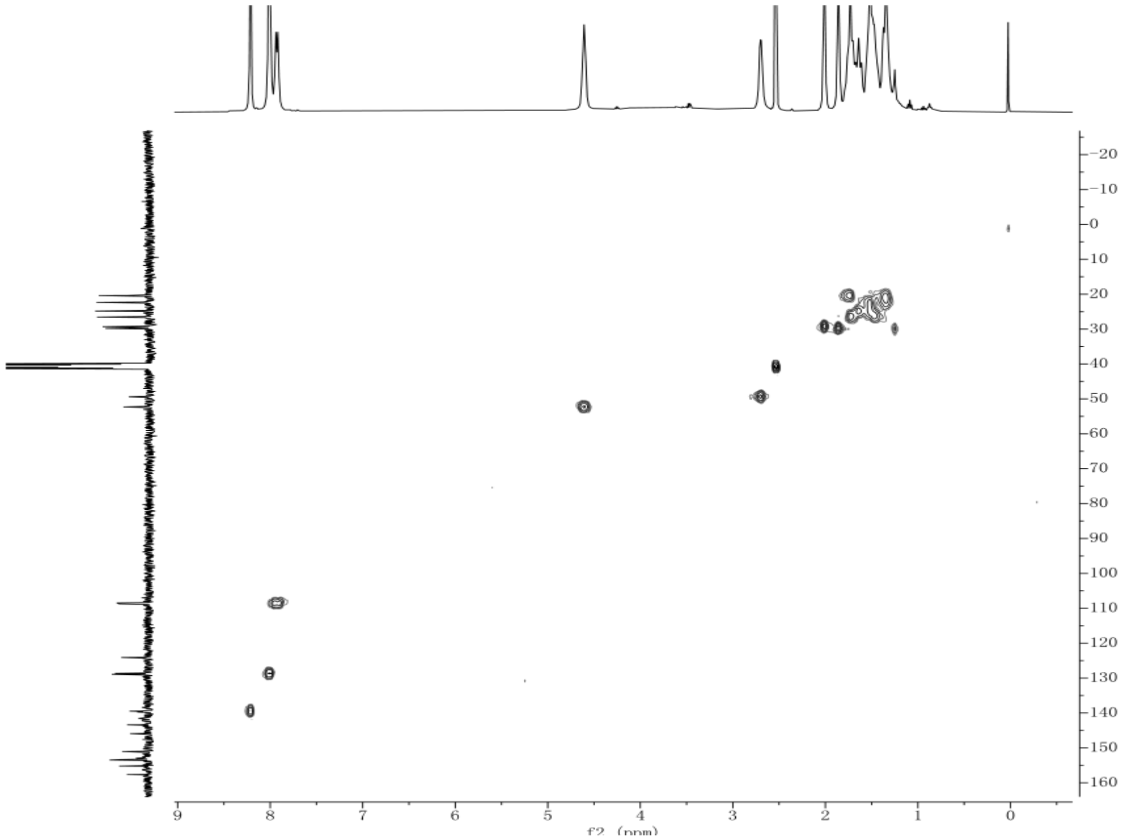

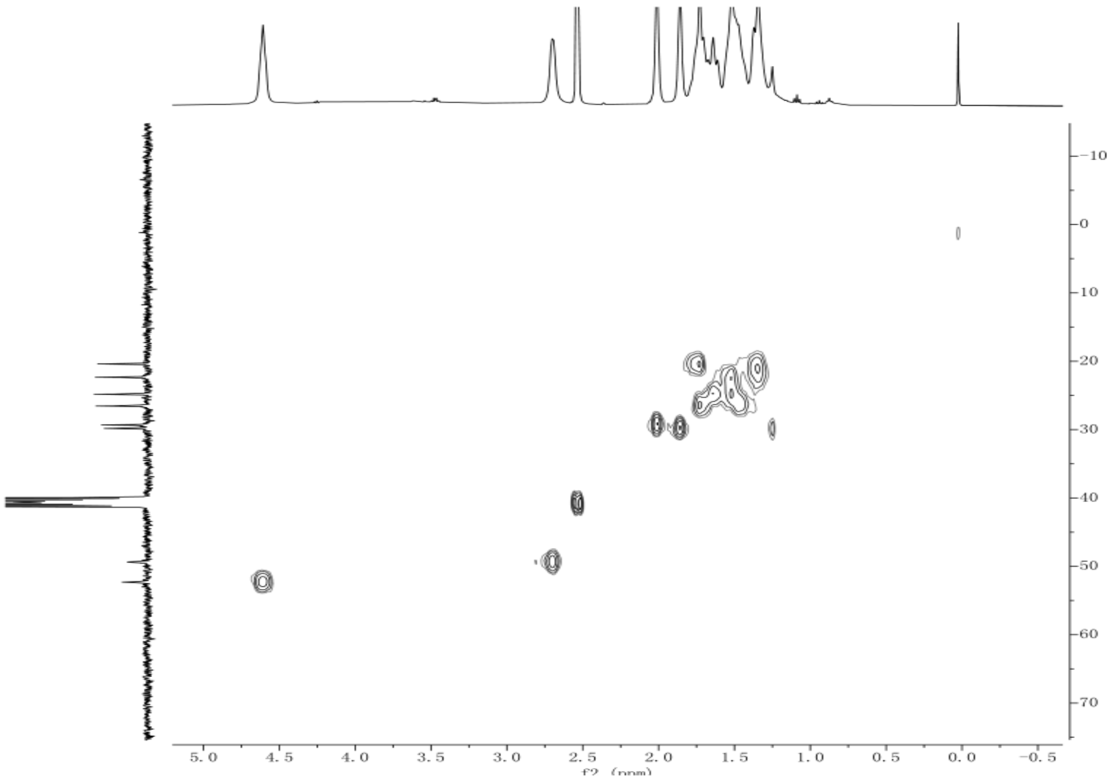

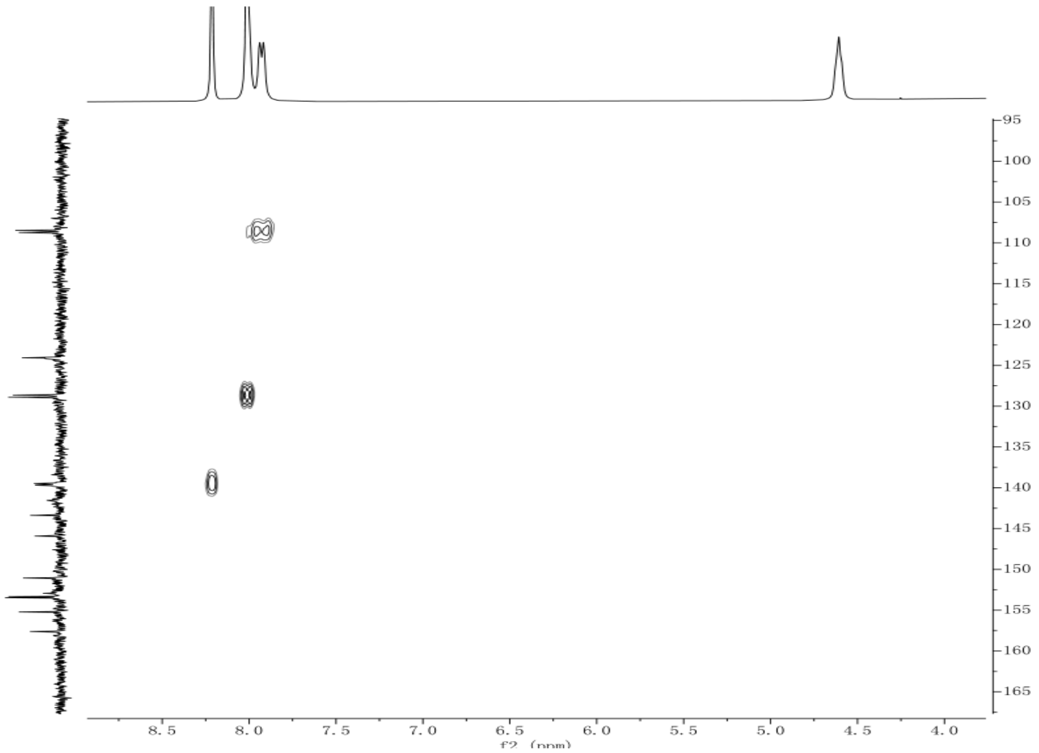

Figure S4-39. The ^{1}H-NMR Spectra of compound **II**

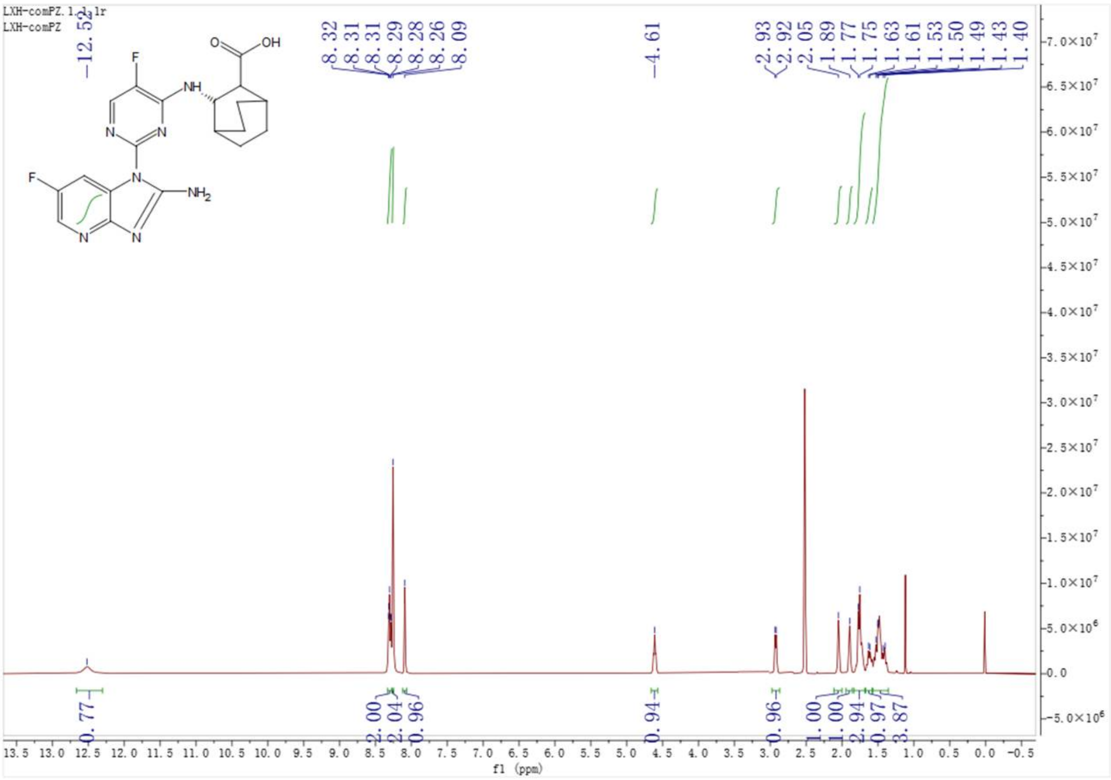

Figure S4-40. The ^{1}H-NMR Spectra of compound **II** (D_2O exchangeable)

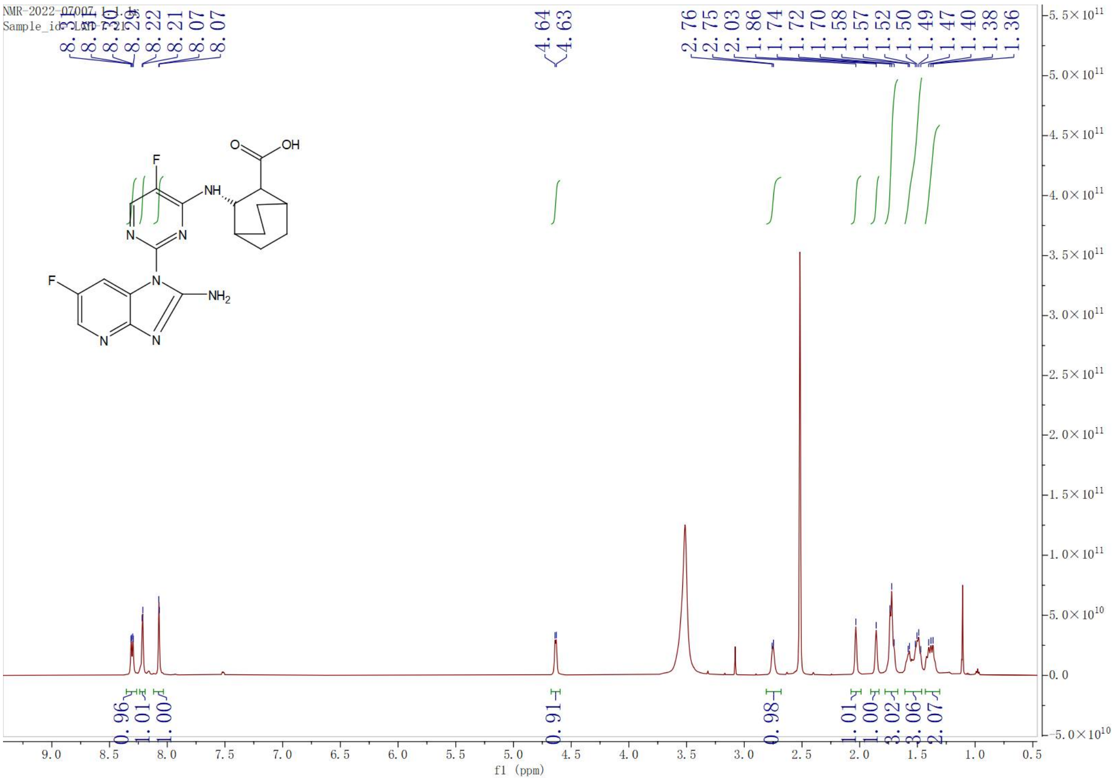

Figure S4-41. The ^{13}C-NMR Spectra of compound **II**

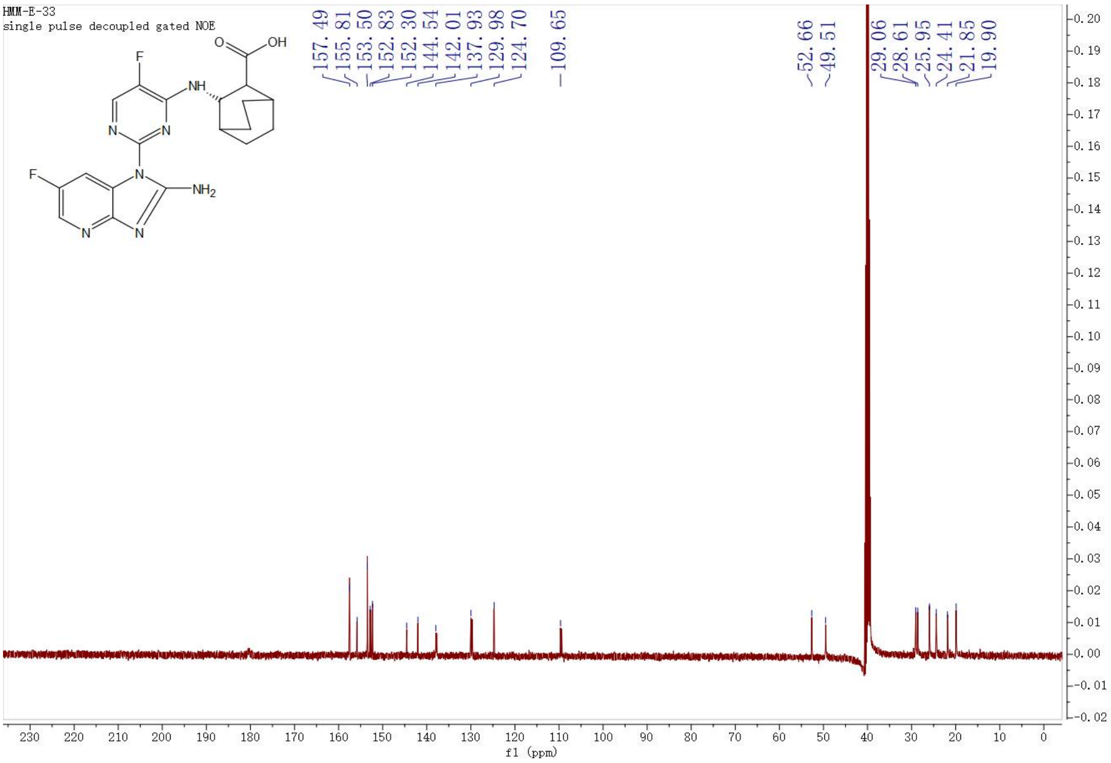

Figure S4-42. The ^{19}F-NMR Spectra of compound **II**

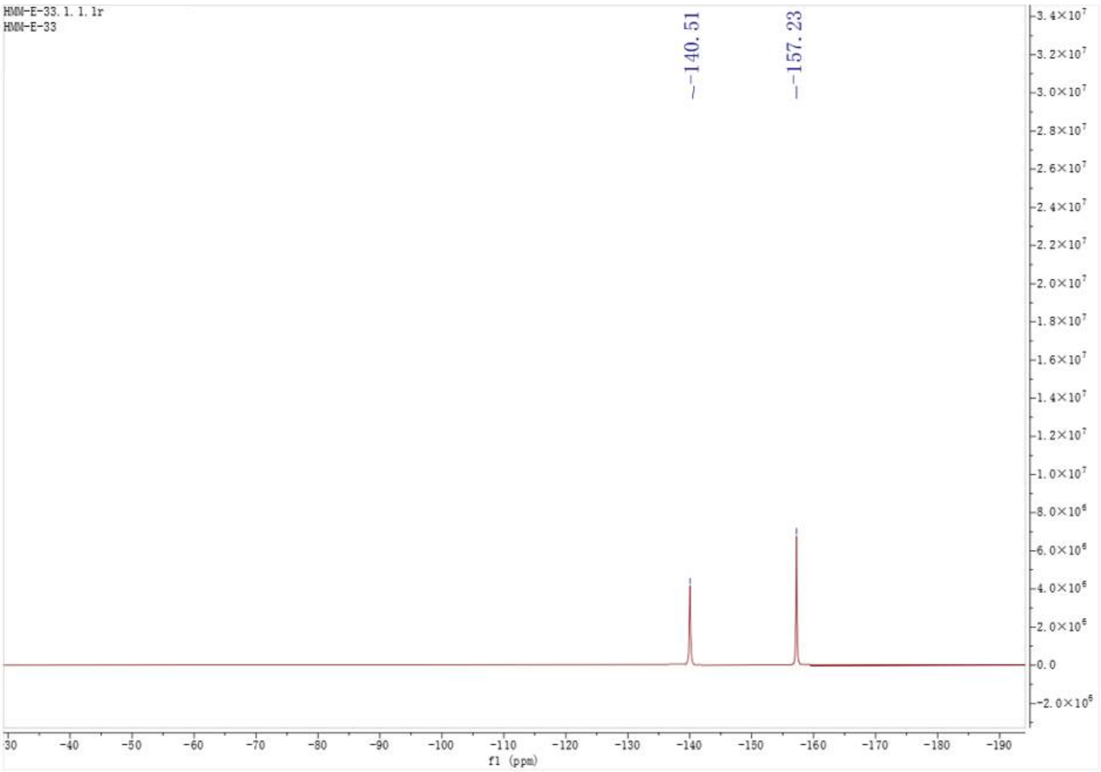

Figure S4-43. The ESI-MS Spectra of compound **II**

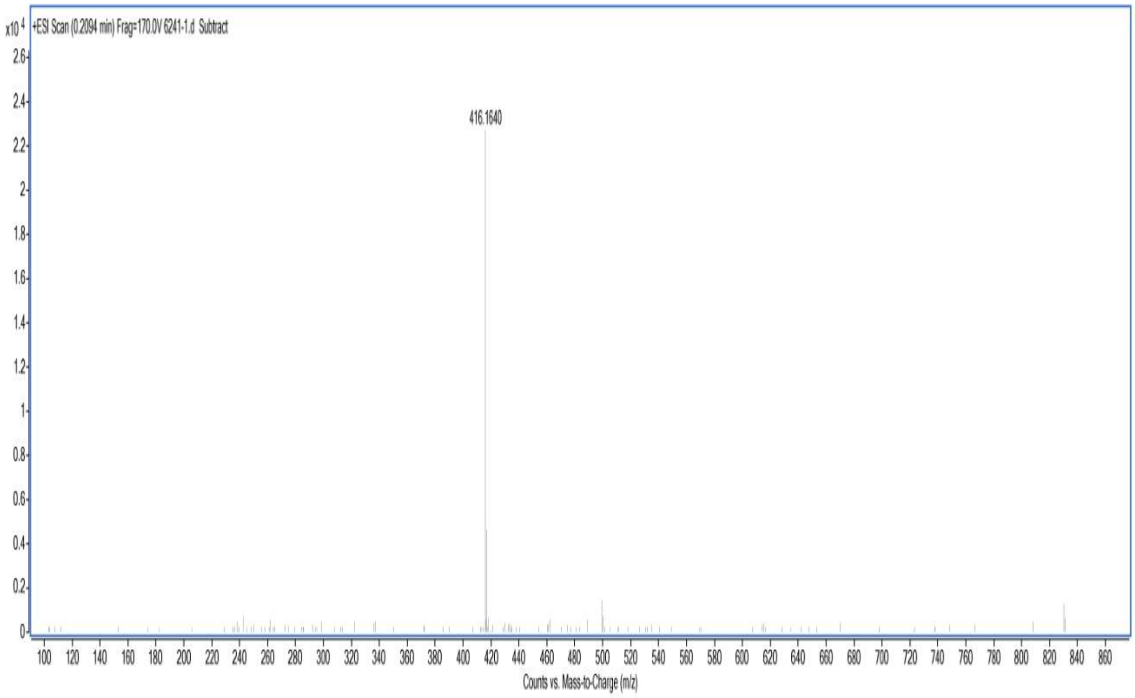

Figure S4-44. The HSQC Spectra of compound **II**

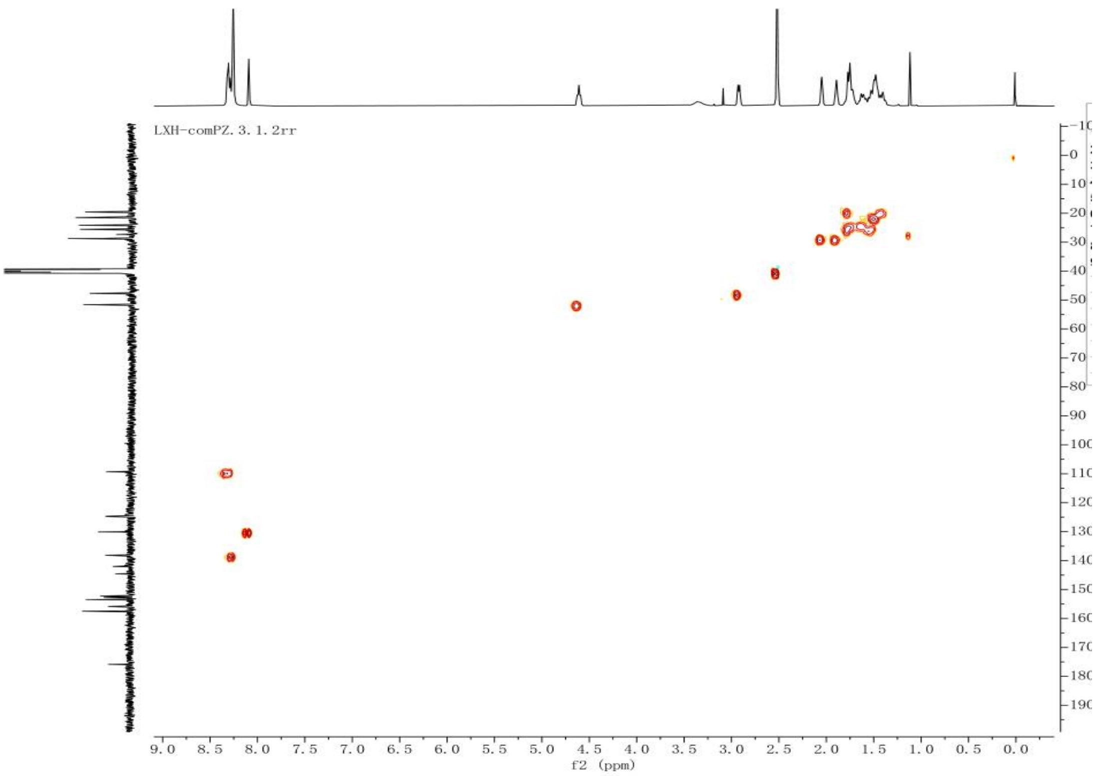

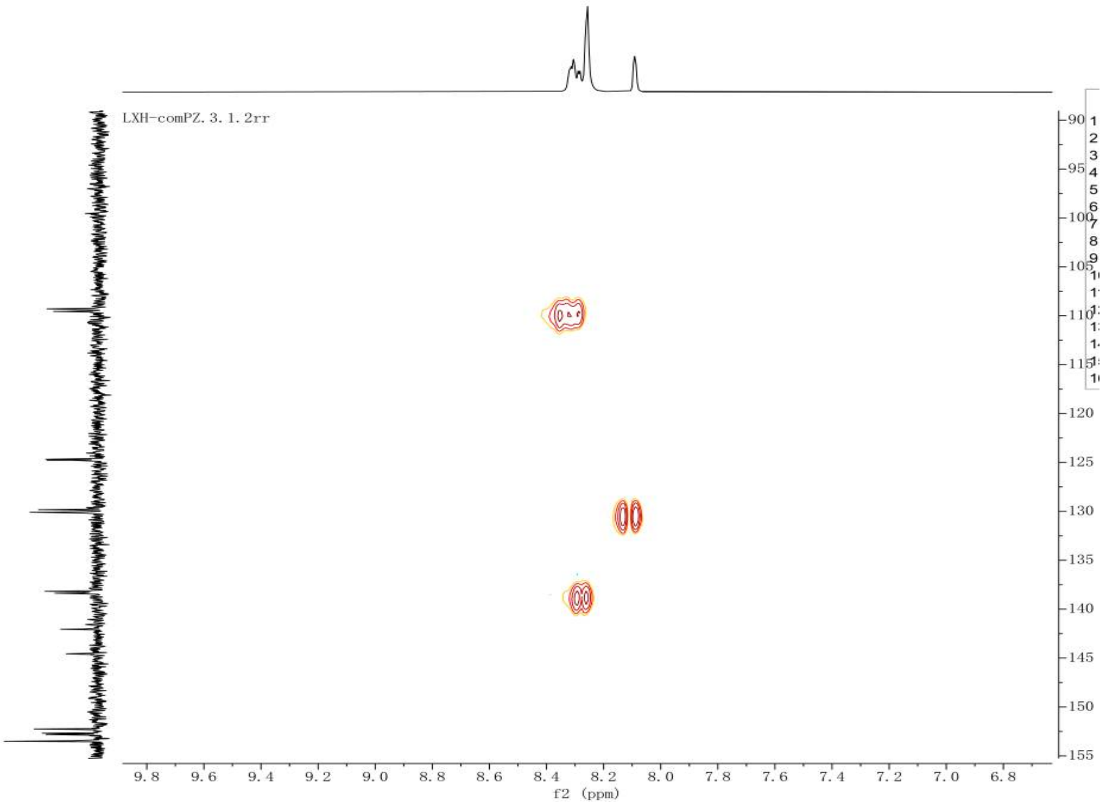
LXH-comPZ.3.1.2rr
f2 (ppm)

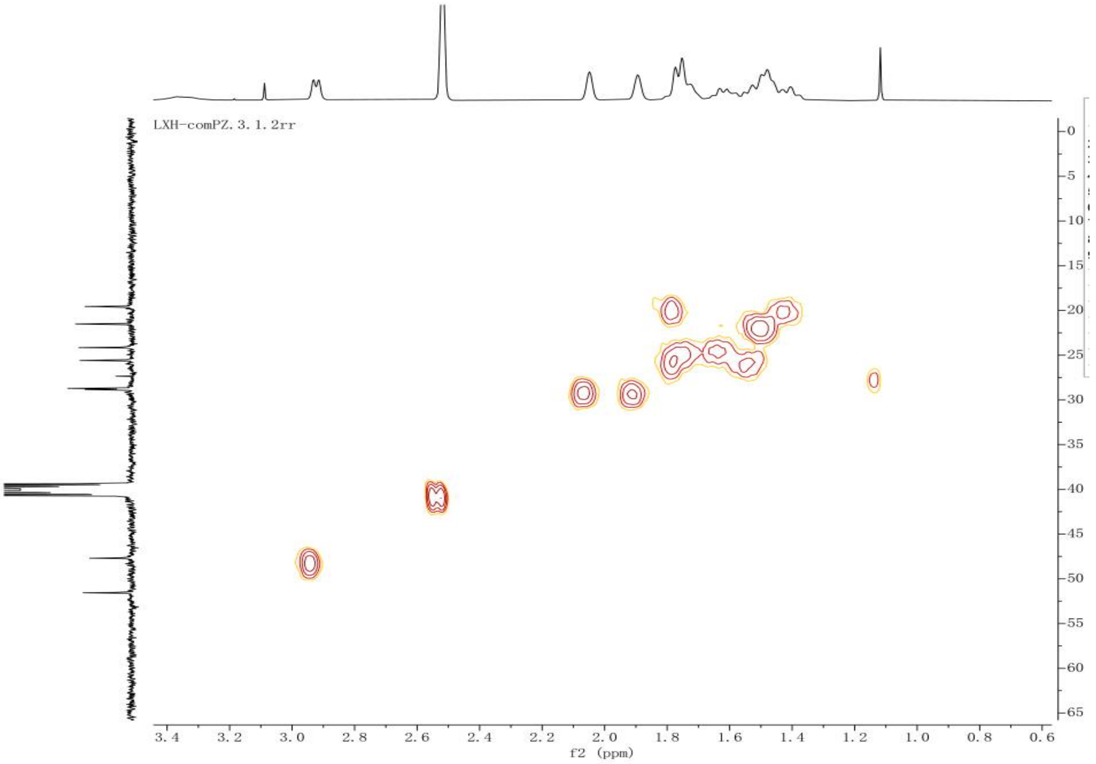
LXH-comPZ.3.1.2rr
f2 (ppm)

Figure S5. HPLC analysis of Comp. I and Comp. II

Experimental part：

HPLC： Agilent Technologies 1260 infinity

Column：Diamonsil C18 5μm 150 × 4.6 mm

Temperature：25 ℃

Flow rate：1.0 mL/min

Injection volume：5 μL

Sample concentration：1.0 mg/mL

Sample solvent： methanol

Gradient: 70% H_2O (with 0.5% HCOOH) and 30% CH_3CN over 20 min at a flow rate of 1 mL/min

Wavelength: 254 nm

Figure S5-1. Purity of Comp. I

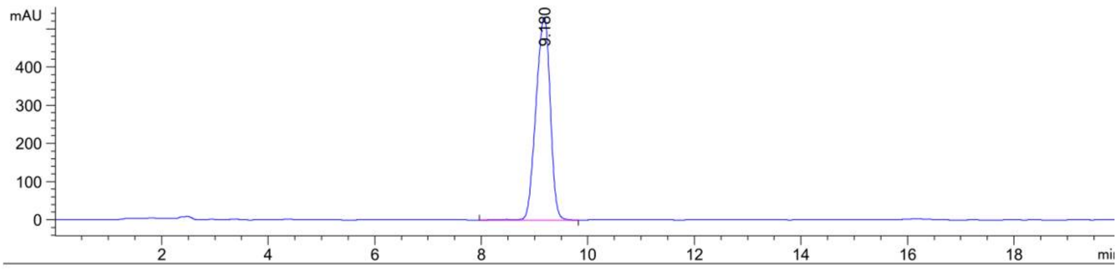

Purity: 100.0000%

Peak	Ret. Time(min)	Area(mAU*s)	Area%
1	9.180	1.00879e4	100.0000
Total		1.00879e4	100.0000

Figure S5-1. Purity of Comp. II

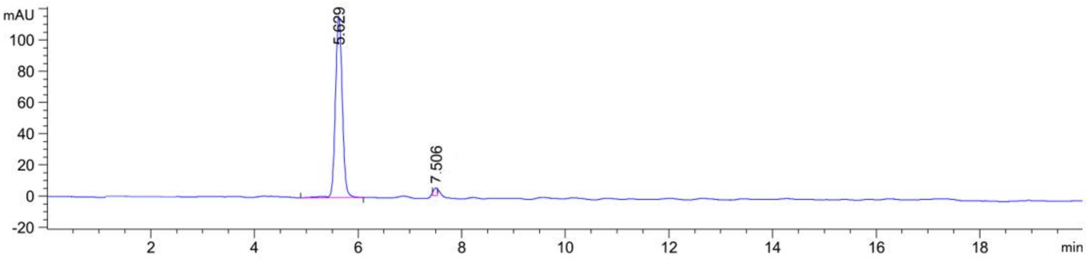

Purity: 97.8492%

Peak	Ret. Time(min)	Area(mAU)	Area%
1	5.629	1072.02051	97.8492
2	7.506	23.56424	2.1508
Total		1095.58475	100.0000

Figure S6. Chiral HPLC analysis of Compound 12

Experimental part：

Compound 12 (10 mg) was placed in a vial, to which 1 M aqueous NaOH (1 mL), toluene (1 mL), and benzoyl chloride (15 µL) were added. The mixture was allowed to stand for 15 min at room temperature, during which N-benzoylation proceeded to yield N-benzoyl-12. After separation, drying and evaporation of the toluene layer, the residue was dissolved in 2 mL a mixture of n-hexane and IPA (8:2) and the insoluble were removed by filtration. The filtrate (2 µL) was injected into the HPLC apparatus.

HPLC： Agilent Technologies 1260 infinity

Column：Daicel Chiralcel ODH column

Temperature：25 °C

Flow rate：1.0ml/min

Injection volume：5 µL

Sample concentration：15 mg/mL

Sample solvent： methanol

Gradient: 95% Hexane (with 0.5% HCOOH) and 5% IPA (0.1%DEA) over 15 min at a flow rate of 1.0ml/min

Wavelength: 220.4 nm

Purity: 99.3759%

Chiral purity (e.e.%): 98.7%

Figure S6-1. Chiral resolution of racemic compound 12

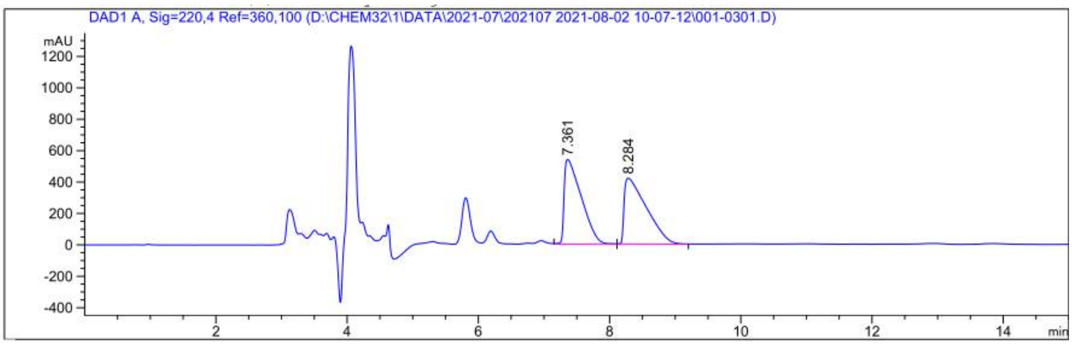


```
=====================================================================
                         Area Percent Report
=====================================================================

Sorted By             :      Signal
Multiplier            :      1.0000
Dilution              :      1.0000
Do not use Multiplier & Dilution Factor with ISTDs


Signal 1: DAD1 A, Sig=220,4 Ref=360,100

Peak RetTime Type  Width     Area      Height     Area
  #   [min]        [min]   [mAU*s]     [mAU]       %
----|-------|----|-------|----------|----------|--------|
   1   7.361 VB    0.2616 9757.93262  537.95270  49.7719
   2   8.284 BV    0.3165 9847.37207  418.56049  50.2281

Totals :                  1.96053e4   956.51318
```

Figure S6-2. Optical purity assessment Purity of compound 12

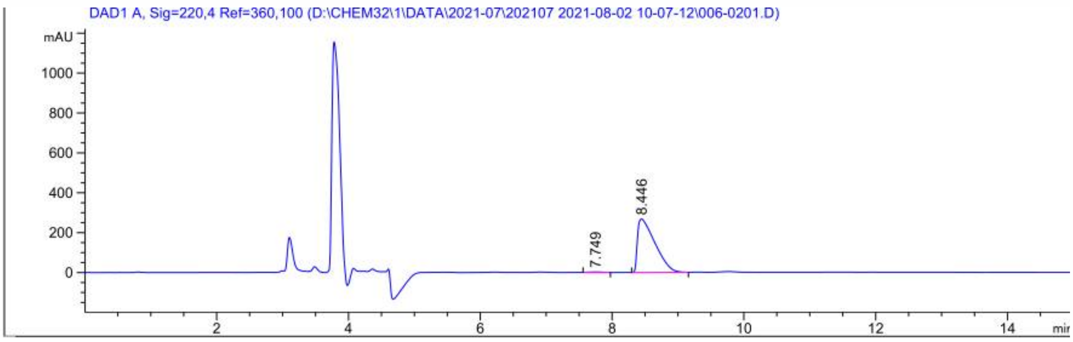


```
=====================================================================
                        Area Percent Report
=====================================================================

Sorted By             :      Signal
Multiplier            :      1.0000
Dilution              :      1.0000
Do not use Multiplier & Dilution Factor with ISTDs


Signal 1: DAD1 A, Sig=220,4 Ref=360,100

Peak RetTime Type  Width     Area      Height     Area
  #   [min]        [min]   [mAU*s]     [mAU]        %
----|-------|----|-------|----------|----------|--------|
   1   7.749 MM R  0.1894   31.00762    2.72798   0.6241
   2   8.446 BV    0.2549 4937.32568  268.10126  99.3759

Totals :                  4968.33330  270.82924
```
